# Learning shapes cortical dynamics to enhance integration of relevant sensory input

**DOI:** 10.1016/j.neuron.2022.10.001

**Published:** 2022-10-24

**Authors:** Angus Chadwick, Adil G. Khan, Jasper Poort, Antonin Blot, Sonja B. Hofer, Thomas D. Mrsic-Flogel, Maneesh Sahani

**Affiliations:** 1 Gatsby Computational Neuroscience Unit, University College London, London, UK; 2 Sainsbury Wellcome Centre for Neural Circuits and Behaviour, University College London, London, UK; 3 Institute for Adaptive and Neural Computation, School of Informatics, University of Edinburgh, Edinburgh, UK; 4 Centre for Developmental Neurobiology, King’s College London, London, UK; 5 Department of Physiology, Development and Neuroscience, University of Cambridge, Cambridge, UK

## Abstract

Adaptive sensory behavior is thought to depend on processing in recurrent cortical circuits, but how dynamics in these circuits shapes the integration and transmission of sensory information is not well understood. Here, we study neural coding in recurrently connected networks of neurons driven by sensory input. We show analytically how information available in the network output varies with the alignment between feedforward input and the integrating modes of the circuit dynamics. In light of this theory, we analyzed neural population activity in the visual cortex of mice that learned to discriminate visual features. We found that over learning, slow patterns of network dynamics realigned to better integrate input relevant to the discrimination task. This realignment of network dynamics could be explained by changes in excitatory-inhibitory connectivity among neurons tuned to relevant features. These results suggest that learning tunes the temporal dynamics of cortical circuits to optimally integrate relevant sensory input.

## Introduction

Cortical circuits process sensory information through both feedforward and recurrent synaptic connections (Lamme and Roelfsema, 2000). Feedforward connectivity can filter (Hubel and Wiesel, 1962; LeCun et al., 2015) and propagate (Abeles, 1991; Van Rossum et al., 2002) relevant information, allowing rapid categorization and discrimination of stimuli (Thorpe et al., 1996; Resulaj et al., 2018). However, the majority of synaptic input received by neurons in sensory cortex arises from neighboring cortical cells (Peters et al., 1994; Douglas et al., 1995), and recurrent cortical dynamics exerts a powerful influence on network activity during sensory stimulation (Fiser et al., 2004; Reinhold et al., 2015). The functional role of such recurrent synapses in the integration and transmission of sensory information remains poorly understood.

Many of the stimulus features represented in the spiking output of neurons in primary sensory cortex are already present in the net feedforward input they receive (Lien and Scanziani, 2013). Previous studies have proposed two possible functions of recurrent cortical synapses. First, recurrent synapses may increase the signal-to-noise ratio (SNR) of the relevant sensory features through selective amplification (Douglas et al., 1995; Ben-Yishai et al., 1995; Somers et al., 1995; Murphy and Miller, 2009; Liu et al., 2011; Li et al., 2013; Lien and Scanziani, 2013; Cossell et al., 2015). Second, recurrent synapses may enhance the efficiency of the encoding by suppressing redundant responses in similarly tuned cells (Olshausen and Field, 1996; Lochmann and Deneve, 2011; Chettih and Harvey, 2019). However, although recurrent amplification and competitive suppression can increase the SNR of single-neuron responses and improve coding efficiency, respectively, such mechanisms cannot increase the amount of sensory information transmitted through the network beyond the information that the network receives in its input (Cover and Thomas, 2006; Seriès et al., 2004; Beck et al., 2011; Kanitscheider et al., 2015; Zylberberg et al., 2017; Huang et al., 2022).

Recent studies have shown that visual features such as orientation become easier to decode from both single-cell and population responses in primary visual cortex (V1) when mice and monkeys learn to associate them with behavioral contingencies (Poort et al., 2015; Khan et al., 2018; Jurjut et al., 2017; Yan et al., 2014). This apparent improvement in representation is accompanied by changes in functional interactions among excitatory and inhibitory cell types within the local circuit (Khan et al., 2018). Since changes in recurrent amplification or competitive suppression cannot increase the total available information, it remains unclear how changes in cortical connectivity could generate the observed improvements.

Here, we ask whether improvements in stimulus decodability over learning could arise through selective temporal integration of relevant feedforward sensory input. We first show analytically how the output of a network can be tuned to optimally discriminate pairs of input stimuli by matching its recurrent dynamics to their sensory input statistics. In particular, we show that a stimulus decoder applied to network output performs best if the dimension of network input with greatest SNR activates a pattern of recurrent network dynamics that decays slowly. We then study how the dynamical properties of neural circuits in mouse V1 change as animals learn to discriminate visual stimuli. Using a dynamical systems model fit to experimental data (Khan et al., 2018), we find that slowly decaying patterns in the recurrent dynamics became better aligned with high-SNR sensory input over learning. Finally, we analyze circuit models with excitatory and inhibitory neurons to explore how this alignment might arise through changes in the circuit. We find that stimulus-specific changes in connectivity between excitatory and inhibitory neurons increase the alignment of recurrent dynamics with sensory input as observed experimentally. These connectivity changes predict changes in stimulus tuning and cell type-specific reorganization of dynamics within the model, which we find to be recapitulated in the experimental data. Our findings suggest a critical role for cortical dynamics in selective temporal integration of relevant sensory information.

## Results

### Sensory discrimination relies on temporal integration of optimally weighted sensory input

We first asked how the dynamical properties of a recurrent network influence its capacity to discriminate sensory inputs. The scenario we considered had one of two possible stimuli appear for the duration of a trial. Each stimulus generated an input to each neuron in the network with constant mean corrupted by additive, temporally uncorrelated, Gaussian noise (this approximates the net feedforward synaptic input a neuron receives from a large number of upstream neurons; see Stein, 1967; Capocelli and Ricciardi, 1971; Lansky, 1984). To determine how these inputs should be integrated for optimal discrimination performance, we adopted a signal processing perspective (see Methods S1 File).

**Figure 1:**
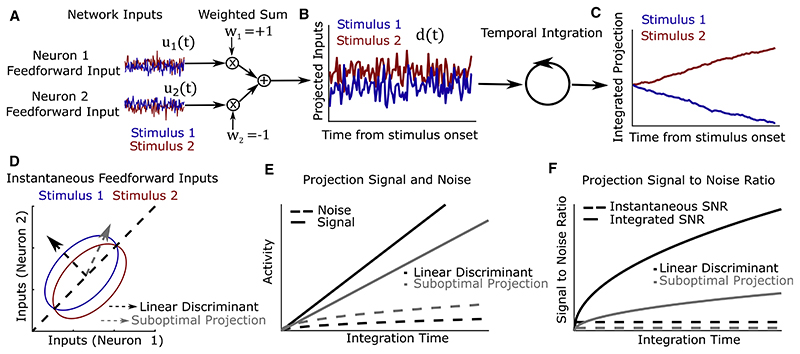
Stimulus discrimination performance depends on temporal integration of weighted sensory input (A) Feedforward inputs to a two-neuron network, shown for two different stimuli (red and blue). (B) A weighted sum (linear projection) of the instantaneous inputs shown in (A). (C) The temporally integrated input projection for each stimulus (cumulative sum of projected inputs shown in B). (D) Distributions of instantaneous feedforward input for each of the two stimuli (colored ellipses), their optimal linear discriminant (dashed black arrow), and a second suboptimal projection (dashed gray arrow). (E) The signal (difference in mean; solid lines) and noise (standard deviation; dashed lines) of activity following linear projection and temporal integration, shown for the two projections in (D). (F) The instantaneous (dashed) and temporally integrated (solid) signal-to-noise ratio of these two projections.

Two noisy stimuli can be optimally discriminated from the instantaneous sensory input to the network by taking a one-dimensional linear combination of the inputs to different neurons (Figures 1A and 1B) weighted according to the “linear discriminant.” This is the linear combination of inputs that achieves the best compromise between separating the mean inputs under the two stimuli and avoiding projected noise (Figure 1D, black dashed arrow). Writing **u**(*t*) for a vector collecting the inputs to all neurons at time *t*, the linear discriminant is a vector **w** of the same dimension such that the projected input vector *d*(*t*) = **w**·**u**(*t*) has the greatest possible SNR_input_(**w**) for the discrimination of the two stimuli (Figures 1B and 1D). Then, to discriminate stimuli over a window of duration *T*, the optimal strategy is simply to integrate the linear discriminant projection across the time window (Figure 1C), yielding an output with ${\text{SNR}}_{\text{output}}={\text{SNR}}_{\text{input}}\left(\text{w}\right)\sqrt{T}$ (Figures 1E and 1F).

These results demonstrate that a network can best generate distinct activity patterns in response to two different continuous stimuli if it temporally integrates the input stimuli weighted according to their projection onto an optimal linear discriminant.

### Recurrent networks enhance sensory discrimination by alignment of slowly decaying dynamical modes with optimal sensory input

How might this optimal discrimination function be achieved using a recurrent network? To address this, we considered how noisy stimulus input is filtered through the recurrent network dynamics. A core feature of recurrent networks is their capacity to generate multiple distinct activity patterns, which may unfold with different dynamical time constants within the network’s high-dimensional activity space (Rabinovich et al., 2006; Miller, 2016; Sussillo et al., 2014). We asked if these different time constants of network dynamics could allow a network to act as an optimal integrator of sensory input by providing windows of temporal integration over the optimal input discriminant (Goldman et al., 2009a).

**Figure 2:**
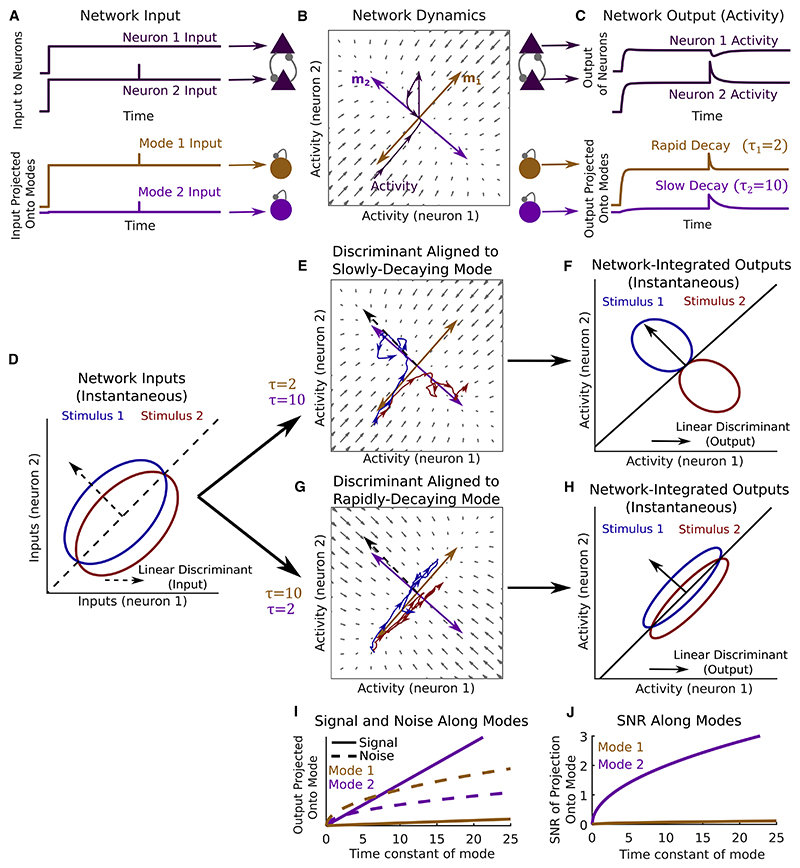
Alignment of dynamical modes with feedforward input determines sensory discrimination performance (A–C) Illustration of a two-neuron network receiving feedforward input and generating an output activity pattern with rapidly and slowly decaying dynamical modes (brown and light purple). (A) (Top) Constant input to each neuron, and a small input perturbation to neuron 2. (Bottom) The same input shown following projection ontothe two modes of network dynamics. (B) Illustration of network dynamics. Gray arrows depict the dynamical flow of network activity from a given statewhen input is held at the constant level shown in (A). Light purple and brown arrows depict modes’ activation patterns **m**. Thetrajectory of neural activity in responsetothe input in (A) is shown in dark purple. The input perturbation to neuron 2 generates a dynamical response along both modes, each decaying with a different time constant *τ*. (C) Network output shown for each neuron and along each mode. Single-neuron responses exhibit complex and heterogeneous time courses, but the network response projected onto any mode exhibits a simple exponential decay. (D) Distributions of instantaneous feedforward input under two different stimuli (red and blue ellipses), as in Figures 1A and 1D (note that inputs have time-varying noise). (E) A network with a slowly decaying mode aligned to the input linear discriminant. Blue and red traces show example trajectories of network output when the network is driven by a single-trial input from each of the two stimulus distributions. (F) Distributions of instantaneous network output at equilibrium under each stimulus. (G and H) As in (E) and (F) but with a rapidly decaying mode aligned to the input linear discriminant. (I) Signal and noise of instantaneous network output along each mode, as a function of the mode’s time constant. (J) Signal-to-noise ratio of instantaneous network output along each mode. Note that (A)–(H) show a special case of orthogonal modes, i.e., normal dynamics. The more general case of non-normal dynamics is shown in Figure S1.

For networks that settle into a steady pattern of firing rates when driven by a constant input (Figures 2A and 2C), the behavior of small fluctuations around that input-driven fixed point can be approximated with a linear dynamical system (Figure 2B). The dynamics of this linearized network can be described by a set of dynamical “modes,” each of which associates a time constant *τ* with a unique pattern of network activation **m** (Figure 2B). The activation pattern **m** is a vector describing a particular deviation of network activity from the fixed point, with elements equal to the relative deviation of each neuron, whereas *τ* determines the time taken for an activity fluctuation along **m** to decay back toward the fixed point through the network dynamics. In particular, when network activity is perturbed away from its input-driven fixed point along any direction, the ensuing population activity trajectory projected onto any given mode’s **m** decays as an exponential function with the corresponding time constant *τ* (Figures 2B and 2C). Moreover, when the network is driven by a stimulus input with continuously fluctuating noise as considered here (Figure 1A), population activity projected onto any mode’s **m** behaves as a leaky integrator, with each mode independently aggregating inputs that fall along its activation pattern with an integration window of duration *τ* (Figures 2D and 2E). In the discrimination task, input associated with one of the two possible stimuli drives the network on any given trial (Figures 1A, 1D, and 2D). In this case, provided that the two stimulus-driven fixed points are sufficiently close to fall within the domain of network linearization (Figures 2E and 2F), the SNR of network output projected onto any single mode’s **m** following network integration matches the signal processing solution above, with ${\text{SNR}}_{\text{output}}\left(\text{m}\right)={\text{SNR}}_{\text{input}}\left(\text{m}\right)\sqrt{2\tau }$ (Figures 2I and 2J). Thus, a recurrent network can achieve the optimal strategy for stimulus decoding (Figure 1) if its recurrent connectivity gives rise to a dynamical mode with activation pattern **m** that is aligned to the input linear discriminant **w** (i.e., **m** = **w**) and decay time constant *τ* that is longer than the stimulus window *T* (as in Figures 2E and 2F; Figures 2G and 2H show suboptimal integration). In other words, the recurrent dynamics are optimized for discrimination of a pair of input stimuli with linear discriminant **w** if fluctuations of network activity along **w** decay slowly.

Biological neural networks exhibit complex “non-normal” dynamics which may rapidly amplify network input and produce temporally extended “functionally feedforward” network responses (Ganguli et al., 2008; Murphy and Miller, 2009; Goldman, 2009b). In such networks, activation of one network activity pattern causes subsequent activation of other activity patterns, leading to transient activity sequences whose lifetime exceeds the decay time of any individual mode (Goldman, 2009b). We asked whether these non-normal dynamics might yield further mechanisms for optimizing stimulus discrimination. We found analytically that the discrimination performance of a network depends on the geometry of its modes’ activation patterns (Figures S1A and S1B). When these are orthogonal, corresponding to “normal” networks, response information is maximized when the most slowly decaying mode has its activation pattern aligned to the input linear discriminant (Figures 2E, S1A, and S1B). Analyzing non-normal networks, we found that response information further improves when multiple modes have their activation patterns aligned with the input linear discriminant (Figures S1A and S1B). These improvements arise through functionally feedforward dynamics, which increase the total window of network integration relative to the decay time constants of the individual modes (Figures S1A and S1E–S1J) (Ganguli et al., 2008; Goldman, 2009b).

A surprising consequence of this analysis is that networks which optimally integrate their input tend to exhibit strong information-limiting correlations (Figures S1B–S1D; Moreno-Bote et al., 2014). This phenomenon occurs in both normal and non-normal networks and can be understood intuitively by considering the effect of temporal integration on the mean and trial-by-trial variability of responses: as temporal integration of the input discriminant is increased, response variability along the direction separating the two stimuli increases, but the mean responses to the two stimuli diverge at a faster rate, leading to increased stimulus discriminability despite increased information-limiting correlations (Figures 2D–2F, 2I, 2J, S1A, and S1B). Thus, strong information-limiting correlations are a signature of optimal integration of sensory input through recurrent network dynamics.

Taken together, our findings demonstrate that recurrent networks maximize their capacity to discriminate sensory inputs when they align one or more slowly decaying modes of dynamics with the optimal input discriminant. We reasoned that such a mechanism may underlie improvements in cortical representations for relevant stimuli over learning (Poort et al., 2015; Khan et al., 2018).

### Learning reorganizes cortical networks to enhance integration of relevant sensory input

**Figure 3:**
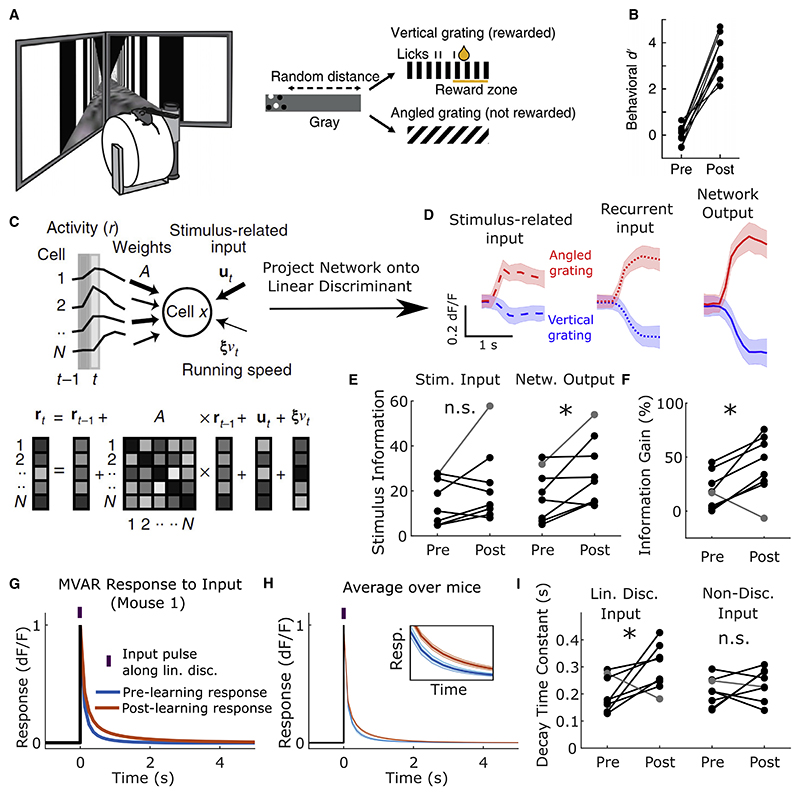
Changes in V1 population dynamics over learning selectively enhance temporal integration of relevant sensory input (A) Visual discrimination task. (B) Behavioral performance of each mouse pre- versus post-learning. (C) Schematic describing MVAR model fit to imaged population activity. The MVAR model fits variability in single-trial responses of each cell by estimating the contribution of stimulus-locked input, recurrent input from the local cell population, and running speed. (D) The inferred stimulus-related and recurrent input and the imaged network output, each projected onto the optimal linear discriminant (mean ± SD over trials for one mouse post-learning). (E) Information in MVAR stimulus-related input and network output for each mouse pre- versus post-learning (gray line delineates a particular mouse whose improvements occurred through enhanced stimulus-related input). (Input information p > 0.36, output information p = 0.035, one-sided sign tests on N = 8 mice). (F) MVAR input-output information gain, pre- versus post-learning for each mouse. (p = 0.035, one-sided sign test on N = 8 mice). (G) Simulated response of the MVAR model to a synthetic pulse of input aligned to the linear discriminant, pre- and post-learning for one mouse. (H) As in (G), showing mean ± SEM over mice. Inset shows zoomed in traces. (I) Left: the decay time constant of responses in (G) and (H) for each mouse, pre- versus post-learning. Right: the decay time constants for a second input pattern that carries no information about stimulus identity. (Discriminant input p = 0.035, non-discriminant input p = 0.64, one-sided sign tests on N = 8mice). See also Figures S2–S4.

With this description of recurrent processing in mind, we examined the effects of learning on cortical dynamics and sensory representations. We analyzed the activity of neuronal populations in primary visual cortex of head-fixed mice as they learned to perform a visual discrimination task within a virtual reality environment. Over a period of 7-9 days, mice learned to selectively lick a reward spout in a virtual corridor lined with vertical but not angled stripes (Figures 3A and 3B). The responses of the same populations of neurons to these stimuli were measured before and after learning using chronic two-photon calcium imaging. Learning led to an improvement in the linear discriminability of these two stimuli based on instantaneous population responses (Figure 3E right, p = 0.035, one-sided sign test on pre- versus post-learning linear Fisher information; see STAR Methods for details). Given that instantaneous sharpening or amplification of sensory input by the V1 circuit cannot increase response information (Cover and Thomas, 2006; Zamir, 1998; Series et al., 2004; Beck et al., 2011), we hypothesized that such improvements could arise via either (1) an increase in sensory information provided through external input to the circuit (i.e., an increase in SNR_input_(**w**) caused by changes in upstream processing) or (2) a reorganization of cortical circuit dynamics to enhance temporal integration of sensory input (Figures 1 and 2).

To address these hypotheses, we first asked whether mouse behavior or neural activity showed signatures of temporal integration. As predicted by the temporal integration hypothesis, reaction times were slower on hit trials than false alarm trials (p < 10^−16^, Wilcoxon rank sum test, median lick time on hit/false alarm trials 1.24 and 0.87 s) and error rates decreased as a function of time from stimulus onset (Figures S2A and S2B). Moreover, stimulus discriminability based on instantaneous population responses increased over the course of a trial (Figure 3D, right; Figure 7C), and network responses along the linear discriminant ramped toward the vertical stimulus before licking on false alarm trials (Figure S2C) and exhibited slower autocorrelations after learning than before (Figures S2D and S2E), consistent with an increased integration timescale along the discriminant. These findings provide neural and behavioral evidence for the temporal integration hypothesis. However, they do not exclude changes in sensory input or distinguish among alternative dynamical mechanisms (e.g., Figures 2 and S1), which we next sought to investigate.

Distinguishing among these possibilities requires a complete characterization of the dynamics of the imaged circuit and the sensory input it receives before and after learning. As it is not currently possible to achieve this experimentally, we sought to infer the recurrent dynamics and stimulus inputs which best accounted for the coordinated activity patterns of the imaged circuit using a statistical model fit to the data. To this end, we examined a multivariate autoregressive (MVAR) linear dynamical system model we had previously fit to population activity imaged before or after learning (Khan et al., 2018). The MVAR model predicts the activity of each cell at imaging frame t based on (1) recurrent input from all imaged cells at time step t-1, with stimulus-independent weights; (2) a time-varying stimulus-dependent input, locked to stimulus onset and the same for all trials with a given stimulus; and (3) the running speed of the animal at time t (Figure 3C). Imaged responses in the population covaried in time and across trials, in a way that could not be explained by changes in the stimulus or changes in running behavior (Khan et al., 2018). The model depended on the recurrent interaction term to capture such “noise” covariance, and hence, once the model was fit to data, these weights were effectively determined by the structure of observed trial-by-trial variability. Conversely, the stimulus-dependent trial-invariant terms were determined during fitting so that the input signals, once fed through the recurrent terms of the model, captured the trial-averaged response profiles. Any remaining trial-by-trial variability in the data was assigned to a residual term (see STAR Methods and Khan et al., 2018 for a detailed discussion of the MVAR model and its validation on the present dataset). Given this characterization of the imaged responses in terms of stimulus-related input and recurrent interactions (Figure 3D), we then sought to determine the respective contributions of these components to the improvements in response information over learning (Figure 3E right).

To assess whether input information increased over learning, we computed the linear discriminability of stimuli based on the stimulus-related input inferred by the MVAR model, assigning model residuals to noise in this input (Figure 3D, left). Information contained in this input did not increase (p = 0.36, one-sided sign test on linear discriminability pre- versus post-learning over all mice; Figure 3E, left). However, there was an increase with learning in the gain of output over input information for 7/8 mice (Figures 3E and 3F, p = 0.035, one-sided sign test on relative percentage difference between MVAR input and output information). Thus, the MVAR model ascribed improvements in population response information to learning-related changes in recurrent interactions acting on stimulus-related input that was itself unchanged in information content.

If these recurrent changes acted to improve temporal integration, then the network response to an input pattern aligned with the linear discriminant should be observed to decay more slowly after learning than before. Indeed, the MVAR response to a pulse of such input decayed more slowly after learning for all mice in which improvements in response information were attributed to recurrent dynamics (p = 0.035, one-sided sign test on all mice, Figures 3G–3I). Moreover, when this analysis was repeated for an input pattern that was orthogonal to the input discriminant, the decay time did not change over learning (p = 0.64, one-sided sign test, Figure 3I right; Figure S3A). Thus, learning induced changes in temporal integration which were selective for task-relevant sensory input.

Enhanced temporal integration could arise through changes in the interaction weights or the stimulus-related input (for example, if stimulus input realigned to drive more slowly decaying network activity patterns). To distinguish between these possibilities, we refit the MVAR model with either interaction weights or stimulus-related input constrained to remain fixed over learning (see STAR Methods). Changes in temporal integration did not occur when interaction weights were fixed (p = 0.36, one-sided sign test) but persisted when stimulus-related input was fixed (p = 0.004, one-sided sign test, Figures S3B and S3C). This suggested that the improvements relied on changes in interaction weights but not stimulus input.

Motor signals such as running and licking are known to modulate responses in visual cortex (Niell and Stryker, 2010; Musall et al., 2019; Stringer et al., 2019). Thus, a possible explanation for our findings is that stimulus-locked changes in motor behavior drive changes in cortical responses, which are misconstrued as changes in recurrent dynamics by the MVAR model. We tested this hypothesis using an MVAR model which included an additional lick-dependent input and in which both velocity and licking coefficients were free to change with learning, allowing not only for changes in motor behavior to drive changes in activity through fixed coefficients but also for possible effects of changes in coupling of neural activity to these motor signals (see STAR Methods). Even in this more flexible model, we found that the running and licking contributions to population activity along the linear discriminant were negligible both before and after learning (Figure S3H). Moreover, repeating key analyses using this more flexible model did not alter our results (Figures S3I-S3L). Thus, changes in recurrent integration with learning could not be explained by stimulus-locked changes in motor behavior.

Taken together, these findings suggest that stimulus information in network responses improved over learning through changes in recurrent dynamics that selectively enhanced temporal integration of task-relevant sensory input.

### Enhanced integration depends on realignment of slowly decaying modes with sensory input

**Figure 4:**
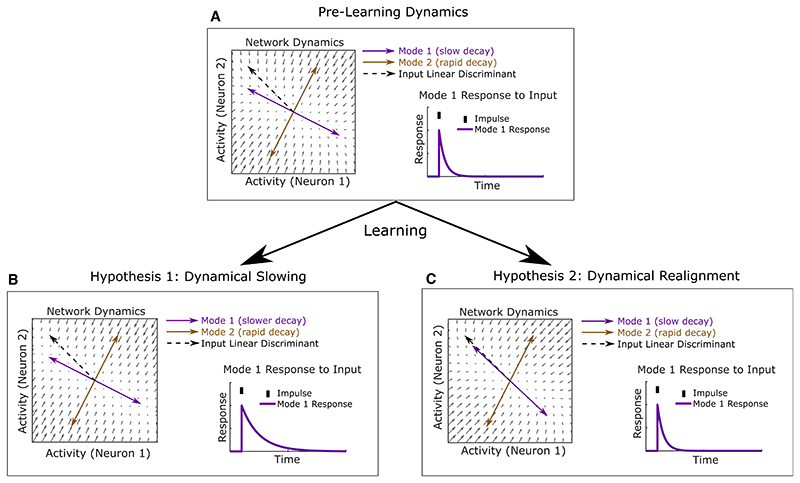
Improvements in temporal integration of relevant sensory input could arise from either slowing or realignment of dynamical modes (A) Example of pre-learning dynamics for a two-neuron network. (B) According to the dynamical slowing hypothesis, modes whose activation patterns are best aligned with the input linear discriminant extend their decay time constants over learning, leading to longer timescales of integration over the relevant input patterns. (C) In the dynamical realignment hypothesis, modes which decay most slowly become better aligned to the input linear discriminant over learning.

Altered recurrence could selectively enhance temporal integration of relevant sensory input in two ways. First, it could lengthen the decay time constants of those modes whose activation patterns are already best aligned with the input linear discriminant (“dynamical slowing hypothesis,” Figures 4A and 4B). Alternatively, it could realign the activation patterns of existing slowly decaying modes toward that discriminant (“dynamical realignment hypothesis,” Figure 4C).

**Figure 5:**
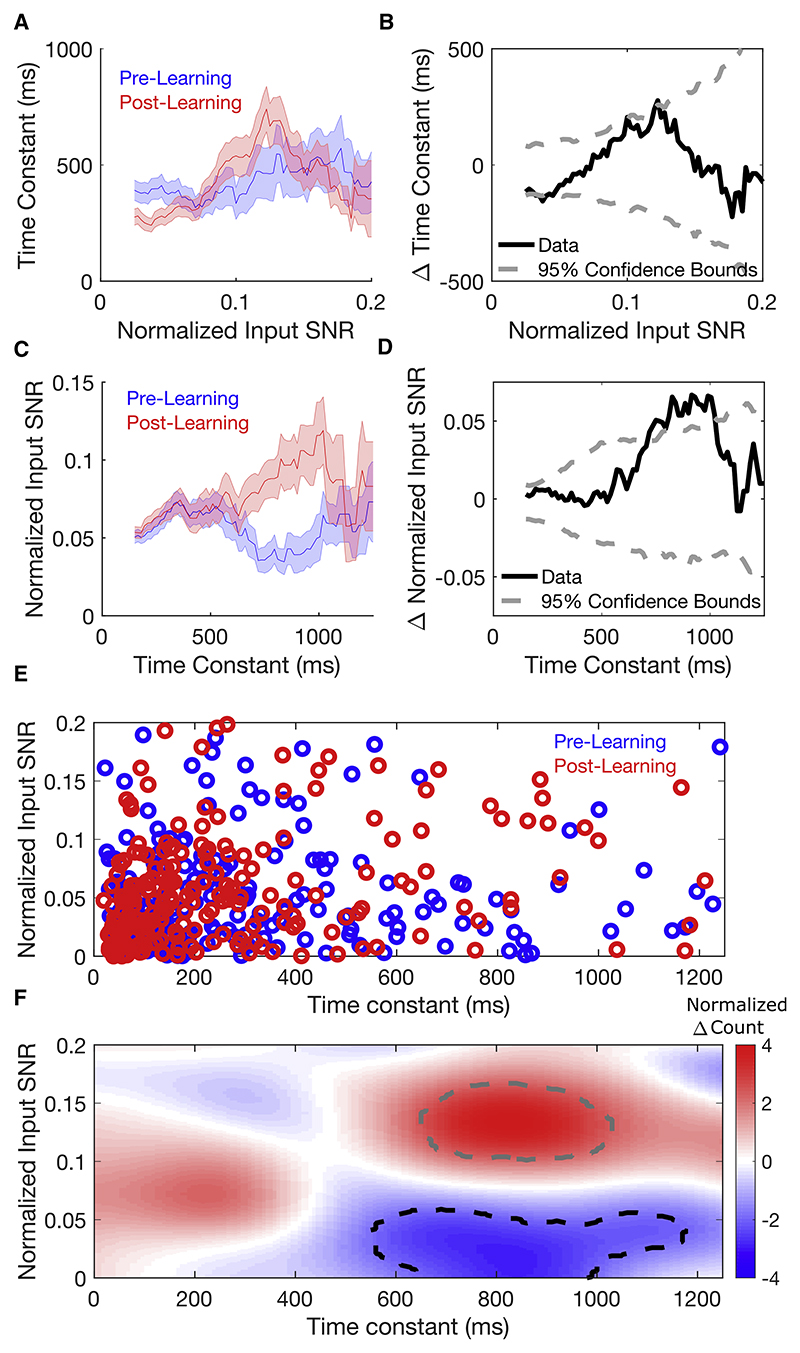
The MVAR model supports the dynamical realignment hypothesis but not the dynamical slowing hypothesis (A) Dependence of the time constants of modes on their input SNR, pre- and post-learning (average time constant conditioned on normalized input SNR, mean ± SEM taken over pooled modes over animals). (B) Difference between pre and post curves in (A) (solid black line). Dashed gray lines show 2.5% and 97.5% of shuffled distributions. (C and D) As in (A) and (B) but for an average of normalized input SNR conditioned on time constant. (E) Time constants and normalized input SNRs of modes pooled over animals pre- and post-learning. (F) Smoothed histogram of difference over learning in numberof modes with a given input SNR and time constant (normalized by standard deviation over shuffles). Dashed black and gray lines show regions where the number fell below 2.5% and above 97.5% of shuffled distributions, respectively (see STAR Methods). See also Figures S2–S4.

To distinguish between these two hypotheses, we computed modes of network dynamics and their time constants from the pre- and post-learning MVAR interaction weight matrices. For each mode, we computed the proportion of stimulus-related input information that fell along its activation pattern (its “normalized input SNR,” SNR_norm_(**m**) = SNR_input_(**m**)/SNR_input_(**w**), which is maximized when the mode is aligned to the input linear discriminant). The dynamical slowing hypothesis predicts that the time constants of modes with high input SNR should increase (Figures 4A and 4B). However, the time constants of modes did not change significantly over learning, either across all modes (p = 0.79, one-sided Wilcoxon rank sum test on pre- versus post-learning time constants for all modes pooled across animals) or the subset of modes with high input SNR (Figures 5A, 5B, and S3J). In contrast, the dynamical realignment hypothesis predicts that the normalized input SNRs of slowly decaying modes should increase (Figures 4A and 4C). This prediction was borne out by a striking increase over learning in normalized input SNR (p = 0.03, one-sided Wilcoxon rank sum test on all modes pooled across animals pre- versus post-learning) which was most pronounced for modes with time constants of ~ 700—1,000 ms (Figures 5C, 5D, and S3K). The range of time constants for which input SNR increased was consistent with the time-scale at which response SNR and behavioral performance increased (Figures 3D, 7C, and S2B). The increase in normalized input SNR occurred for 7/8 mice (p = 0.035, one-sided sign test on average over modes within each mouse pre- versus post-learning, Figure S3D), whereas time constants increased for only 3/8 mice (p = 0.86, one-sided sign test on average over modes within each mouse pre- versus post-learning, Figure S3E). Examining the joint distribution of the time constants and normalized input SNRs of modes before and after learning (Figures 5E, 5F, and S3L), we found a fall in the number of slowly decaying modes with low input SNR matched by an increase in the number with similar decay time constants but high input SNR. These changes are consistent with a realignment of slowly decaying modes toward the input linear discriminant.

Signatures of dynamical realignment could also be detected through non-MVAR based analyses of the data. First, the response SNRs before and after learning were related by a multiplicative scaling, as predicted by dynamical realignment but not slowing of modes (Figure S2F). Second, principal component analysis revealed slowly varying population modes whose time course did not change substantially with learning but whose neuronal activation pattern became better aligned to the response discriminant (Figures S2G and S2H). These findings further reinforce the conclusion that network dynamics realign with learning to optimally integrate task-relevant sensory input.

In principle, enhanced integration could also arise through greater non-normality in the recurrent dynamics (Figure S1). However, we found that for 6/8 animals the recurrent dynamics became less non-normal over learning (p = 0.03, two-sided Wilcoxon rank sum test), suggesting that this mechanism did not contribute to the enhancements detected in the MVAR model (Figures S3F and S3G). Thus, changes in non-normality of dynamics did not account for improvements in integration with learning.

Our dataset comprised multiple molecularly distinct cell types, which were simultaneously imaged before and after learning (pyramidal [PYR], parvalbumin [PV], somatostatin [SOM], and vaso-intestinal peptide [VIP] expressing, see Khan et al., 2018). We next sought to determine whether improvements in integration in the MVAR model relied on cell type-specific changes in sensory input or recurrent dynamics. To test whether learning modified the relative contribution of different cell classes to the population-level representation of task-relevant stimuli, we computed the total loading of each cell class onto the linear discriminant before and after learning (Figure S4A, loading was defined as the proportion of the length of the discriminant vector that was generated by a given cell class, normalized by the number of cells in that class). There were no statistically significant changes in discriminant loading of any cell class with learning, suggesting that learning did not alter the distribution of population information across cell classes (note that this is not inconsistent with the differential improvements in single-cell response SNR found in Khan et al., 2018, as these may be offset at the population level by changes in noise correlations). However, there was a cell type-specific reorganization of the network response to task-relevant input perturbations, consistent with the hypothesis that improvements in integration are caused by changes in dynamical interactions among distinct cell classes (Figure S4B). Moreover, PV neurons coupled more weakly into the high-SNR modes that emerged after learning than the low-SNR modes that disappeared with learning (Figures S4C and S4D). This suggested that changes in PV cell response dynamics, but not SOM or VIP, were important for learning-related improvements in V1, consistent with the changes in PV functional interactions and stimulus selectivity found by Khan et al. (2018) (see also Figure S7B).

In summary, these results support the hypothesis that learning reorganizes cortical dynamics in order to align slowly decaying modes of recurrent dynamics with the optimal linear discriminant of sensory input (Figure 4C), thereby enhancing temporal integration of task-relevant sensory information.

### Stimulus-specific but not uniform connectivity changes reproduce the changes in dynamical integration observed in the MVAR model

**Figure 6:**
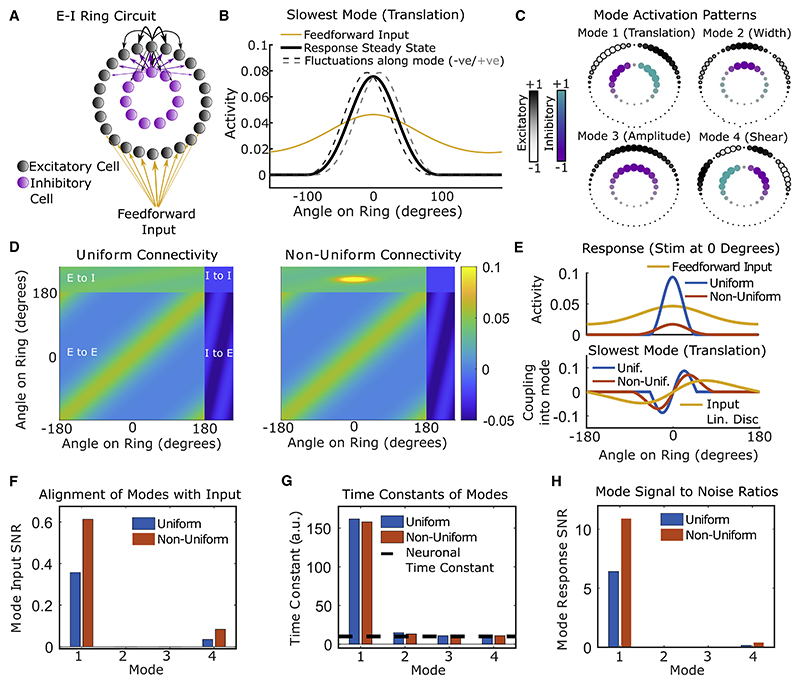
Stimulus-specific inhibition aligns the slowest-decaying mode with the input linear discriminant (A) Excitatory-inhibitory ring network model for V1 orientation selectivity. (B) Steady-state network response (solid black) and perturbations along the most slowly decaying mode (dashed gray). Feedforward input (yellow) was rescaled for aid of visual comparison. Only excitatory cells are shown. (C) Activation patterns **m** for the four most slowly decaying modes (in order of time constant). Size and color of circles depicts weighting of cell in mode activation pattern (see B and E bottom, and Figure S5 for alternative visualizations). (D) Synaptic weight matrix for a ring network with uniform (left) and non-uniform (right) connectivity. (E) (Top) Feedforward input and steady-state responses for the two networks. (Bottom) The most slowly decaying mode **m** for each of the two networks, overlaid with the input linear discriminant. The greater overlap between red and yellow lines compared with cyan and yellow indicates increased alignment. (F-H) Input SNRs (F), time constants (G), and response SNRs (H) for the four most slowly decaying modes. See also Figures S5–S7.

How might the dynamical realignment observed in the MVAR model relate to systematic changes in synaptic connectivity and response tuning within the V1 circuit? Constraints in the original experiment meant that we were unable to determine the orientation tuning of the imaged neurons. Thus, we turned to a canonical circuit model for feature selectivity to investigate the relationship between network connectivity, tuning curves, and dynamical modes (Ben-Yishai et al., 1995; Rubin et al, 2015; Hennequin et al., 2018). The model comprised excitatory and inhibitory neurons arranged on a ring corresponding to their preferred orientation before learning. Neurons at nearby locations formed stronger synaptic connections and received more similarly tuned feedforward input than those more separated around the ring (Figure 6A). This is consistent with local microcircuits in visual cortex in which neurons receive feature-tuned feedforward input (Lien and Scanziani, 2013) and interact through feature-specific local synapses (Cossell et al., 2015; Znamenskiy et al., 2018).

We first analyzed the tuning curves and modes of dynamics in the E-I ring network. The network formed a stable bump of activity centered on the stimulus orientation (Figure 6B, solid black line), and each of the four most slowly decaying modes reflected an interpretable fluctuation about this stable activity pattern: side-to-side translation (Figure 6B, dashed gray lines), sharpening/broadening, gain of amplitude, and asymmetric shear (Figures 6C and S5A–S5C). Responses were sharpened relative to feedforward input (Figure 6B, black versus yellow line) and the degree of sharpening depended on the strength and tuning of excitatory and inhibitory synapses around the ring (Figures S5D–S5F). We asked whether changes in recurrent connectivity that act to sharpen network responses could account for the reorganization of dynamical modes observed in the MVAR model. We found that connectivity changes that increased recurrent sharpening also reduced alignment of the slowest dynamical mode with the input linear discriminant, in contrast to the increased alignment observed in the MVAR model (Figures S5G–S5L). This relationship between sharpening and alignment of modes persisted over a broad range of networks with varying strength and feature-tuning of excitatory and inhibitory synaptic weights (Figures S6A–S6D). Thus, uniform changes in the strength or tuning of synaptic weights did not reproduce the realignment of modes with learning observed in the data.

**Figure 7:**
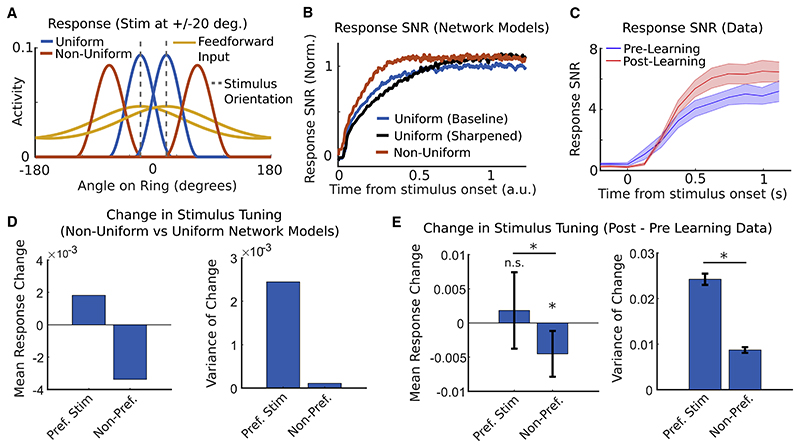
Stimulus-specific inhibition predicts observed changes in stimulus tuning (A) Network responses to two stimulus orientations separated by 40°. (B) SNR of instantaneous network output for three networks (based on simulation of nonlinear dynamics). (C) SNR of imaged V1 population responses (mean ± SEM over mice). (D) The change in responses of excitatory neurons to their preferred and non-preferred stimuli induced by non-uniform inhibition (mean and variance over cells). The greater variance for the preferred stimulus reflects a more heterogeneous response change including both boosting and suppression. (E) Mean (left) and variance (right) of the change in pyramidal responses to their preferred and non-preferred stimuli overlearning. Responses to the non-preferred stimulus decreased (p = 0.003, two-sided sign test, n = 776 cells), but responses to the preferred stimulus did not (p = 0.8, two-sided sign test; p = 0.025, one-sided Wilcoxon rank sum test on difference between preferred and non-preferred stimulus response change, n = 776 cells). The variance over cells of response changes was higher for the preferred than non-preferred stimulus (p = 0.035, shuffling test, n = 776 cells). Error bars show SEM and standard error in the variance (SEV). See also Figure S7.

In Khan et al. (2018), we found that response SNRs of both PYR and PV cells increased over learning and that these improvements were driven by an emergence of stimulus-specific PYR to PV interaction weights in the MVAR model. We therefore reasoned that a change in E-I connectivity that is specific to the learned stimuli might account for the observed realignment of slow dynamical modes. Thus, we considered a non-uniform ring network in which excitatory to inhibitory synaptic weights were strengthened locally among neurons tuned to a particular orientation (Figure 6D). We found that the resulting non-uniform inhibition induced changes in dynamical modes that were consistent with those observed over learning in the MVAR model: the slowest-decaying mode became better aligned with the input discriminant, whereas its time constant was unchanged (Figures 6E, 6F, S6E, and S7A). Interneurons exhibited substantially weakened coupling into the translation mode in the non-uniform network, as found for PV interneurons in the MVAR model (Figures S7B and S4C). When stimuli were presented at ±20° relative to the subnetwork center (reflecting the 40°stimulus separation in the experiment), information was enhanced via a greater separation of responses around the ring (Figures 7A and S7C). In simulations of the full nonlinear network response to feed-forward input, accumulation of stimulus information was accelerated by non-uniform inhibition but slowed by uniform sharpening (Figure 7B). Experimental data showed an accelerated rate of integration over learning consistent with the non-uniform connectivity change (Figures 7C and S2F). Thus, in both the analysis of local linearized modes and the evolution of the nonlinear network responses over time, non-uniform changes in E-I connectivity accounted for the learning-related changes in responses imaged from the V1 circuit.

The tuning curve changes induced by non-uniform connectivity (Figure 7A) generated further predictions that we subsequently tested on the experimental data. Responses of excitatory neurons to their non-preferred stimulus were consistently suppressed by non-uniform inhibition, whereas responses to their preferred stimulus showed a heterogeneous combination of boosting and suppression (Figure 7D). Changes over learning in imaged PYR cell responses showed a similar pattern (Figure 7E). Moreover, the average response SNR of both excitatory and inhibitory neurons increased in the model (Figures S7D and S7E), as previously reported for the imaged responses of PYR cells and PV-expressing interneurons (Khan et al., 2018; reproduced in Figure S7E). Despite these improvements in single-cell response SNR, neither E nor I populations increased their loading onto the linear discriminant, as found for PYR and PV neurons in the data (Figure S7B). Finally, non-uniform inhibition increased the slope of tuning curves flanking the E-I subnetwork, as observed in primate V1 following learning of a fine-scale orientation discrimination task (Figure S7F; Schoups et al., 2001). Importantly, although dynamical realignment through non-uniform inhibition required that feedforward input was more broadly tuned than network output (Figures 6B and 6E), feed-forward and recurrent input could nonetheless have very similar tuning widths as reported experimentally (Lien and Scanziani, 2013; see Figures S7G–S7L).

Taken together, these findings demonstrate that the learning-related changes in imaged network responses are consistent with the emergence of stimulus-specific excitatory to inhibitory synaptic connectivity within cortical circuits. These connectivity changes act to increase response information by aligning slowly decaying dynamical modes with the optimal discriminant of sensory input in order to selectively integrate relevant sensory information over time.

## Discussion

We have developed a general framework for modeling the integration and transmission of sensory information through recurrent networks and leveraged this framework to uncover the changes in recurrent processing that drive improvements in sensory representations over learning. Previous studies suggested that recurrent synapses selectively amplify or sharpen the tuning of feedforward input (Douglas et al., 1995; Ben-Yishai, 1995; Somers et al., 1995; Murphy and Miller, 2009; Liu et al., 2011; Li et al., 2013; Lien and Scanziani, 2013; Cossell et al., 2015); however, theoretical analyses concluded that sharpening reduces population response information (Seriès et al., 2004; Beck et al., 2011). Others proposed that recurrent synapses selectively suppress responses to remove redundancy between similarly tuned neurons (Olshausen and Field, 1996; Lochmann and Deneve, 2011; Znamenskiy et al., 2018; Chettih and Harvey, 2019); however, such mechanisms cannot explain the improvements in response information as animals learn to discriminate simple sensory features such as oriented grating stimuli (Poort et al., 2015; Khan et al., 2018). Instead, we show that recurrent cortical dynamics perform selective temporal integration of relevant sensory information and that learning modifies cortical dynamics in order to selectively integrate task-relevant sensory input.

While recurrent integration of sensory information has long been implicated in decision-making tasks (Shadlen and Newsome, 2001; Wong and Wang, 2006; Goldman et al., 2009a; Mante et al., 2013), our work makes three novel contributions. First, previous work has analyzed recordings of single neurons (or small populations) and has therefore turned to hand-crafted circuit models or task-trained recurrent neural networks to investigate possible dynamical mechanisms for the integration of sensory input (e.g., Wong and Wang, 2006; Mante et al., 2013). Instead, we fit a dynamical model directly to large-scale cortical population activity and analyzed how sensory input was integrated within this model, an approach that was made possible by the simultaneous nature of our recordings. Second, previous studies have not addressed how learning modifies recurrent integration to prioritize relevant sensory information. By fitting a dynamical systems model to population activity from the same neurons before and after learning, we identified the changes in dynamics that drive improvements in cortical representations for task-relevant stimuli with learning. Third, previous studies focused on decision-making tasks in which the distal stimulus was noisy or variable, requiring temporal integration even when neural processing is perfectly noiseless (Brunton et al., 2013). Here, we show that temporal integration occurs even for noiseless stimuli, where all information relevant to the decision is immediately available in the distal stimulus. This suggests a role for temporal integration in mitigating internal physiological noise that would otherwise degrade information propagation during sensory processing (Faisal et al., 2008).

We inferred cortical dynamics by fitting linear dynamical models to imaged population activity. Such an approach is prone to model mismatch, such that temporally coordinated external input may be erroneously attributed to local interactions among cells. Thus, although the MVAR model identified changes in dynamics over learning, it is possible that such dynamics are inherited by the local circuit or generated through a broader network of cortical and subcortical structures. Although our E-I circuit model (Figures 6 and 7) synthesizes and predicts numerous findings in our data, including the increase in PYR and PV selectivity for relevant stimuli, reorganization of PYR-PV but not PYR-PYR interactions, realignment but not slowing of dynamical modes, weakened coupling of PV but not PYR neurons into high SNR modes, and suppression of PYR responses to their non-preferred stimulus with learning, it is nonetheless possible that all of these properties are inherited by the V1 circuit via external input from a downstream integrator. Such hypotheses could be tested in future experiments by recording neuronal population activity in multiple brain regions simultaneously during sensorimotor decision-making tasks. Additional confounds in the MVAR analysis may arise through the convolution of neuronal responses by slow calcium dynamics and the temporal resolution of the data (~ 125 ms). However, although these may lead to an overestimate of the time constants of network dynamics, they cannot trivially explain the change in alignment of dynamical modes observed over learning. Although we observed an apparent decrease in non-normality over learning, measurements at higher temporal resolution are necessary to detect rapid forms of non-normal dynamics and their changes over learning (Murphy and Miller, 2009).

Responses of cells in primary visual cortex have been found to decay within a single neuronal time constant when thalamic input is removed (Reinhold et al., 2015). Can the long timescales of recurrent dynamics required for selective temporal integration be reconciled with these observations? One possibility is that the dynamical regime of cortex is dependent on tonic thalamic input or on thalamocortical loops. Alternatively, Reinhold and colleagues may have predominantly activated and measured rapidly decaying modes of dynamics which obscured the presence of weakly activated slowly decaying modes intermixed with the population response. Unless these slowly decaying modes of dynamics comprise a substantial fraction of the total response variance, their detection requires recording from neural populations, whereas Reinhold and colleagues recorded single neurons. Future studies could test these hypotheses by measuring and perturbing different patterns of population activity during sensory stimulation and quantifying the time constants of network responses.

Our theory explains a recent report that information-limiting noise correlations are higher when animals make correct decisions compared with incorrect ones (Valente et al., 2021). Because these correlations reduce the information about the stimulus available in the network response relative to an uncorrelated population and yet were associated with improved behavioral accuracy, these findings were considered to be paradoxical by Valente and colleagues. Instead, we show that these findings are an expected signature of optimal integration of sensory input through the recurrent circuit dynamics. In particular, we observe that information-limiting response correlations across neurons are maximized when networks integrate their sensory input optimally (compare Figures 2F with 2H and S1A, ellipses which are more elongated along the direction which separates the two means have higher information-limiting correlations; see also Figures S1B–S1D). Valente and colleagues also found that correlations between responses at different time points within a trial are higher when animals make correct decisions, which was considered paradoxical because such correlations limit the ability of downstream readers to decode the stimulus over the duration of a trial. We show that strong temporal correlations are an expected signature of optimal integration of sensory input through time by the circuit. Thus, we suggest that optimal sensory coding is best understood in terms of the transformation of sensory input signals by the neural circuit, a perspective which leads to fundamentally different experimental predictions for the optimal response statistics than those obtained using abstract neural encoding models (see also Series et al., 2004; Beck et al., 2011; Huang et al., 2022).

Several previous studies have investigated information transmission through recurrent networks (Seriès et al., 2004; Ganguli et al., 2008; Beck et al., 2011; Toyoizumi and Abbott, 2011; Dambre et al., 2012; Najafi et al., 2020; Huang et al., 2022). Although most studies (correctly) concluded that information in network output cannot exceed that contained in the input, such studies either (1) quantified information in time-integrated network responses (Seriès et al., 2004; Moreno-Bote et al., 2014), (2) modeled sensory input as being static within each trial, varying only from trial to trial (Najafi et al., 2020), or (3) analyzed network models which lack the capacity for dynamical integration (Beck et al., 2011). In our analysis, input noise was time varying, and recurrent dynamics could integrate input over the course of a trial, allowing the instantaneous response to carry more information than that of the instantaneous input. Although Toyoizumi and Abbott considered a similar scenario, their analysis was restricted to networks of randomly connected neurons with anti-symmetric, saturating transfer functions.

Our analysis provides a general framework for understanding evidence integration in neural circuits, such as path integration in grid cells, vestibular integration in head direction cells, and integration of motion in higher visual areas. While several of these systems have been studied mechanistically as attractor networks (Wong and Wang, 2006; Burak and Fiete, 2009) or statistically as drift-diffusion and population coding models (Ratcliff and McKoon, 2008; Averbeck et al., 2006), our approach provides a unifying formalism which links statistical properties of evidence integration and population coding to the dynamical properties of the underlying recurrent network. Although we have focused on changes in network dynamics over learning, the mechanism of dynamical alignment may also provide a substrate for contextual or attentional modulation of sensory processing (Gilbert and Li, 2013). Specifically, top-down input may modulate the dynamics of recipient neural populations, transiently aligning dynamical modes of the local circuit with relevant features of bottom-up sensory input according to task context. Such a mechanism could allow for flexible routing and gating of information between brain areas through the dynamical formation and coordination of “communication subspaces” (Semedo et al., 2019; Kohn et al., 2020; Javadzadeh and Hofer, 2022), configured through selective alignment of local modes across anatomically distributed circuits.

## Star★Methods

Detailed methods are provided in the online version of this paper and include the following:

- KEY RESOURCES TABLE
- RESOURCE AVAILABILITY
  - Lead contact
  - Materials availability
  - Data and code availability
- EXPERIMENTAL MODEL AND SUBJECT DETAILS
- METHOD DETAILS
  - Analysis of optimal stimulus discrimination function (Figure 1)
  - Analysis of linear Fisher Information in recurrent networks (Figures 2 and S1)
  - Multivariate autoregressive system model and analysis of neural data (Figures 3, 5, and S2–S4)
  - Network models (Figures 6, 7, and S5–S7)

### Key Resources Table

**Table:** 

REAGENT or RESOURCE	SOURCE	IDENTIFIER
Antibodies		
Goat anti-parvalbumin	Swant	PVG-213; RRID: AB_2650496
Mouse anti-parvalbumin	Swant	PV-235; RRID: AB_10000343
Rabbit anti-Vasoactive intestinal peptide	ImmunoStar	Cat# 20077; RRID: AB_572270
Rat anti-somatostatin	Millipore	MAB354; RRID: AB_2255365
DyLight 405-AffiniPure Donkey Anti-Mouse	Jackson ImmunoResearch	Cat# 715-475-150; RRID: AB_2340839
Rhodamine Red-X-AffiniPure Donkey Anti-Rabbit	Jackson ImmunoResearch	Cat# 711-295-152; RRID: AB_2340613
Alexa Fluor 647-AffiniPure Donkey Anti-Rat	Jackson ImmunoResearch	Cat# 712-605-153; RRID: AB_2340694
Alexa Fluor 594-AffiniPure Donkey Anti-Mouse	Jackson ImmunoResearch	Cat# 715-585-151; RRID: AB_2340855
Alexa Fluor 647-AffiniPure Donkey Anti-Rabbit	Jackson ImmunoResearch	Cat# 711-605-152; RRID: AB_2492288
DyLight 405-AffiniPure Donkey Anti-Rat	Jackson ImmunoResearch	Cat# 712-475-153; RRID: AB_2340681
DyLight 405-AffiniPure Donkey Anti-Goat	Jackson ImmunoResearch	Cat# 705-475-147; RRID: AB_2340427
Bacterial and virus strains		
AAV2.1-syn-GCaMP6f-WPRE	Addgene	Cat#100837
Experimental models: Organisms/strains		
Mouse: C57BL/6	Biozentrum animal facility	N/A
Mouse: Rosa-CAG-LSL-tdTomato (JAX: 007914) crossed with PV-Cre (JAX: 008069)	Jackson Laboratory	JAX: 007914; RRID IMSR_JAX:007914 JAX: 008069; RRID IMSR_JAX:008069
Mouse: Rosa-CAG-LSL-tdTomato (JAX: 007914) crossed with VIP-Cre (JAX: 010908)	Jackson Laboratory	JAX: 007914; RRID IMSR_JAX:007914 JAX: 010908; RRID IMSR_JAX:010908
Software and algorithms		
Matlab	Mathworks	https://ww2.mathworks.cn/products/matlab.html; RRID: SCR_001622
Network model	Custom code	https://doi.org/10.5281/zenodo.7109995

### Resource Availability

#### Lead contact

Further information and requests for resources and reagents should be directed to and will be fulfilled by the lead contact and corresponding authors Angus Chadwick (angus.chadwick@ed.ac.uk) and Maneesh Sahani (maneesh@gatsby.ucl.ac.uk).

#### Materials availability

This study did not generate new unique reagents.

### Experimental Model And Subject Details

No new experimental data were collected for the purposes of this study. The acquisition and pre-processing of data used in this study are described in detail in Khan et al. (2018).

### Method Details

#### Analysis of optimal stimulus discrimination function (Figure 1)

In the Methods S1 File we analyze the problem of stimulus discrimination from a signal processing (or ideal observer) perspective. We consider a network receiving noisy but stimulus-tuned input and tasked with reporting stimulus identity in its output. Under the assumption that the input time series for a given stimulus follows a multivariate normal distribution with temporally uncorrelated, stimulus-independent noise, we show that the statistically optimal method for discriminating two stimuli is to perform a linear projection and temporal filtering of the input time series. We derive the optimal projection weights and filter, and the signal to noise ratio (SNR) obtained using an arbitrary projection and filter. While our conclusions rely on the specific assumptions mentioned above, these results provide intuition that could be extended to more complex scenarios. For example: if input noise is stimulus-dependent or non-Gaussian the optimal decoder typically becomes nonlinear (Shamir and Sompolinsky, 2004; Yang et al., 2021), if stimulus-independent temporal correlations are present in the input the benefits of temporal integration are typically reduced (see Methods S1 File), but stimulus-dependent temporal correlations could be extracted by a nonlinear filter to enhance discrimination performance. The key insight of this signal processing analysis is therefore that stimuli can be optimally discriminated based on a spatiotemporal filtering of single-trial sensory input, and that the form of the optimal filter depends on the statistics of the input signals.

In Figure 1 we sought to illustrate these observations in a minimal toy example consisting of a reduced two-dimensional system describing the feedforward input to two neurons under each of two stimuli. The dimensionality and statistics of the input were chosen primarily to optimize visualization and conceptual insight - our analysis allows for arbitrary numbers of neurons receiving input with arbitrary stimulus-tuning and noise covariance. For each stimulus *s_i_* (*i* = 1,2) and at each timestep *t*, feedforward inputs **u**(*s_i_*, *t*) ~ *N*(**g**(*s_i_*), *∑*_η_) were sampled independently from a multivariate normal distribution with stimulus-dependent mean **g**(*s*_1_) = [1,2], **g**(*s*_2_) = [2,1] and stimulus-independent covariance *∑*_η_ = [2, 1; 1, 2] (here and throughout, we will use the shorthand notation that matrix elements separated by commas are on the same row, while elements separated by a semicolon are on separate rows, e.g. [*x*, *y*] = [*x*;*y*]^*T*^). These time series were projected onto the linear discriminant ${\text{w}}_{LD}=\sum_{\text{η}}^{-1}(\text{g}(s_{2})-\text{g}(s_{1}))$ to obtain $d_{{\text{w}}_{LD}}(s,t)={\text{w}}_{LD}^{T}\text{u}(s,t)$ before being summed cumulatively over time to obtain $D_{{\text{w}}_{LD}}(s,t)=\sum_{t'=1}^{t}d_{{\text{w}}_{LD}}(s,t)$. The signal (difference in mean), noise (standard deviation), and signal to noise ratio of the projection of instantaneous input onto a vector **w**, *d*_**w**_(*s*, *t*) = **w**^*T*^**u**(*s*, *t*), were plotted using analytical expressions *Δμ*_input_(**w**) ≡ 〈*d*_**w**_(*s*_2_, *t*) - *d*_w_(*s*_1_, *t*)〉 = **w**^*T*^(**g**(*s*_2_) - **g**(*s*_1_)), $\sigma_{\text{input}\;}(w)\equiv \sqrt{0.5\sum_{i=1,}\langle {\left(d_{w}(s_{i},t)-\langle d_{w}(s_{i},t)\rangle \right)}^{2}\rangle }=\sqrt{w^{T}{\text{Σ}}_{\eta }w},{\text{SNR}}_{\text{input}\;}(w)=\Delta \mu_{\text{input}\;}(w)/\sigma_{\text{input}\;}(w)$. Following temporal integration, the corresponding quantities $D_{\text{w}}(s,t)=\sum_{t'=1}^{t}d_{\text{w}}(s,t)$ were plotted as $\Delta \mu_{\text{input}\;}(w,t)\equiv \langle D_{w}(s_{2},t)-D_{w}(s_{1},t)\rangle =\Delta \mu_{\text{input}\;}(w)t,\sigma_{\text{input}\;}(w,t)\equiv \sqrt{0.5\sum_{i=1,2}\langle (D_{\text{w}}(s_{i},t)-{\langle (D_{\text{w}}(s_{i},t)\rangle )}^{2}\rangle }=\sigma_{\text{input}\;}(w)\sqrt{t}{\text{,and}\;\;\text{SNR}}_{\text{input}\;}(w,t)\equiv \Delta \mu_{\text{input}\;}(w,t)/\sigma_{\text{input}\;}(w,t)={\text{SNR}}_{\text{input}\;}(w,t)\sqrt{t}$. Iso-probability contours at one standard deviation under each stimulus were plotted as $\text{g}\left(s_{i}\right)+\sqrt{\Sigma_{\text{η}}}[\cos\theta ;\sin\theta ]\;\;\text{for}\;\theta \in [0,2\pi )$.

#### Analysis of linear Fisher Information in recurrent networks (Figures 2 and S1)

Linear Fisher Information quantifies the accuracy of a locally optimal linear estimator of a stimulus from network responses (Series et al., 2004; Beck et al., 2011). When network responses follow a multivariate normal distribution, the linear Fisher Information takes the form of a (squared) signal to noise ratio. We derived analytical expressions for the linear Fisher Information of the instantaneous output of a recurrent network as a function of its input statistics and dynamics, and for the SNR of network output projected onto any one of its dynamical modes (see Methods S1 File). Our results hold for networks with arbitrary numbers of neurons with arbitrary non-linearities and synaptic connectivity, receiving sensory input with arbitrary stimulus-tuning and noise covariance. Our strongest modeling assumptions were the linearization of dynamics about a fixed point and the analysis of stationary state response statistics. We note that under the assumptions made for the sensory input described above, these linearized networks can achieve the optimal solution to stimulus decoding. However, in the more general case of non-Gaussian, stimulus-dependent and temporally correlated input noise, integration through nonlinear network dynamics may be required for optimal stimulus discrimination. Thus, our analysis may be considered as the simplest scenario, but the insight obtained about how information is integrated through both space and time to optimize neural coding should generalize to more complex situations.

##### Signal to noise ratio along dynamical modes (Figure 2)

To illustrate the relationship between network dynamics and population coding, we constructed a minimal toy model comprising a two-dimensional linear dynamical system *d***r**/*dt* = *A***r** + **u**(*s_i_*, *t*) corresponding to the linearized dynamics of the firing rates **r** = [*r*_1_; *r*_2_] of two reciprocally connected neurons. The weight matrix *A* was constructed by defining two dynamical modes with activation patterns **m**_*i*_ and corresponding time constants *δ_i_*. We consider a system without oscillations, i.e. one in which the eigenvalues *λ_i_* of *A* are real. In that case, *τ_i_* = - 1/*λ_i_* and the unique weight matrix which generates these dynamical modes is given by *A* = *M*^-1^ *ΛM*, where $M=[{\text{m}}_{1}^{T};{\text{m}}_{2}^{T}]$ and *Λ* = [*λ*_1_, 0; 0, *λ*_2_] (note that we define the mode activation patterns **m**_*i*_ to be the *left eigenvectors* of *A*, see Methods S1 File for details). We constructed **m***?* as unit length vectors with a given angle relative to the input linear discriminant using the equation **m**_*i*_ = *R*(*θ_i_*)**w**_*LD*_/‖**w**_*LD*_‖, where *R*(*θ_i_*) = [cos(*θ_i_*), − sin(*θ_i_*); sin(*θ_i_*), cos(*θ_i_*)] is a rotation matrix. **w**_*LD*_ was defined as the linear discriminant of two stimulus inputs with **g**(*s*_1_) = [6; 6], **g**(*s*_2_) = [5; 7], *Σ*_η_ = [20, 10; 10, 20] (these values, along with the modes and time constants, were chosen to primarily to optimize visualization). We constructed networks with one mode aligned to input linear discriminant and the other orthogonal to the first by setting *θ*_1_ = 0.02*π*, *θ*_2_ = *θ*_1_ + 3*π*/2. Forthe network with slowly-decaying mode aligned to the linear discriminant we set *δ*_1_ = 10, *τ*_2_ = 2 and for the network with rapidly-decaying mode aligned to input linear discriminant we set *τ*_1_ = 2, *τ*_2_ = 10 (in arbitrary units of time).

As Figures 2A-2C were designed to illustrate the dynamical modes of the network ratherthan the stimulus input, we set the input to **u** = (**g**(*s*_1_) + **g**(*s*_2_))/2(or **u** = [0; 0] before input onset). Network responses **r** were computed using the solution to the linear dynamics **r**(*t*) = exp(*At*)(**r**(0) - **r**_∞_) + **r**_∞_ where **r**(0) = [0; 0], **r**_∞_ = – *A*^–1^**u** and exp is the matrix exponential function. The perturbation was modeled by setting **r**(*t*_pert_) = **r**_∞_ + [0; 10] and computing all future time points as **r**(*t*) = exp(*A*(*t* – *t*_pert_))(**r**(*t*_pert_) – **r**_∞_) + **r**_∞_

For Figures 2D-2J, network responses to the two stimulus input time series were simulated using the Euler method with *dt* = 0.01, i.e. **r**(*t* + *dt*) = **r**(*t*) + (*A***r**(*t*) + **g**(*s_i_*) + **η**(*t*))*dt* where η(*t*) ~ *N*(0, *Σ*_η_). For visualization purposes, trajectories were smoothed before plotting for Figures 2E and 2G using a moving average box filter containing 100 time samples.

Input and output iso-probability ellipses were generated as in Figure 1, using the relevant mean and covariance matrix in each condition. Response means were computed using the analytical solution for a linear system at steady state, **r**_∞_ (*s*) = – *A*^–1^**g**(*s*), and response covariance matrices (Figures 2F and 2H) were computed as the solution to the Lyapunov equation *AΣ* + *ΣA^T^* + *Σ*_η_ = 0 using the Matlab function *lyap*.

The signal, noise, and signal to noise ratio ofstationary state responses projected along each mode $d_{{\text{m}}_{i}}\left(s,t\right)={\text{m}}_{i}^{T}\text{r}\left(s,t\right)$ were computed using the equations $\Delta \mu_{\text{output}\;}\left(m_{i}\right)\equiv \langle d_{m_{i}}(s_{2},t)-d_{m_{i}}(s_{1},t)\rangle =\Delta \mu_{\text{input}\;}\left(m_{i}\right)\tau_{i},\sigma_{\text{output}\;}\left(m_{i}\right)\equiv \langle \sqrt{0.5\sum_{k=1,2}{\left(d_{m_{i}}(s_{k},t)\;-\;\langle d_{m_{i}}(s_{k},t)\rangle \right)}^{2}}\rangle =\sigma_{\text{input}}({\text{m}}_{i})\sqrt{\tau_{i}/2}$, and SNR_output_(**m**_*i*_) = SNR_input_(**m**_*i*_) $\sqrt{2\tau_{i}}$ respectively, where *Δμ*_input_, *σ*_input_, SNR_input_ are as described for Figure 1 (see Methods S1 File for a derivation).

##### Non-normal dynamics (Figure S1)

We derived expressions relating linear Fisher Information to the dynamics of an arbitrary normal or non-normal network (subject to the same approximations described above). These expressions had a simple and interpretable form in three special cases: two-dimensional networks, normal networks, and non-normal networks with strong functionally-feedforward dynamics. Related findings have been presented previously (Ganguli et al., 2008; Goldman, 2009b).

To illustrate our analytical findings for the two-dimensional case, we constructed networks with modes **m**_1_ = [cos*θ*_1_; sin*θ*_1_], **m**_2_ = [cos*θ*_2_; sin*θ*_2_]. Figure S1A was constructed using the same procedure as for Figure 2, but this time with *τ*_1_ = 10, *τ*_2_ = 5. For Figure S1B we chose input with isotropic covariance *Σ*_η_ = *I*_2_ (where *I_N_* is the N x N identity matrix) and *Δ***g** = **g**(*s*_2_) - **g**(*s*_1_) = [1; 0]. These inputs were chosen in order to demonstrate the influence of non-normality as clearly as possible. We set *τ*_1_ = 10, *τ*_2_ = 1,5,7.5,9 and varied *θ*_1_, *θ*_2_ from – *π*/2 to *π*/2 for each value. For each network (defined by the parameters *θ*_1_, *θ*_2_, *τ*_1_, *τ*_2_ using the procedure described for Figure 2), the Fisher Information of the stationary state network response $I_{F}=\Delta r\cdot \Sigma^{-1}\Delta r$ was computed by substituting the long-run solution for the mean *Δ***r** = – *A*^–1^ *Δ***g** and the numerical solution to the Lyapunov equation for *Σ* (described above). We normalized this linear Fisher Information by the maximum achievable SNR in any normal network with the same time constants by defining $I_{F,\text{norm}}=I_{F}/(\Delta {\text{g}}^{T}\Sigma_{\text{η}}^{-1}\Delta \text{g2}\tau_{1})$. For each network, we computed the information-limiting correlations as *ρ_ILC_* = *Δ***r**^*T*^ *ΣΔ***r**/(*Δ***r**^*T*^ *Δ***r**Trace(*Σ*)). For each choice of *τ*_2_, we computed the Pearson correlation between the Fisher information and the information-limiting correlations corr(𝓘_*F*, *ρ_ILC_*_), where the correlation was computed over a set of networks spanning the range of *θ*_1_, *θ*_2_ ∈ [- *π*/2, *π*/2). We computed this correlation for various settings of *Σ*_η_ = [**v**_1_, **v**_2_][*λ*_1_, 0; 0, *λ*_2_][**v**_1_, **v**_2_]^*T*^, by varying the angle of its principal eigenvector **v**_1_ from *Δ***g** and the ratio of its two eigenvalues *λ*_2_/*λ*_1_ with *λ*_1_ = 1 and *λ*_2_ ∈ [0, 1].

To illustrate functionally-feedforward networks (Goldman, 2009b), we constructed networks with NxN weight matrix *A_ij_* = (− 1 /*τ*)*δ_ij_* + *ωδ*_*i*,*j* + 1_, while varying the weight *ω* and number of neurons *N* for fixed single-cell time constants *τ* = 10 (where *δ_ij_* is the Kronecker delta symbol). We set *Δg_i_* = *δ*_*i*1_ and *Σ*_η_ = *I_N_*. We derived analytical expressions in the *ω* → ∞ limit for the linear Fisher Information of network output at stationary state, the temporal filter the network applies to its input, and the optimal linear readout of network responses. We numerically extended our results to the finite *ω* case by computing the response signal, response covariance, and linear Fisher Information in the same way as for the two-dimensional networks. To understand how the finite *ω* and large *ω* networks differ and where the large *ω* approximation breaks down, we also computed the SNR of the finite *ω* network responses projected onto the large *ω* optimal readout. Full derivations can be found in the Methods S1 File.

#### Multivariate autoregressive system model and analysis of neural data (Figures 3, 5, and S2–S4)

Details of the experiment, data preprocessing, calculation of behavioral d-prime (Figure 3B), and fitting and validation of MVAR model on this dataset have been described in detail in previous publications (Khan et al., 2018; see also Poort et al., 2015, 2022). The MVAR model used in this study, and the data the model were fit to, were identical to those of Khan et al. (2018). In particular, in all studies the data comprised multiple cell types (PYR, PV, SOM and VIP) and the model was fit to all simultaneously imaged cells using a least-squares method that was blind to cell type. Any cell type-specific analyses were performed post hoc based on the fitted model. For model performance on held out data, see Figure S10 of Khan et al. (2018). Here, we summarize the MVAR model and provide details of novel MVAR analyses used in the present study.

The imaged *ΔF/F* signals for each cell were divided into trials of duration -1 to 1 s relative to the onset of a visual stimulus. Here and below, all sums over time samples are restricted to the *N_t_* = 9 time samples contained in the post-stimulus window of 0 to 1 s (although the model was fit to the full window of -1 to 1 s containing 17 time samples). We collect the population activity of *N* simultaneously imaged neurons at imaging frame *t* on trial *i* into an *N* -dimensional vector denoted ${\text{r}}_{t}^{\left(i\right)}$. We define the following quantities which we will make use of below. The trial-averaged activity conditioned on stimulus *s* and time relative to stimulus onset *t* is ${\bar{r}}_{t}^{\left(s\right)}=\left(1/N_{\text{Trials }}(s)\right)\sum_{i\;\in \;\text{Trials}(s)}r_{t}^{\left(i\right)}$, where *N*_Trials_(*s*) is the number trials of stimulus *s*. The grand average over both time samples and trials conditioned on the stimulus *s* is ${\bar{r}}^{\left(s\right)}=\left(1/N_{t}\right)\sum_{t=1}^{N_{t}}{\bar{r}}_{t}^{\left(s\right)}$. The pooled covariance over vertical (*V*) andangled (*A*) stimuli is $\sum ={\left(N_{t}(N_{\text{Trials}}(V)+N_{\text{Trials}}(A))\right)}^{-1}\sum_{s=VA}\sum_{i\in \;\text{Trials}(s)}\sum_{t=1}^{N_{i}}({\text{r}}_{t}^{\left(i\right)}-{\bar{\text{r}}}_{t}^{\left(s\right)}){\left({\text{r}}_{t}^{\left(i\right)}-{\bar{\text{r}}}_{t}^{\left(s\right)}\right)}^{T}$. The linear discriminant of population responses to the vertical and angled stimuli was defined as $w_{LD}^{\text{output }}=\sum^{-1}({\bar{\text{r}}}^{V}-{\bar{\text{r}}}^{A})$.

##### Description of Model

To infer linear dynamics and stimulus input of the imaged circuit, we fit a multivariate autoregressive linear dynamical system model to the imaged responses. In the MVAR model, the imaged activity is modeled as:

(Equation 1) $${\text{r}}_{t}^{\left(i\right)}=(A+I_{N}){\text{r}}_{t-1}^{\left(i\right)}+{\text{u}}_{t}^{\left(s\right)}+\text{ξ}v_{t}^{\left(i\right)}+{\text{e}}_{t}^{\left(i\right)}$$

where *A* is an *N*×*N* matrix of interaction weights, ${\text{u}}_{t}^{\left(s\right)}$ is a vector of *N* stimulus-related inputs, ξ is a vector of *N* running speed co-efficients, $v_{t}^{\left(i\right)}$ is the running speed of the animal and ${\text{e}}_{t}^{\left(i\right)}$ is a vector of residuals.

The MVAR model was fit to each dataset by minimizing the sum of squared residuals across all neurons and trials of the vertical, angled, and gray corridor stimuli before or after learning (-1 to 1 s about the onset of the corridor, which appeared suddenly). Analytical expressions for the model parameters obtained under this least-squares fit offer insight into their interpretation (Equations 2, 3, and 4 in Khan et al. [2018]). In particular, the interaction weights depend only on the stimulus-independent covariance of the data (both the instantaneous covariance *Σ* and the covariance between consecutive imaging frames). Given these interaction weights, the stimulus-related input depends only on the stimulus-conditioned trial-averaged responses ${\bar{\text{r}}}_{t}^{\left(s\right)}$. Thus, the MVAR model uses the imaged noise covariance of the data (both within and across consecutive time samples) in order to infer interactions between cells and ascribes any remaining stimulus-dependent variation in trial-averaged responses to sensory input. The residuals have zero mean under each condition, i.e. $\sum_{i\;\in \;\text{Trials}(s)}{\text{e}}_{t}^{\left(s\right)}=0$ for any *t* and *s* (Equation 4 in Khan et al. [2018]).

In the main version of the model used in both this study and Khan et al. (2018), ξ was constrained to have the same value pre- and post-learning. In this model, changes in running behavior with learning could generate changes in response dynamics via the term $\text{ξ}v_{t}^{\left(i\right)}$ with fixed ξ and varying $v_{t}^{\left(i\right)}$. We also considered a second variant of the model with an additional lick-dependent input $\text{ζ}l_{t}^{\left(i\right)}$ added to the right hand side of Equation 1, where $l_{t}^{\left(i\right)}=1$ if the mouse licked at time *t* on trial *i* and $l_{t}^{\left(i\right)}=0$ otherwise and ζ was a vector of *N* lick coefficients that determined the influence of licking on neural activity. This model was used to determine whether behavioral changes with learning could offer an alternative explanation for the changes in responses. To allow the model maximum flexibility to capture neural responses via behavioral variables, we allowed the running and licking coefficients ξ and ζ to change with learning in this model. This allowed for the contribution of running and licking to vary over learning not only due to changes in behavior ($v_{t}^{\left(i\right)}$ and $l_{t}^{\left(i\right)}$) but also through changes in the relationship between behavior and neural activity (ξ and ζ). The results of this analysis are shown in Figures S3H–S3L.

##### Visualization of MVAR input and output along discriminant axis

Having fit the MVAR model to the experimental data, we sought to visualize how the imaged responses were generated through recurrent integration of stimulus-related input within the inferred dynamical system. To do so, we projected the sensory input, recurrent input, and MVAR output onto the linear discriminant in order to see how stimulus-discriminability evolved overtime. Single-trial sensory input was defined as ${\text{u}}_{t}^{\left(s\right)}+{\text{e}}_{t}^{\left(i\right)}$ (i.e. residuals were assigned as input noise), recurrent input as $(A+I_{N}){\text{r}}_{t-1}^{\left(s\right)}$, and MVAR output as ${\text{r}}_{t}^{\left(i\right)}$. The linear discriminant vectors were ${\text{w}}_{LD}^{\text{input}}=\sum_{\text{e}}^{-1}({\text{u}}^{V}-{\text{u}}^{A})$ and $w_{LD}^{\text{output }}=\sum^{-1}({\bar{\text{r}}}^{V}-{\bar{\text{r}}}^{A})$, where ${\text{u}}^{\left(s\right)}={\left(N_{\text{Trials}}(s)N_{t}\right)}^{-1}\sum_{t,j\;\in \;\text{Trials}(s)}({\text{u}}_{t}^{\left(s\right)}+{\text{e}}_{t}^{\left(i\right)})=(1/N_{t})\sum_{t}{\text{u}}_{t}^{\left(s\right)}$ and ${\sum_{e}\left(\left(N_{\text{Trials}}(A)+N_{\text{Trials}}(V)\right)N_{t}\right)}^{-1}\sum_{s=A,Vt,i\in }\sum_{\text{Trials(s)}}{\text{e}}_{t}^{\left(i\right)}{\text{e}}_{t}^{(i)T}$. The sensory input was projected onto ${\text{w}}_{LD}^{\text{input}}$, while both recurrent input and imaged responses were projected onto ${\text{w}}_{LD}^{\text{output}}$. We plotted the mean and standard deviation over trials of these projected activity patterns for a representative mouse in the post-learning condition.

For the more flexible MVAR model containing a lick-dependent term and allowing licking and running coefficients to change with learning, we computed the projection of each term along the input and output learning discriminants for each mouse before and after learning. We averaged these projections across trials for each mouse and then averaged across animals to obtain the results shown in Figure S3H.

##### Quantification of MVAR input and output information

The stimulus information (or linear discriminability) of single-imaging frame population responses was quantified as $I_{\text{out}}={\left({\bar{\text{r}}}^{V}-{\bar{\text{r}}}^{A}\right)}^{T}\sum^{-1}({\bar{\text{r}}}^{V}-{\bar{\text{r}}}^{A})$. The stimulus information of inferred input was quantified as $I_{\text{in}}={\left({\text{u}}^{V}-{\text{u}}^{A}\right)}^{T}\sum_{\text{e}}^{-1}({\text{u}}^{V}-{\text{u}}^{A})$. These metrics were computed separately for the pre-and post-learning data for each mouse. The gain in output to input information was defined as 100 × ((*l*_out_ /*l*_in_) − 1).

##### Quantification of temporal integration of relevant and irrelevant input

To test how temporal integration of relevant and irrelevant input changed over learning in the MVAR model, we analyzed the impulse-response of the MVAR to two different input perturbations. The impulse-response to a perturbation **p** was modelled by setting the MVAR to an initial state **r**_0_ = **p** and forward-simulating the system over multiple time steps with no other input, i.e. **u**_t_, **e**_t_, *v_t_* = 0. This gave the response **r**_*t*_ = (*A* + *I_N_*)^*t*^**p**. Simulated responses **r**_*t*_ were then projected onto a vector **w**. For the relevant input, we chose **p** to be the MVAR input linear discriminant $\text{p}\propto \sum_{\text{e}}^{-1}({\text{u}}^{V}-{\text{u}}^{A})$ and **w** to be the linear discriminant of the imaged population responses $\text{w}\propto \sum^{-1}({\bar{\text{r}}}^{V}-{\bar{\text{r}}}^{A})$ (**w** and **p** were computed separately pre- and post-learning). With this choice (i.e., by choosing not to enforce **w** = **p**), we allow for the possibility that temporal integration occurs through either normal or non-normal dynamics (Figure S1). For the task-irrelevant input we chose $\text{p}\propto \sum_{\text{e}}^{-1}({\text{u}}^{V}+{\text{u}}^{A})$ and $\text{w}\propto \sum^{-1}({\bar{\text{r}}}^{V}+{\bar{\text{r}}}^{A})$. Time constants of network responses were defined as $\tau =(T_{s}/2){\left[\sum_{t=0}^{\infty }{\text{r}}_{t}\cdot {\text{w}}_{\text{out}}\right]}^{2}/{\left[\sum_{t=0}^{\infty }{\text{r}}_{t}\cdot {\text{w}}_{\text{out}}\right]}^{2}$, which was adapted from the analytically-derived temporal integration factor *I_T_*(*f*) in the Methods S1 File (see section titled signal processing analysis).

As a more comprehensive control analysis, we generated a distribution of input vectors sampled as random combinations of the vertical and angled stimulus inputs to each neuron, $p_{i}\propto {\sum_{j}\left(\sum_{\text{e}}^{-1}\right)}_{ij}(\eta_{j}^{V}u_{j}^{V}-\eta_{j}^{A}u_{j}^{A})$ and $w_{i}\propto {\sum_{j}\left(\sum^{-1}\right)}_{ij}(\eta_{j}^{V}r_{j}^{V}-\eta_{j}^{A}r_{j}^{A})$ with $\eta_{i}^{X}\sim N(0,1)$ a set of independent standard normal random variables. We generated 10,000 such random input vectorsand computed the time constant *τ* before and after learning for each one. The results are shown in Figure S3A. Note that the linear discriminant input and the task-irrelevant input described in the previous paragraph are both contained in this distribution of input vectors.

##### Constrained model fits

To test whether the learning-related changes in temporal integration in the MVAR model require changes in interaction weights or stimulus input, we refit the MVAR with either *A* or **u** constrained be the same both pre- and post-learning. We then repeated the analyses for Figure 3 on the constrained MVAR model fits. Details of the constrained model fitting procedure are provided in Khan et al. (2018).

##### Input and output SNR along MVAR modes

To compute the SNR of network input and output projected onto each mode, we used analytically derived expressions which relate these SNRs to the eigenvectors and eigenvalues of *A*. Eigenvectors (right ${\text{v}}_{i}^{R}$ and left ${\text{v}}_{i}^{L}\equiv {\text{m}}_{i}$) and eigenvalues *λ_i_* of the pre- and post-learning MVAR interaction weight matrices *A* were numerically computed using the Matlab function *eig*. The SNR of stimulus input projected along each mode was then given by the equation ${\text{SNR}}_{\text{input}}({\text{m}}_{i})\equiv \Delta \mu_{\text{input}}({\text{m}}_{i})/\sigma_{\text{input}}({\text{m}}_{i})=\left|{\text{m}}_{i}\cdot ({\text{u}}^{V}-{\text{u}}^{A})\right|/\sqrt{{\text{m}}_{i}\cdot \sum_{\text{e}}{\text{m}}_{i}}$. The normalized input SNR was ${\text{SNR}}_{\text{norm}}({\text{m}}_{i})={\text{SNR}}_{\text{input}}({\text{m}}_{i})/{\text{SNR}}_{\text{input}}({\text{w}}_{LD,\text{input}})$, where ${\text{w}}_{LD,\text{input}}=\sum_{\text{e}}^{-1}({\text{u}}^{V}-{\text{u}}^{A})$ is the input linear discriminant and ${\text{SNR}}_{\text{input}}{\text{(w}}_{LD,\text{input}})=\sqrt{{\left({\text{u}}^{V}-{\text{u}}^{A}\right)}^{T}\sum_{\text{e}}^{-1}({\text{u}}^{V}-{\text{u}}^{A})}$ is the SNR of input projected along the linear discriminant. We computed the time constant of each mode using the equation *τ_i_* = - *T_s_*/log(*λ_i_* + 1) which converts from a discrete-time dynamical system of sampling period *T_s_* to a time constant in an equivalent continuous-time dynamical system. We restricted our analysis of individual modes to those with real eigenvalues *λ_i_* + 1 > 0 (which ensures that *τ_i_*, are real, so that the mode is not oscillatory).

We pooled modes across animals separately in the pre- and post-learning conditions (note that individual modes are not matched pre- vs post-learning). Both pre- and post-learning, we performed averages over time constants conditioned on normalized input SNRs and over normalized input SNRs conditioned on time constants. These conditional averages were obtained using a moving average analysis. To obtain an average normalized input SNR conditioned on time constant, we used a box filter of width 100 ms with center increasing from 100 ms to 1400 ms in increments of 25 ms. For each increment, we computed the mean normalized input SNR of all modes within that window. Similarly, we used a box filter of width 0.025 increasing from 0.025 to 0.25 to compute average time constant conditioned on normalized input SNR. As an additional analysis, we computed a two-dimensional histogram describing the number of modes *n*(*τ*, SNR_norm_) with time constant *τ* and normalized input SNR SNR_norm_ by applying a moving two-dimensional Gaussian filter over the set of modes using the equation $n\text{(}\tau \text{,}\;{\text{SNR}}_{\text{norm}}\text{)}=\sum_{i=1}^{N_{\text{modes}}}\text{exp}-[{\left(\tau_{i}-\tau \right)}^{2}/(2\sigma_{\tau }^{2})+{\left({\text{SNR}}_{\text{norm}}({\text{m}}_{i})-\;{\text{SNR}}_{\text{norm}}\right)}^{2}/2\sigma_{\text{SNR}}^{2}]$. We set *σ_τ_* = 100 ms and *σ*_SNR_ = 0.025. We computed the change over learning *Δn* = *n*_post_ – *n*_pre_ and normalized this quantity by its standard deviation across shuffled data (see below) to obtain *Δn*/*σ*(*Δn*_shuff_), a measure of the change relative to chance level, which is plotted in Figure 5F.

To determine whether learning-related changes in time constants or normalized input SNRs exceeded chance level, we performed a bootstrapping procedure based on shuffling of trials. For each mouse, we pooled pre- and post-learning trials and randomly resampled (without replacement) two sets of trials of equal number to the pre- and post-learning datasets. These shuffled datasets constituted the null hypothesis that no changes occurred overlearning. Wethen refit the MVAR model to each of these shuffled data-sets and repeated the above analyses to obtain the time constants and normalized input SNRs under the null hypothesis. In this way, we generated a null distribution for each statistic (moving average of change in time constant, moving average of change in normalized input SNR, and *Δn*). We then formed 95 % confidence intervals for each statistic based on their respective null distributions. Our null distributions consisted of 1000 such shuffles.

To test whether our results were biased by individual mice, we also performed within-animal averages of the time constants and normalized input SNRs pre- and post-learning (Figures S3D and S3E). For this analysis, individual mice were considered as the statistical unit when performing significance testing.

##### MVAR non-normal dynamics

The non-normality of dynamics was quantified using Henrici’s departure from normality (Henrici, 1962): $H=\sqrt{{\left\|A\right\|}_{F}^{2}-\sum_{i=1}^{N}{\left|\lambda_{i}\right|}^{2}/{\left\|A\right\|}_{F}}$, where ‖*A*‖_*F*_ is the Frobenius norm. This measure was computed separately on the interaction weight matrix for pre- and post-learning data for each animal (Figure S3F). We also computed the angle between the input linear discriminant $\text{p}=\sum_{\text{e}}^{-1}({\text{u}}^{V}-{\text{u}}^{A})$ and output linear discriminant $\text{w}=\sum^{-1}({\bar{\text{r}}}^{V}-{\bar{\text{r}}}^{A})$ as a measure of functionally-feedforward integration of task-relevant sensory input (Figure S3G).

##### Analysis of false alarm trials

Analysis of behavioral errors was restricted to the post-learning data. False alarm trials and hit trials were defined as trials in which the mouse licked within 4 seconds of the angled and vertical grating stimulus onset respectively. Using a sliding time window of width 2 time bins (~ 250 ms), we computed the number of hit and false alarm trials *N*_hit_(*t*) and *N*_FA_(*t*) for which the first lick fell in that window. The hit and false alarm rates were defined as *r*_hit_ = *N*_hit_(*t*)/(*N*_hit_(*t*) + *N*_FA_(*t*)), *r*_FA_ = *N*_FA_(*t*)/(*N*_hit_(*t*) + *N*_FA_(*t*)) i.e. the fraction of first licks at time *t* relative to stimulus onset that were hits or false alarms.

Lick-triggered averages on false alarm trials were obtained as $\text{LTA}(t)=(1/N_{\text{FAtrials}})\sum_{i\;\in \;\text{FAtrials}}{\hat{w}}^{T}({\text{r}}_{t-t_{l^{\left(i\right)}}}^{\left(i\right)}-{\bar{r}}_{t-t_{l^{\left(i\right)}}}^{\left(A\right)})$, and similarly for hit trials, where $\text{w}=\sum^{-1}({\bar{r}}^{V}-{\bar{r}}^{A})$ and *t*_*l*^(*i*)^_ is the first lick time on trial *i* (selected as described above). This gave a lick-triggered average for each mouse, which we then averaged across mice.

##### Autocorrelation along linear discriminant

Autocorrelations were computed as $R_{\tau }^{\left(i\right)}=\sum_{t=0}^{T-\tau -1}\left(w^{T}\left(r_{t+\tau }^{\left(i\right)}-{\bar{r}}_{t+\tau }^{\left(s\right)}\right)\right)\left(w^{T}\left(r_{t}^{\left(i\right)}-{\bar{r}}_{t}^{\left(s\right)}\right)\right)$ with *T* = 8, which gave an autocorrelation for each animal on each trial computed over the 0 to 1 s interval after stimulus onset (this was implemented using Matlab’s xcorr function). These single-trial autocorrelations were then averaged for each animal and normalized by their zero-lag value to obtain $R_{\tau }^{\left(s\right)}=\sum_{i\;\in \;\text{Trials(s)}}R_{\tau }^{\left(i\right)}/\sum_{i\;\in \;\text{Trials(s)}}R_{0}^{\left(i\right)}$. The area under the curve was quantified as ${\text{AUC}}^{\left(s\right)}=\sum_{\tau =-T}^{T}R_{\tau }^{\left(s\right)}/(2T+1)$. This gave an AUC for each mouse and each stimulus.

##### Principal component analysis of trial-averaged responses

For each time point *t* relative to the vertical stimulus onset, we concatenated the trial-averaged responses of all neurons ${\bar{r}}_{t}^{\left(V\right)}$ across all animals into a single vector **x**_*t*_, and compiled the set of such vectors with time indices from -0.5 to 1 s relative to stimulus onset into a matrix *X* with dimensions *N*×*T* neurons by time samples. We then performed a singular value decomposition on this matrix which gave *X* = *USV^T^*, where *V* contains temporal modes, *U* contains neuron modes describing the evolution of population activity through time, and *S* is a rectangular diagonal matrix containing the singular values describing the amount each component contributes to *X*. We performed this analysis separately on the pre- and post-learning data and plotted the two temporal modes (columns of *V*) with largest corresponding singular values (Figure S2G). Each neuron mode (the columns of *U*) was a vector containing all cells across animals, so for each animal we extracted the corresponding subvector **n** and computed the alignment of this subvector with the animal’s linear discriminant ${\hat{n}}^{T}\hat{w}$, $w={\text{Σ}}^{-1}\left({\bar{r}}^{V}-{\bar{r}}^{A}\right)$. Figure S2H shows the resulting alignments.

##### Analysis of cell types in MVAR model

The dataset comprised simultaneous calcium imaging of pyramidal (PYR), parvalbumin-expressing (PV), somatostatin-expressing (SOM) and vasointestinal peptide-expressing (VIP) interneurons. Thus, the vector of responses at time *t* on trial *i* could be written as $r_{t}^{\left(i\right)}=\left[r_{\text{PYR},t}^{\left(i\right)},r_{\text{PV},t}^{\left(i\right)},r_{\text{SOM},t}^{\left(i\right)},r_{\text{VIP},t}^{\left(i\right)}\right]$, and similarly the output discriminant could be written as **w** = [**w**_PYR_, **w**_PV_, **w**_SOM_, **w**_VIP_], etc. To test whether changes in loading of cell types onto the output linear discriminant occurred with learning, for each mouse we computed the mean squared loading for a given cell type as *L*(**w**, *X*) = (‖**w**_X_‖^2^/‖**w**‖^2^)(*N/N_X_*), where *X* ∈ {PYR, PV, SOM, VIP}, *N* is the length of the vector **w** and *N_X_* is the length of **w**_*X*_ (i.e. the total number of cellsand the number of cells of type *X* for that animal). This measures the fraction of the norm of vector **w** that is generated by cell class *X*, normalized by the fraction of cells in class *X*. Mean squared coupling of neurons into modes was computed in the same way, using the mode vector **m** instead of the linear discriminant **w**. This gave a single value of mean squared loading per animal and cell type for the input and output discriminants, and a single value of mean squared coupling per mode and cell type.

We computed the response of cell type *X* to an input perturbation to cell type *Y* as **w**_*X*_’(*A* + *I_N_*)^*t*^**p**_*Y*_, where **w**_*X*_, **p**_*Y*_ are the sub-vectors of the response discriminant and input discriminant corresponding to cell types *X* and *Y* respectively. Note that our network-level analysis of input perturbations can be decomposed into a sum over these cell class-specific perturbations, i.e. **w**·(*A* + *I_N_*)^*t*^**p** = ∑_*X*,*Y*_**w**_*X*_’(*A* + *I_N_*)^*t*^**p**_*Y*_. Thus, this analysis decomposes the network response to perturbations (Figures 3G–3I) into multiple directed pathways between cell types.

#### Network models (Figures 6, 7, and S5–S7)

##### Model Description

We considered two populationsof cells (excitatory and inhibitory), each arranged on a ring, with *N^X^* cells in population *X* ∈ {*E*, *I*}. Each population is parameterized by its orientation on the ring $\theta_{i}^{X}=2\pi i/N^{X}$. Dynamics were governed by the Wilson-Cowan equation $\tau^{X}(\partial r_{i}^{X}/\partial t)=-r_{i}^{X}+\phi (\sum_{Y-EJ}\sum_{j=1}^{N^{Y}}W_{ij}^{XY}r_{j}^{Y}+u_{i}^{X}(\theta_{s},t))$, where $r_{i}^{X}$ is the firing rate of neuron *i* in population *X*, *τ^t^* is the time constant of neurons in population *X*, $W_{ij}^{XY}$ is the weight from neuron *j* in population *Y* to neuron *i* in population *X*, $u_{i}^{X}(\theta_{s},t)$ is the external input to neuron *i* in population *X* as a function of the stimulus orientation *θ_s_* and time *t*, and *ϕ* is an element-wise nonlinearity. For both *E* and *I* populations we used a threshold-power law nonlinearity $\phi \left(X\right)={\left[X\right]}_{+}^{\text{γ}}$ (Hansel and Van Vreeswijk, 2002; Miller and Troyer, 2002; Ahmadian et al., 2013; Rubin et al., 2015; Hennequin et al., 2018).

External input had stimulus-tuned mean $g_{i}^{X}\left(\theta_{s}\right)$ and additive, temporally uncorrelated Gaussian noise $\eta_{i}^{X}(t)$, i.e. $u_{i}^{X}(\theta_{s},t)=g_{i}^{X}\left(\theta_{s}\right)+\eta_{i}^{X}(t)$ with $\langle \eta_{i}^{X}(t)=0\rangle$ and $\langle \eta_{i}^{X}(t)\eta_{j}^{Y}\left(t^{\prime }\right)\rangle ={\left(\sigma^{X}\right)}^{2}\delta_{ij}\delta_{XY}\delta \left(t-t^{\prime }\right)$. Input tuning curves were circular-Gaussian, rotationally-invariant functions of stimulus orientation, defined by von Mises functions $g_{i}^{X}\left(\theta_{s}\right)=\left(g_{0}^{X}/2\pi I_{0}\left(\kappa^{X}\right)\right)\text{exp}\left(\kappa^{X}\text{cos}\left(\theta_{i}^{X}-\theta_{s}\right)\right)$. The parameter *κ^X^* determines how concentrated the inputs are around the ring (i.e., orientation selectivity of input), while $g_{0}^{X}$ controls the total strength of network input. *I*_0_ is the modified Bessel function of the first kind, which is included to normalize the total input strength so as to be independent of the input tuning *κ^X^*. To preserve rotational symmetry, inputs were chosen such that that $\theta_{s}=\theta_{i}^{E}=\theta_{j}^{l}$ for some pair of integers *i*,*j*.

For the uniform network, weights had the same circular-Gaussian form as the input, $W_{ij}^{XY}=\left(W_{0}^{XY}/I_{0}\left(\kappa^{XY}\right)\right)\text{exp}\left(\kappa^{XY}\text{cos}\left(\theta_{i}^{X}-\theta_{j}^{Y}\right)\right)$ where *κ^XY^*, $W_{0}^{XY}$ are the concentration and strength parameters for the weights from population *Y* to population *X*. For the non-uniform network, the excitatory to inhibitory weights were modified to $W_{ij}^{IE}={\left(W_{\text{uniform}\;}^{IE}+W_{\text{sub}\;}^{IE}\right)}_{ij}\left(\langle W_{\text{uniform}\;}^{IE}\rangle /\langle W_{\text{uniform}\;}^{IE}+W_{\text{sub}\;}^{IE}\rangle \right)$ where $W_{\text{uniform}}^{IE}$ is the connectivity for the uniform network, ${\left(W_{\text{sub}\;}^{IE}\right)}_{ij}=\left(W_{0,\text{sub}\;\;}^{IE}/I_{0}^{2}\left(\kappa_{\text{sub}\;}^{IE}\right)\right)\text{exp}\left(\kappa_{\text{sub}\;}^{IE}\text{cos}\left(\theta_{i}^{I}-\theta_{\text{sub}\;}\right)\right)\text{exp}\left(\kappa_{\text{sub}\;}^{IE}\text{cos}\left(\theta_{j}^{E}-\theta_{\text{sub}\;}\right)\right)$ is the additional subnetwork connectivity, 〈*W*〉 denotes an average over all elements of the weight matrix *W* and κ_sub_, $W_{0,\text{sub}}^{IE}$ are the concentration and strength parameters for the excitatory-inhibitory subnetwork.

##### Parameter settings and modeling assumptions

We modeled external input as being temporally and spatially uncorrelated. This choice was made to aid numerical analysis, to reduce the number of parameters, and to aid interpretability of our findings, but does not qualitatively affect our results. For example, choosing input to be spatially uncorrelated ensured that the response covariance was determined purely by recurrent network dynamics and not inherited through input, which allowed clearer insight into the relationship between dynamics and variability. Spatially correlated input does not influence response tuning or dynamics in our linearized analysis, but does influence the input linear discriminant and therefore also influences the optimal network dynamics. Similarly, if temporal correlations are spatially isotropic, they do not affect our results other than scaling down the response information by a constant factor, while if temporal correlations vary across input dimensions then the optimal solution is for the network to integrate input dimensions with high instantaneous SNR but low temporal correlations (see Methods S1 File). Thus, the temporally uncorrelated input we consider gives an upper bound estimate for the response information of a network that receives input with stimulus-independent temporal correlations.

With the exception of parameter sweeps and Figures S5 and S7G-S7L, all analyses of the uniform and non-uniform network used the following baseline parameters: *N^E^* = 1000, *N^I^* = 200, *τ^E^* = 10, *τ*^I^ = 5, γ = 2, *κ^E^* = 0.5, *κ^l^* = 0, $g_{0}^{E}=0.5,g_{0}^{l}=0,W_{0}^{EE}=0.019,W_{0}^{ll}=-1.1W_{0}^{EE},W_{0}^{EI}=-0.04,W_{0}^{IE}=0.04,\kappa^{EE}=2,\kappa^{ll}=0,\kappa^{lE}=0.1,\kappa^{{}^{EI}}=0.4,\kappa_{\text{sub}\;}^{lE}=4.2,W_{0,sub}^{IE}=0.004,{\left(\sigma^{E}\right)}^{2}=2\sum_{i=1}^{N^{E}}g_{i}^{E}/N^{E},{\left(\sigma^{l}\right)}^{2}={\left(\sigma^{E}\right)}^{2}/2$. For parameter sweeps, all parameters other than those varied were held at these baseline values. In Figure S5, the network with weak sharpening used *κ^EE^* = 1.4, *κ^IE^* = 0.9, while the network with strong sharpening used *κ^EE^* = 2.8, *κ^IE^* = 0.4, with all other baseline parameters unchanged. Figures S7G-S7L used *κ^E^* = 2, $W_{0}^{ll}=-W_{0}^{EE}$, *κ^EE^* = 3, *κ^IE^* = 0.1, *κ^EI^* = 1, *κ*_sub_ = 32, $W_{\text{sub}}^{IE}=0.0005$.

We found that there was substantial flexibility in the parameter settings in that very different parameter configurations often led to qualitatively similar dynamics (see e.g., Figures S6 and S7G–S7L). Thus, while varying individual parameters altered the behavior of the network, this could typically be offset by compensatory changes in other parameters. Wherever possible, our parameter choices were chosen based on experimental data from mouse visual cortex. For example, Hofer et al. (2011) report untuned E to I synapses, while Znamenskiy et al. (2018) report that E to I synapses exhibit some feature tuning but find that this tuning is weaker than I to E or E to E synapses, so we chose to set *κ^EE^* > *κ^EI^* > *κ^IE^*. To the best of our knowledge, there are no data on the feature tuning of I to I synapses, so we set *κ^ll^* = 0. While the net feedforward input to E cells is orientation tuned (Lien and Scanziani, 2013), PV neurons receive very weakly tuned (or untuned) thalamic input (Bereshpolova et al., 2020) and their responses are very weakly tuned to orientation in mouse V1 (Hofer et al., 2011; Kerlin et al., 2010), so we set *κ^E^* > 0 and *k^l^* = 0. Although PV neurons do receive feedforward input (Bereshpolova et al., 2020), we set $g_{0}^{l}=0$ since the modeled input can be interpreted as the tuned component relative to some base-line level or firing threshold. We found that increasing $g_{0}^{l}$ or *κ^l^* decreased the strength and tuning of excitatory network responses and decreased network time constants but did not qualitatively alter our findings. Moreover, these changes could be compensated by increases in recurrent excitation ($W_{0}^{EE}$ or *κ^EE^*) or decreases in inhibition ($W_{0}^{EI},W_{0}^{IE}$) or *κ^EI^*, *κ^IE^*). The magnitude of input noise to E and I neurons *σ^E^*, *σ^I^* were chosen in order to generate similar E and I response SNRs to those measured for PYR and PV neurons in Khan et al. (2018) (note that I cells had broad tuning curves in our model as reported in experiment, see Hofer et al., 2011; Kerlin et al., 2010). The input noise only affects response covariance in our linearized analysis, so varying *σ^E^*, *σ^I^* would not alter the network dynamics or tuning curves. Thus, our choice of parameters was broadly consistent with known data, but there was substantial freedom in the precise configuration, so that our results were not dependent on fine-tuning of individual parameters.

Nonetheless, there were two general criteria that required some mild tuning of (sets of) parameters. First, the network was required to exhibit integration time constants longer than those of individual neurons, which occurred when recurrent excitation was sufficiently strong and tuned relative to recurrent inhibition (Figure S6). Second, the non-uniform inhibition mechanism required that I to E input was sufficiently tuned to repel the E response bump away from the subnetwork center, which required that inhibition onto E cells was sharply tuned relative to the width of the response bump. In Figures S7G-S7L where the response bump was much narrower than in our standard parameter setting, this was achieved by setting E to I weights to be broadly tuned (which enabled strong recurrent excitation and long time constants, see Figure S6) and I to E weights to be more narrowly tuned (which ensured sharply tuned inhibition of E cells). An alternative parameter setting in which both E to I and I to E were broadly tuned achieved the same result provided that a narrowly tuned I to E subnetwork formed in addition to the E to I subnetwork (not shown).

We note that with our baseline parameters the network was in the “marginal regime” (Ben-Yishai et al., 1995) — when the input was replaced with an untuned input with same mean strength over neurons, the network spontaneously formed a stable bump of activity, albeit weaker and more broadly tuned than the bump driven by tuned input. When these untuned inputs were decreased slightly in amplitude the bump no longer formed, suggesting that the network was near the boundary of the marginal regime (see also Ponce-Alvarez et al., 2013).

Finally, we note that the parameters for the non-uniform network in Figures 6 and 7 were chosen to demonstrate the effect of non-uniform inhibition as clearly as possible. In particular, while the suppression of responses in Figure 6E and separation of responses in Figure 7A are large in magnitude, this should be understood as the most extreme parameter setting along a continuum of networks shown in Figure S6E. Indeed, other parameter settings in Figure S6E showed milder but qualitatively similar effects on response tuning, and the parameter setting of Figures S7G–S7L showed a more modest separation of responses than that of Figure 7A while still generating a similar improvement in alignment of modes and response SNR. Thus, our simulations were designed to illustrate the qualitative behavior of the proposed mechanism over a wide range of parameters, rather than to provide a close quantitative match to experimental data for a specific set of parameters.

##### Analysis of Linearized Dynamics

To compute modes of linearized dynamics and their time constants we used numerical methods to find the fixed points of the network dynamics and then numerically computed the eigenvalues and eigenvectors of an analytically-derived Jacobian.

We found that fixed point estimates obtained by forward-simulating with the Euler method yielded inaccurate estimates of linearized dynamics. Instead, we found the fixed points of the network using a root-finding algorithm applied to the equation $\dot{r}=0$, where **r** = [**r**^*E*^; **r**^*I*^], *W* = [*W^EE^*, *W^EI^*; *W^IE^*, *W^II^*] etc., *T* is a diagonal matrix of neuronal time constants, and $\dot{r}=T^{-1}(-r+\phi (Wr+g))$. We used Newton’s method with the analytically-derived Jacobian $J(r)\equiv \partial \dot{r}/\partial r=\text{Φ}\prime W-T^{-1}$ (where *Φ*’ = *T*^-1^ diag(γ*ϕ*(*W***r** + **g**)^1–1/*γ*^) for our choice of transfer function). Fixed point estimates **r**_*n*_ were iteratively updated as $r_{n+1}=r_{n}-J^{-1}\left(r_{n}\right){\dot{r}}_{n}$. The algorithm was terminated when $\left\|{\dot{r}}_{n}\right\|<10^{-15}$ (where it was considered to have converged), or after 100 iterations (which was classed as a failure to converge). The root-finding algorithm was initialized at **r**_0_ = 0 (or when performing a parameter sweep, at the fixed point obtained from the previous set of parameters).

Having found a fixed point, the time constants, input SNRs, and output SNRs of linearized dynamical modes were computed using analytically-derived equations *τ_i_* = – 1/Real(*γ_i_*), ${\text{SNR}}_{\text{input}\;}\left({\tilde{v}}_{i}^{L}\right)=\left|{\tilde{v}}_{i}^{L}\cdot g^{\prime }\left(\theta_{s}\right)\right|/\sqrt{{\tilde{v}}_{i}^{L}\cdot {\text{Σ}}_{\eta }{\tilde{v}}_{i}^{L}},{\text{SNR}}_{\text{output}\;}\left(v_{i}^{L}\right)={\text{SNR}}_{\text{input}\;}\left({\tilde{v}}_{i}^{L}\right)\sqrt{2\tau_{i}}$, where *γ_i_*, $v_{i}^{L}$, are eigenvalues and left eigenvectors of the Jacobian *J* = *Φ*’*W* − *T*^-1^, and ${\tilde{v}}_{i}^{L}$ are the left eigenvectors of the matrix $\tilde{J}=W\Phi '-T^{-1}$. Note that ${\tilde{\lambda }}_{i}=\lambda_{i}$, and that *Φ*’ = *T*^-1^ diag(*γ***r**^1–1/*γ*^) at the fixed point (see Methods S1 File). Where modes are explicitly plotted (Figures 6B, 6C, 6E, S5A-S5C, S5G-S5I, S7A, S7B, and S7H), the quantities shown are the elements of ${\tilde{v}}_{i}^{L}$. The normalized input SNR was computed as ${\text{SNR}}_{\text{norm}\;}\left({\tilde{v}}_{i}^{L}\right)={\text{SNR}}_{\text{input}\;}\left({\tilde{v}}_{i}^{L}\right)/\sqrt{g^{\prime }\left(\theta_{s}\right)\cdot \sum_{\text{η}}^{-1}g^{\prime }\left(\theta_{s}\right)}$. The degree of recurrent sharpening was quantified as $N^{E}/N_{+}^{E}-1$, where $N_{+}^{E}$ is the number of excitatory neurons with non-zero firing rate at the fixed point. Mean squared coupling of excitatory and inhibitory neurons into the translation mode was computed as described above for the experimental data. Mean squared loading onto the linear discriminant was computed using the discriminant vector $w={\tilde{\Sigma }}^{-1}r^{\prime }$, where $\tilde{\Sigma }=\text{Σ}+\epsilon I_{N}$ is the response covariance plus a small amount of “observation noise” which was added to avoid excessively large discriminant loadings for neurons with very low firing rates (see Seung and Sompolinsky, 1993). We set $\epsilon =0.01{\text{Σ}}_{i=1}^{N}r_{i}/N$

##### Analysis of two-stimulus discrimination and nonlinear dynamics

Our theoretical results are underpinned by two key approximations: the linearization of network dynamics about a fixed point and the analysis of stationary state response statistics of the linearized system. The linearization of dynamics restricts the domain of application of our theory to fine-scale sensory discrimination tasks, whereas the stimuli presented experimentally were separated by 40°. We therefore sought numerically determine whether our linearized theory provides adequate insight into the full nonlinear and non-stationary integration of the experimentally presented stimuli through the recurrent network. We took two approaches to do this. First, to determine the stationary state response information for two stimuli separated by 40°, we separately computed the linearized stationary state response statistics about each stimulus (Figures 7A and S7C-S7E) and then used linear discriminant analysis to compute response information. Second, to determine the non-stationary integration of input through the network dynamics following stimulus onset, we numerically computed responses of the nonlinear system over time using the Euler method (Figure 7B). The behavior of the linearized system made predictions that we were able to confirm in simulations of the nonlinear system: recurrent sharpening caused the most slowly-decaying mode to increase its time constant and become less aligned with the input discriminant (Figure S5), which predicts that input information should be integrated more slowly but over a longer time window, and should nonetheless achieve a greater stationary state information relative to the non-sharpened network; similarly, non-uniform inhibition caused the most slowly-decaying mode to become better aligned to the input discriminant without changing its time constant (Figures 6E–6H), which predicts that input information should be integrated more rapidly, with response information reaching its plateau before the sharpened or baseline uniform network. Both predictions were borne out in simulations of the non-stationary nonlinear dynamics (Figure 7B), which demonstrates that the linearized stationary state approximation to the network dynamics captures the integrative behavior of the nonlinear non-stationary system. We then verified that the same qualitative behavior could be observed in the data (Figure 7C), as would be expected based on the observed changes in MVAR modes (Figure 4).

For Figures 7A and S7C-S7E we computed the fixed points and Jacobians associated with the two stimulus orientations *θ*_*s*1_ = *θ*_sub_ – 20°, *θ*_*s*2_ = *θ*_sub_ + 20°. We computed stationary state response covariance around each of these fixed points by numerically solving the corresponding Lyapunov equation *J*∑ + ∑*J^T^* + Φ’∑_η_Φ’ = 0. We computed response information as *I* = (**r**(*θ*_*s*2_) - **r**(*θ*_*s*1_))·[(1/2)(*Σ*(*θ*_*s*1_) + *Σ*(*θ*_*s*2_))]^-1^ (**r**(*θ*_*s*2_) - **r**(*θ*_*s*1_)). Response information was then normalized by the response information computed for a network with $W_{0,\text{sub}}^{IE}=0$ (computed using the same method with all other parameters unchanged). The SNR of excitatory and inhibitory responses were computed as $SNR_{i}^{X}=(\left|r_{i}^{X}\left(\theta_{s_{2}}\right)-r_{i}^{X}\left(\theta_{s_{1}}\right)\right|/\sqrt{\left(1/2)({\text{Σ}}_{ij}^{X}(\theta_{s_{1}})+{\text{Σ}}_{ij}^{X}(\theta_{s_{2}})\right)})$. In Figures S7C and S7D, we plotted ${\left((1/N^{X})\sum_{i=1}^{N^{X}}{\text{SNR}}_{i}^{X}\right)}^{2}$ normalized by its value in the network with $W_{0,\text{sub}}^{IE}=0$ in order to facilitate direct comparison with the response information. In Figure S7E we plotted the unnormalized $(1/N^{X})\sum_{i=1}^{N^{X}}{\text{SNR}}_{i}^{X}$ to facilitate comparison with previously defined measures of neuronal response SNR (see Khan et al., 2018, in which this measure is reported as the mean absolute selectivity).

To investigate the non-stationary and non-linear integration of sensory input following stimulus onset, we numerically solved the Wilson-Cowan equation using the Euler method. We used a time step of *dt* = 1 and initialized the simulation at the fixed point **r**(*θ*_sub_) with external input given by one of the two stimuli *θ*_*S_i_*_ = *θ*_sub_ ± 20°. At each time step we computed the projection of responses onto the stationary state linear discriminant $d\left(t,\theta_{s_{i}}\right)=w_{LD}^{T}r\left(t,\theta_{s_{i}}\right)$, with **w**_*LD*_ = [(1/2)(*Σ*(*θ*_*s*1_) + *Σ*(*θ*_*s*2_))]^-1^(**r**(*θ*_*s*2_) – **r**(*θ*_*s*1_)) computed using the analytical equations for the stationary state means and covariances in the linearized systems about each fixed point. We simulated 1000 trials with 1000 time steps each. We computed the signal-to-noise ratio of this quantity as $\text{SNR}(t)=\langle d(t,\theta_{s_{2}})-d(t,\theta_{s_{1}})\rangle /\sqrt{0.5\left[\text{Var}\left(d\left(t,\theta_{s_{1}}\right)\right)+\text{Var}\left(d\left(t,\theta_{s_{2}}\right)\right)\right]}$, where averages and variances were taken over trials at each point in time. Forthe baseline and non-uniform networks we set *κ^EE^* = 1.8, and forthe sharpened network *κ^EE^* = 2. Forthe non-uniform network we set $\kappa_{sub}^{IE}=4.2,W_{0,\text{sub}}^{IE}=0.004$ and forthe baseline and sharpened network $\kappa_{sub}^{IE}=0,W_{0,\text{sub}}^{IE}=0$. We normalized SNR(*t*) by the average value in the final 300 time steps under the baseline network model.

To compute response SNR as a function of time in the experimental data, we computed the linear discriminant as ${\text{w}}_{\text{LD}}=\sum^{-1}({\bar{r}}^{V}-{\bar{r}}^{A})$ where *Σ* and ${\bar{r}}^{\left(s\right)}$ were computed as in Figure 3. We projected imaged responses $r_{t}^{\left(i\right)}$ onto **w**_LD_ at each time point *t* on each trial for the vertical and angled stimuli to obtain $d_{t}^{\left(i\right)}=w_{LD}^{T}r_{t}^{\left(i\right)}$. We computed the signal-to-noise ratio of this projection at each time point relative to stimulus onset by computing its mean difference between stimuli and its pooled standard deviation across stimuli, i.e. ${\text{SNR}}_{t}=\left|{\langle d_{t}^{\left(i\right)}\rangle }_{i\;\in \;\;\;\text{Trials}(V)}-{\langle d_{t}^{\left(i\right)}\rangle }_{i\;\in \;\;\;\text{Trials}(A)}\right|/\sqrt{0.5[\text{Var}{\left(d_{t}^{\left(i\right)}\right)}_{i\;\in \;\;\;\;\text{Trials}(V)}+\text{Var}{\left(d_{t}^{\left(i\right)}\right)}_{i\;\in \;\;\;\;\text{Trials}(A)}]}$. We performed this analysis separately for the pre- and post-learning data for each animal. The SNR ratio (Figure S2F) was computed as ${\text{SNR}}_{t}^{\text{post}\;}/{\text{SNR}}_{t}^{\text{pre}\;}$ for each animal and then averaged over animals. We compared this to predictions from the dynamical slowing and dynamical realignment hypotheses (Figure S2F inset) by computing analytically the SNR of responses along a dynamical mode **m** with time constant *τ* as a function of time from stimulus onset, ${\text{SNR}}_{\text{output}\;}(m,t)={\text{SNR}}_{\text{input}\;}(m)\sqrt{2\tau }\sqrt{\left(1-e^{-t/\tau })/(1+e^{-t/\tau }\right)}$. We plotted the ratio SNR_output,post_(**m**, *t*)/SNR_output,pre_(**m**, *t*) by setting either *τ*_pre_ = *τ*_post_ = 0.5, SNR_input,pre_(**m**) = 1, SNR_input,post_(**m**) = 1.5 (dynamical realignment) or *τ*_pre_ = 0.5, *τ*_post_ = 1.125, SNR_input,pre_(**m**) = SNR_input,post_(**m**) = 1 (dynamical slowing).

##### Comparison of response changes to preferred and non-preferred stimuli in model and data

We computed the change in the response of excitatory and inhibitory cells to their preferred and non-preferred stimuli over learning (in the experimental data) and between the uniform and non-uniform ring network models.

In the network models, we defined the preferred stimulus of excitatory cell *i* as the stimulus which generates the greater firing rate value at the fixed point, i.e. $\theta_{\text{pref}\;}(i)={\text{argmax}}_{\theta_{s_{k}}}\left[r_{i}^{E}\left(\theta_{s_{k}}\right)\right]$ where *k* = 1,2. The change in response to its preferred stimulus was defined as the difference in response between the two networks, i.e. $\Delta r_{i}^{E}\left(\theta_{\text{pref}\;}(i)\right)={\left[r_{i}^{E}\left(\theta_{\text{pref}\;}(i)\right)\right]}_{\text{non}\;\text{-}\;\text{uniform}\;}-{\left[r_{i}^{E}\left(\theta_{\text{pref}\;}(i)\right)\right]}_{\text{uniform}\;}$ (note that cells did not change stimulus preference). The mean and variance of this change in response were then taken over the population of excitatory cells, i.e. $\text{mean}\left[\Delta r^{E}\left(\theta_{\text{pref}\;}\right)\right]=\left(1/N^{E}\right)\sum_{i=1}^{N^{E}}\Delta r_{i}^{E}\left(\theta_{\text{pref}\;}(i)\right)$, and $\text{var[}\Delta r^{E}(\theta_{\text{pref}\;})\text{]}=(1/N^{E})\sum_{i=1}^{N^{E}}{\left[\Delta r_{i}^{E}(\theta_{\text{pref}\;})-\text{mean[}\Delta r^{E}\text{(}\theta_{\text{pref}\;})\text{]}\right]}^{2}$. The non-preferred stimulus was analyzed similarly but with $\theta_{\text{non-pref}\;}(i)={\text{argmin}}_{\theta_{s_{k}}}\left[r_{i}^{E}\left(\theta_{s_{k}}\right)\right]$.

In the experimental data we considered learning-related response changes of putative pyramidal cells to the vertical and angled grating corridors (see Khan et al. for how cells were identified). For each cell, we computed the difference in its response to the vertical and angled stimuli both pre- and post-learning $\Delta_{\text{V}-\text{A}}{\bar{r}}_{l}={\bar{r}}_{l}^{V}-{\bar{r}}_{l}^{A}$ (where *l* = pre, post). We also computed the change in response to the vertical and angled stimulus over learning $\Delta_{\text{post}\;-\;\text{pre}\;}{\bar{r}}^{\left(s\right)}={\bar{r}}_{\text{post}\;}^{\left(s\right)}-{\bar{r}}_{\text{pre}\;}^{\left(s\right)}$. (where *s* = *A*, *V*). We then took the mean and variance of $\Delta_{\text{post-pre}\;}{\bar{r}}^{\left(s_{\text{pref}\;}\right)}$ over all pyramidal cells which passed a set of inclusion criteria (where $s_{\text{pref}\;}={\text{argmax}}_{s}\left[{\bar{r}}_{l}^{\left(s\right)}\right]$ is the preferred stimulus of the cell). The inclusion criteria were as follows: the cell had a significant preference for one of the vertical and angled stimuli both before and after learning (defined as *p* < 0.05 under a Wilcoxon rank sum test on the responses on vertical vs angled trials); the preferred stimulus *s*_pref_ was the same before and after learning. These criteria were necessary to avoid confounds relating to regression to the mean. The same analysis was performed for the non-preferred stimulus, in this case using $s_{\text{non–pref}\;}={\text{argmin}}_{s}\left[{\bar{r}}_{l}^{\left(s\right)}\right]$.

We computed the average response SNR of individual E and I cells in both the model and data (Figures S7D and S7E). The method for computing E and I response SNR in the network models is described in the above section. Quantification of mean SNR of individual pyramidal and parvalbumin cells was similar and has been reported in Khan et al. (2018).

##### Replication of Schoups et al

For each stimulus orientation *θ_s_*, we computed the fixed point of the network dynamics **r**(*θ_s_*) as described above and computed its derivative **r**'(*θ_s_*) = – *J*^–1^*Φ*'**g**'(*θ_s_*). For each excitatory neuron *i*, we plotted the relative slope of its tuning curve at the trained orientation (corresponding to the subnetwork center) as $\left|r_{i}^{'}(\theta_{\text{sub}})\right|/{\text{max}}_{\theta_{s}}(r_{i}(\theta_{s}))$.

## Supplementary Material

Supplemental information can be found online at https://doi.org/10.1016/j.neuron.2022.10.001.

Supplement

## Data Availability

The data reported in this study are available from the corresponding authors upon request. All original code has been deposited at https://zenodo.org/record/7109995 and is publicly available as of the date of publication. The DOI is listed in the key resources table.

## Highlights

- A new theoretical principle links recurrent circuit dynamics to optimal sensory coding
- Predicts that high-SNR input dimensions activate slowly decaying modes of dynamics
- Population dynamics in primary visual cortex realign during learning as predicted
- Stimulus-specific changes in E-I connectivity in recurrent circuits explain realignment

## In brief

Chadwick et al. develop a theory to determine how the dynamics of a neural circuit can be tuned to optimally integrate behaviorally relevant sensory input. Through analysis of large-scale neural recordings from mouse visual cortex, they demonstrate that neural population dynamics reorganize with learning to enhance cortical representations of taskrelevant stimuli.
