# Supplementary material for: Learning shapes cortical dynamics to enhance integration of relevant sensory input
Source: Neuron. Author manuscript; Available in PMC 2023 Jun 22. (PMC7614688; doi:10.1016/j.neuron.2022.10.001)
Supplement: Supplement [file EMS177372-supplement-Supplement.pdf]

Neuron, Volume *111*

Supplemental information

Learning shapes cortical dynamics to enhance integration of relevant sensory input

Angus Chadwick, Adil G. Khan, Jasper Poort, Antonin Blot, Sonja B. Hofer, Thomas D. Mrsic-Flogel, and Maneesh Sahani

Supplementary Figures

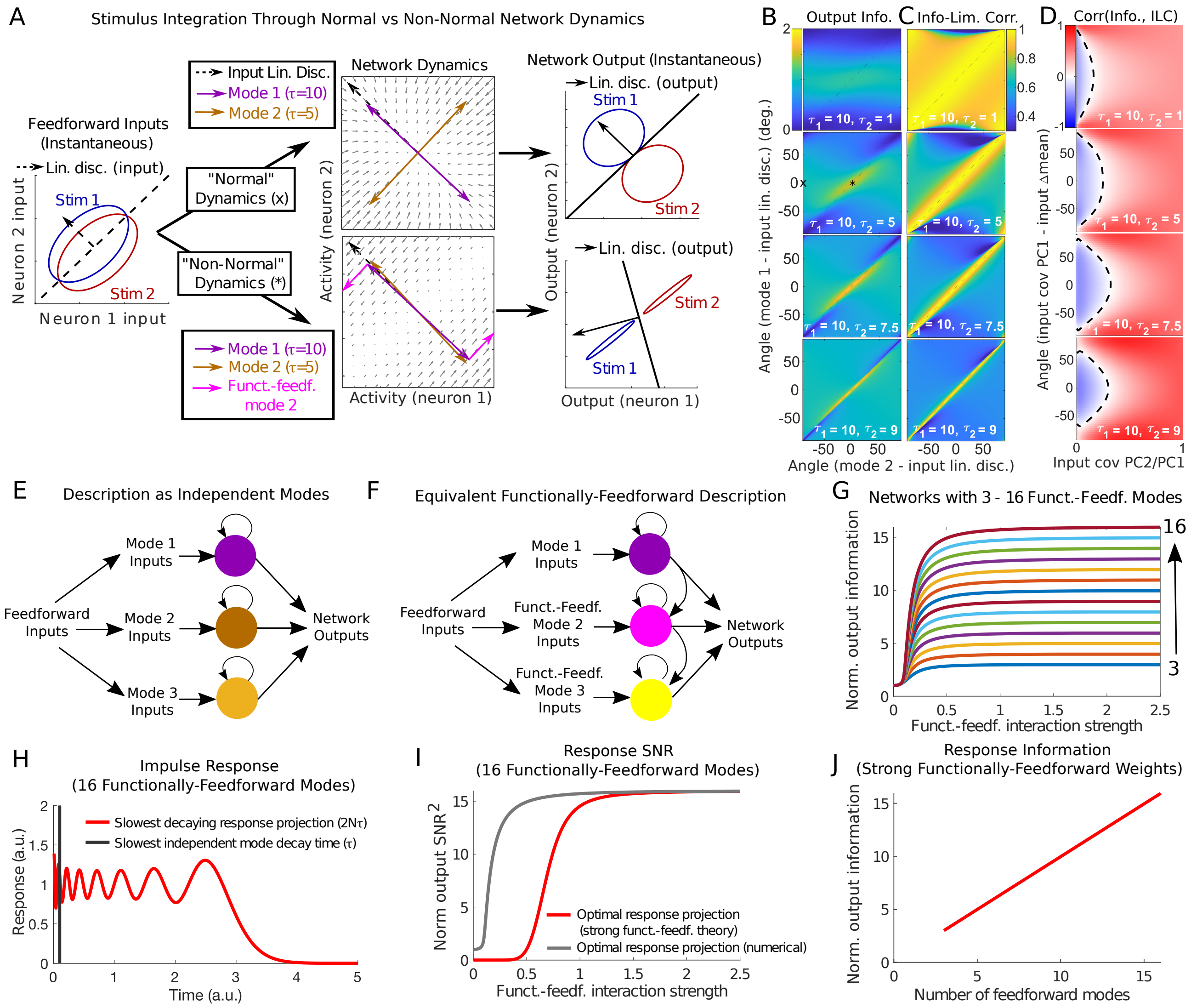

Figure S1. Non-normal dynamics can increase response information through functionally-feedforward temporal integration of the optimal input discriminant (Related to Figure 2). A: Integration of feedforward input through normal and non-normal dynamics. Left: Distributions of instantaneous feedforward input for two stimuli and their linear discriminant (dashed black arrow). Middle: Recurrent dynamics around an input-driven fixed point. Non-normal dynamics can be described by either independent modes or functionally-feedforward modes (Schur decomposition or Jordan normal form; see panels E, F). Right: Distributions of instantaneous network output following integration of feedforward input (solid black arrow is the output linear discriminant). B: Response information depends on the time constants and the activation patterns of modes. x and * are the parameters for the two example networks shown in A (note that an isotropic input covariance was used in B, C). Response information is normalized by the maximum information achievable in a normal network with the same time constants, so the maximum normalized response information of 2 means that the optimal non-normal network performs twice as well as the optimal normal network. Maximum response information occurs when both modes are aligned to the input discriminant and have similar time constants. C: Information-limiting correlations for the networks shown in B (defined as the fraction of total response variance that lies along the direction that separates the two stimulus-driven means). Note that information-limiting correlations are high when response information is high. This is caused by a greater mean separation despite an increased variance along the direction separating the means, as can be seen in the rightmost panels in A. D: Correlation between information-limiting correlations (ILC) and response information as a function of the input covariance. Each pixel shows the Pearson correlation between ILC and response information for a fixed input

covariance matrix and fixed pair of dynamical time constants, where the correlation is taken across all possible angles of the two dynamical modes (i.e., the correlation between two matrices such as shown in each row of B, C). The ratio of the two eigenvalues of the input covariance is varied along the x-axis, while the angle of the principal eigenvector of the input covariance matrix relative to the vector separating the mean inputs for each stimulus is varied along the y-axis. There is a positive correlation between ILC and response information for most input covariance matrices, but a negative correlation occurs when the input covariance is highly anisotropic and points in the direction separating the two means (shown as region inside dashed black lines - note that this corresponds to the regime in which the input ILCs are strong). E-J: response information in higher-dimensional non-normal networks. E, F: Characterization of network dynamics by independent modes (eigenvectors) or "functionally-feedforward" modes (e.g., Schur decomposition). Both are valid descriptions of the dynamics, but functionally-feedforward modes reveal non-normal integration more clearly. G: Response information for networks with varying numbers of functionally-feedforward modes and strength of functionally-feedforward interactions. Information is maximized in networks with strong functionally-feedforward dynamics and grows with the number of modes. H: Response of a strong functionally-feedforward network to a pulse of input. Black line shows the decay time constant of individual modes and red trace shows the timecourse of the most slowly decaying projection of network output. I: Squared SNR of two projections of network outputs. Red shows the optimal projection derived analytically assuming infinitely strong functionally-feedforward weights. Gray curve shows the optimal projection computed numerically for finite weights. J: Response information increases linearly with number of functionally-feedforward modes.

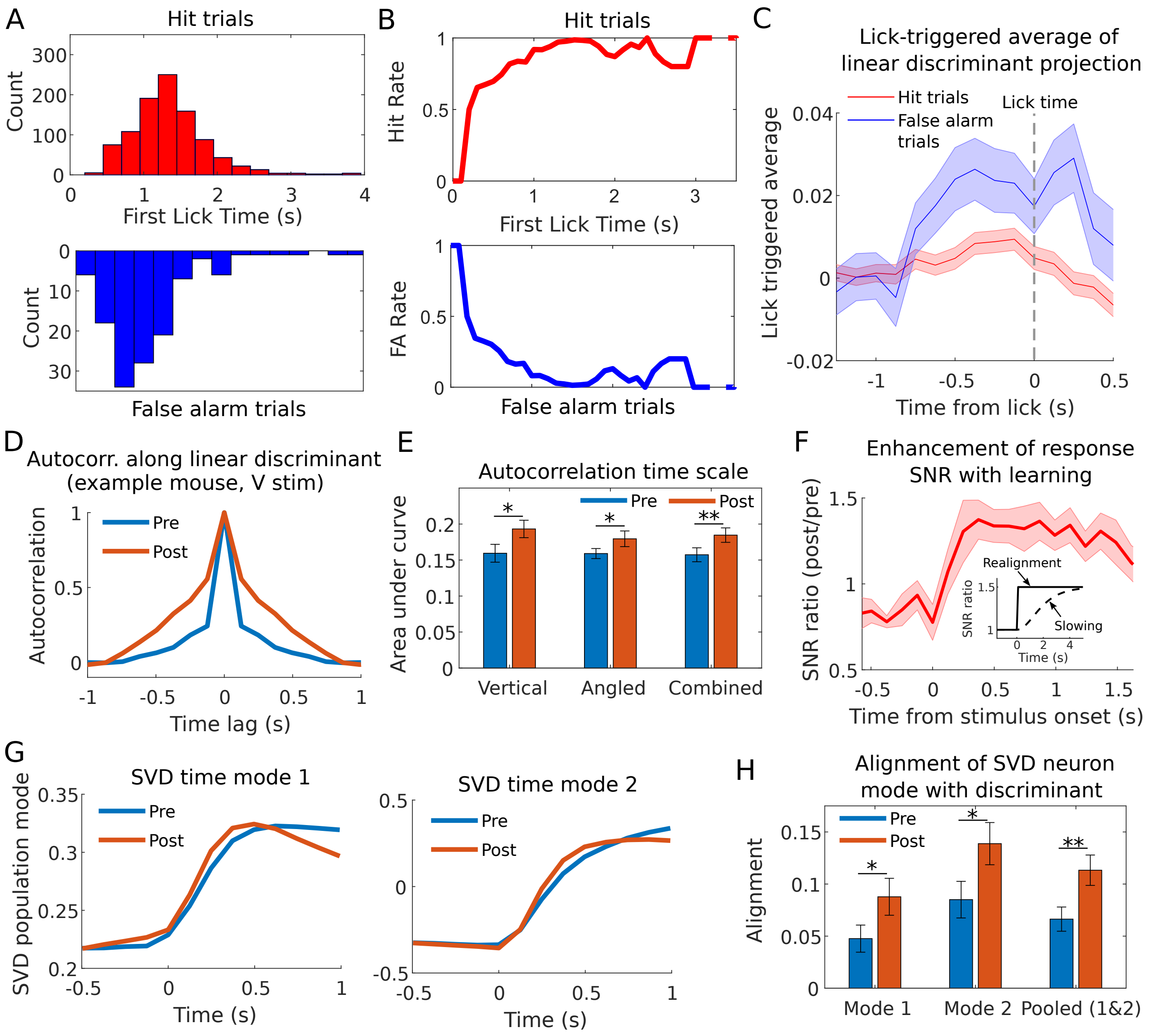

Figure S2. Behavior and neural population activity showed signatures of temporal integration (Related to Figures 3-5). A: Reaction times were faster on false alarm trials than hit trials. B: Hit rates increased and error rates decreased with time from stimulus onset, with timecourses consistent with a leaky integrator of sensory input. C: A lick-triggered average over the projection of population activity along the response discriminant showed that activity ramped up towards the rewarded stimulus on false alarm trials (note that the stimulus-conditioned trial average at each time relative to stimulus onset is subtracted before computing the lick-triggered average, so that a positive deflection on false alarm trials implies an increase relative to correct rejection trials, while the more modest positive deflection on hit trials reflects the fact that very few miss trials occurred, so that the average over hit trials was approximately equal to the average across all rewarded stimulus trials). D, E: There was a significant increase in the timescale of autocorrelation of population activity projected along the linear discriminant. D shows an example mouse for the vertical stimulus, E shows average autocorrelation over mice for the vertical and angled stimuli and for trials pooled over the two stimuli (quantified as area under autocorrelation function with zero-lag normalized to 1; vertical stimulus, $p = 0.046$; angled stimulus, $p=0.029$; autocorrelation of data pooled over stimuli, $p=0.006$, all one-sided paired t-tests on $n=8$ mice). F: Dividing the pre- and post-learning response SNRs (as in Figure 7C) revealed an approximately constant scaling, as predicted by the dynamical realignment but not dynamical slowing hypothesis (shown in inset). Specifically, given the equation $\mathrm{SNR}_{\mathrm{output}}(\mathbf{m}) = \mathrm{SNR}_{\mathrm{input}}(\mathbf{m})\sqrt{2\tau}$, dynamical slowing involves an increase in $\mathrm{SNR}_{\mathrm{input}}(\mathbf{m})$ but not τ, whereas dynamical slowing involves an increase in τ but not $\mathrm{SNR}_{\mathrm{input}}(\mathbf{m})$. The inset shows a theoretical prediction from dynamical slowing (dashed line, $\tau_{\mathrm{pre}} = 0.5$ s, $\tau_{\mathrm{post}} = 1.125$

s, $\mathrm{SNR}_{\mathrm{input,pre}} = \mathrm{SNR}_{\mathrm{input,post}} = 1$) and dynamical realignment (solid line, $\tau_{\mathrm{pre}} = \tau_{\mathrm{post}} = 0.5$ s, $\mathrm{SNR}_{\mathrm{input,pre}} = 1$, $\mathrm{SNR}_{\mathrm{input,pre}} = 1.5$). Note that for these time constants, which were chosen based on the relevant modes in the data (Figure 5), it takes 5 seconds for the SNR ratio to plateau under dynamical slowing, whereas the data plateau within 2 imaging frames (250 ms). G: Singular value decomposition (SVD) applied to the trial-averaged responses of the population of neurons, yielding a set of coupled time x neuron modes that change over learning. The top two modes did not substantially change their timecourse with learning, as would be predicted by the dynamical slowing hypothesis. H: The corresponding neuron modes increased their overlap with the response discriminant over learning, consistent with the dynamical realignment hypothesis. Pooling the overlaps across both modes revealed an even stronger effect (first mode: p=0.02, one-sided paired t-test; second mode: p=0.04, one-sided paired t-test; pooled first two modes: p=0.0035, one-sided paired t-test).

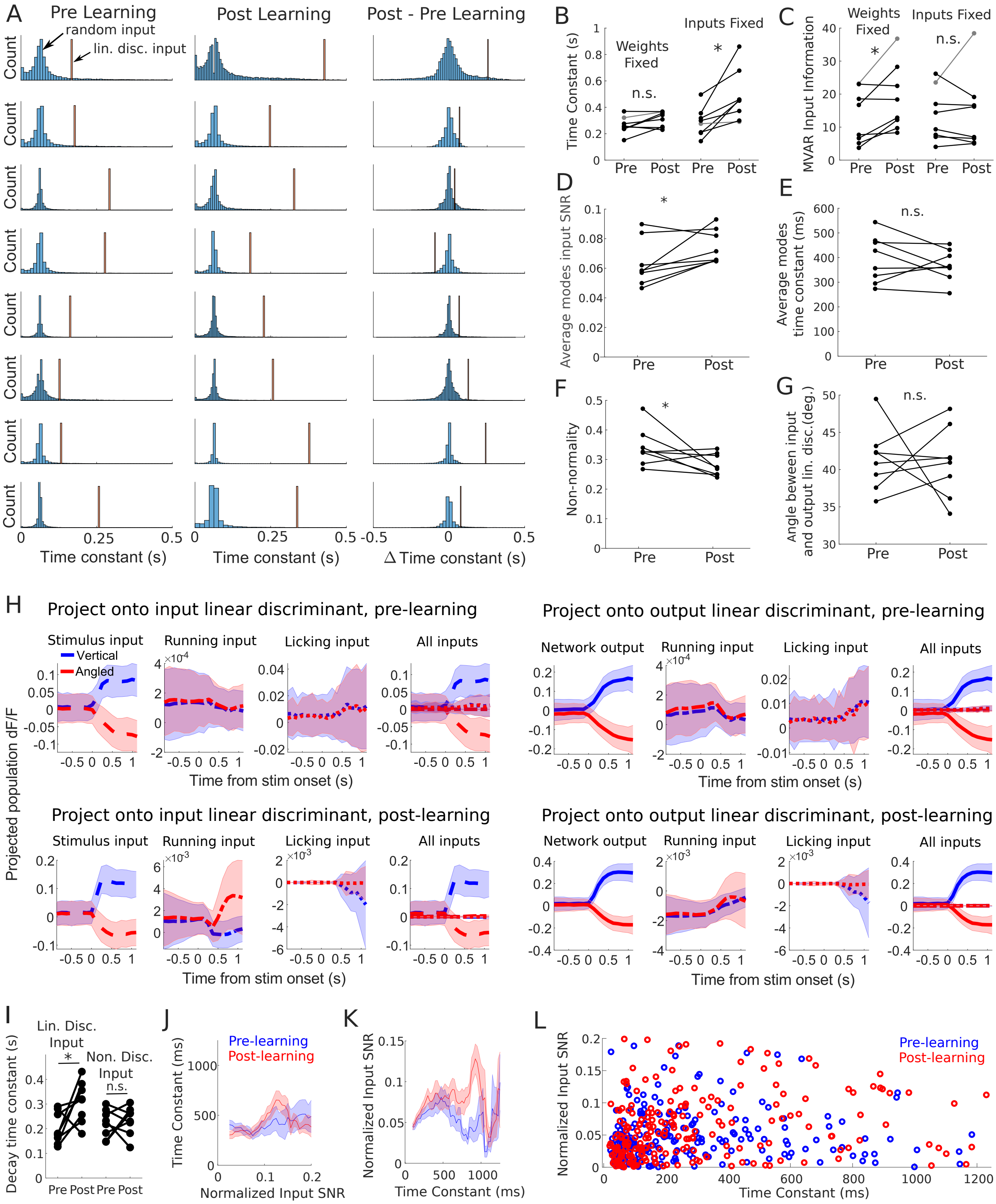

Figure S3. Reorganization of MVAR dynamics for each animal and influence of mouse behaviour. (Related to Figures 3-5). A: Improvements in temporal integration were selective for task-relevant sensory input. Histograms show the network decay time constant in response to a random input perturbation (generated as a random combination of the vertical and angled grating stimulus input for each neuron), overlaid with the decay time of the network response to the linear discriminant input. Each row shows an individual mouse. Column 1 shows time constants before learning, column 2 shows time constants after learning, and column 3 shows the change in time constant over learning for a given random input and for the discriminant input. While all mice showed longer decay time constants for the discriminant input than for random inputs both before and after learning, most mice also showed a greater increase over learning in the decay time constant of responses to the linear discriminant than to random inputs (fraction of random inputs that elicited a decay time con-

stant which increased less than the discriminant input for each mouse: 0.97, 0.91, 0.85, 0.07, 0.94, 0.98, 0.99, 0.94). B, C: Improvements in temporal integration relied on reorganization of interaction weights but not stimulus-related input. B: Time constant of response to input along linear discriminant for an MVAR model in which interaction weights or stimulus-related input was constrained to be the same before and after learning. Gray line shows mouse whose time constant decreased over learning when all parameters were free (see Figure 3E, F, I). C: Information in stimulus-related input to MVAR model. Input information increased when weights were fixed, but not when input was fixed (note that input information could in principle improve through altered residuals even when mean input is held fixed). D-G: Individual mice showed an increase in alignment of modes with the input linear discriminant, no increase in decay time constants, and a decrease in non-normality. D: Average over modes' normalized input SNR, shown for each mouse pre- and post-learning. E: Average over modes' time constant for each mouse. F: Non-normality of interaction weight matrices for each mouse pre- and post-learning. G: Angle between the input linear discriminant and output linear discriminant for each mouse before and after learning. A larger angle would be generated if functionally-feedforward links were formed between task-relevant sensory input and network output (see Figure S1A). There was no change in angle with learning ($p=0.8$, Wilcoxon rank sum test on pre-vs post-learning angle). H: MVAR models fit with a lick-dependent term and with both velocity and licking coefficients free to change over learning. Panels show the contribution of each MVAR term projected onto the linear discriminant (of MVAR input or network responses, pre- or post-learning). Note difference in y-axis scale. The MVAR model suggests a negligible influence of running and licking on population responses along the discriminant projection. I-L: Inclusion of learning-dependent running and licking coefficients did not alter learning-related changes in recurrent integration of task-relevant sensory input. I: Replication of Figure 3I (linear discriminant input, $p=0.035$, non-discriminating input, $p=0.64$, one-sided sign test on all mice). J: Replication of Figure 5A ($p=0.65$, Wilcoxon rank sum test on all modes pooled across mice). K: Replication of Figure 5C ($p<0.05$, Wilcoxon rank sum test on all modes pooled across mice). L: Replication of Figure 5E.

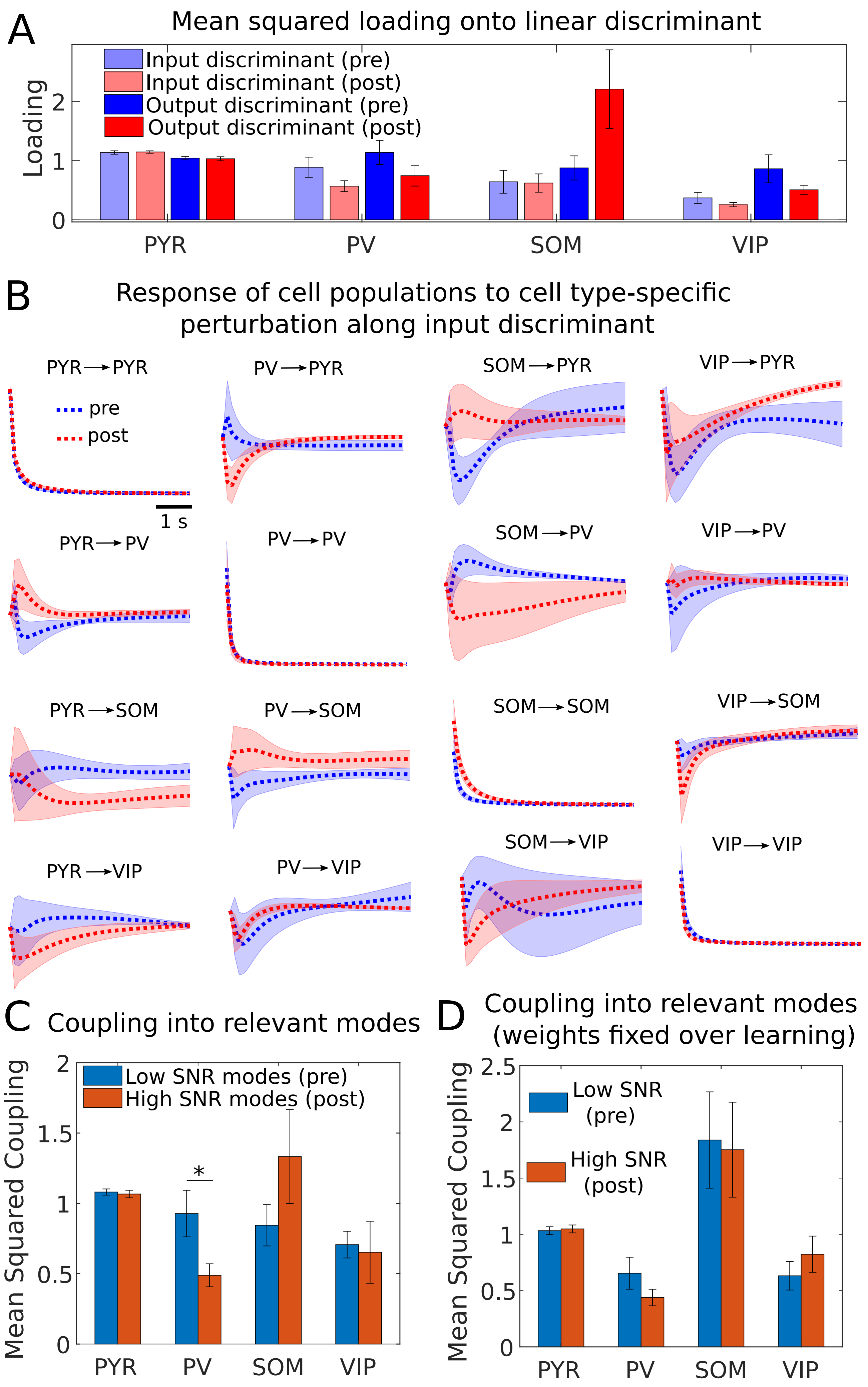
A
Mean squared loading onto linear discriminant
Input discriminant (pre)
Input discriminant (post)
Output discriminant (pre)
Output discriminant (post)
Loading
0
1
2
PYR
PV
SOM
VIP
B
Response of cell populations to cell type-specific perturbation along input discriminant
PYR⟶PYR
pre
post
1 s
PV⟶PYR
SOM⟶PYR
VIP⟶PYR
PYR⟶PV
PV⟶PV
SOM⟶PV
VIP⟶PV
PYR⟶SOM
PV⟶SOM
SOM⟶SOM
VIP⟶SOM
PYR⟶VIP
PV⟶VIP
SOM⟶VIP
VIP⟶VIP
C
Coupling into relevant modes
Low SNR modes (pre)
High SNR modes (post)
Mean Squared Coupling
0
0.5
1
1.5
2
*
PYR
PV
SOM
VIP
D
Coupling into relevant modes (weights fixed over learning)
Low SNR (pre)
High SNR (post)
Mean Squared Coupling
0
0.5
1
1.5
2
2.5
PYR
PV
SOM
VIP

Figure S4. Recruitment of cell types into task-relevant MVAR dynamics (Related to Figures 3-5). A: Loading of cell types onto the linear discriminant. The scale is normalized such that a mean squared loading of 1 would occur if all cells loaded equally. Statistics were performed on a per-animal basis (mean±sem over 8 mice). There were no changes in mean-squared loading with learning for any cell type, suggesting that the distribution of input information and response information across cell types did not change (input/output linear discriminant: PYR p=0.88/0.88, PV p=0.16/0.29, SOM p=0.65/0.16, VIP p=0.38/0.44, all Wilcoxon rank sum tests on n=8 mice). B: A cell-class specific reorganization of network responses to input perturbations emerged with learning. This extends the analysis of Figure 3G-I by restricting the input perturbation and readout projection to a specific pair of cell types (along the input and output linear discriminant vectors). C: Mean squared coupling of each cell type into the modes that significantly changed with learning (modes were selected based on Figure 5F, selection criteria: time constants in range 800±250 ms, low/high SNR modes defined as having normalized input SNR less/greater than 0.05). As there were multiple modes per animal, statistics were performed on a per-mode basis (mean ± sem over selected modes' mean squared coupling). Coupling is normalized such that a mean squared coupling of 1 would be obtained if all cells coupled equally into a given mode. PV neurons had lower magnitude of coupling into the high SNR modes that emerged after learning compared to the low SNR modes that disappeared with learning (p=0.04, Wilcoxon rank sum test on mean squared coupling of PV cells onto each mode, other cell types: PYR p=0.41, SOM p=0.75, VIP p=0.13). D: When the analysis of C was repeated with weights fixed across learning, there were no differences in coupling of cell types to pre-learning low SNR modes vs post-learning high SNR modes, suggesting that the differences that occur in C reflect changes in PV coupling into relevant modes with learning rather than baseline differences of coupling into low vs high SNR modes (PYR p = 0.58, PV p = 0.40, SOM p = 81, VIP p = 0.51, Wilcoxon rank sum tests).

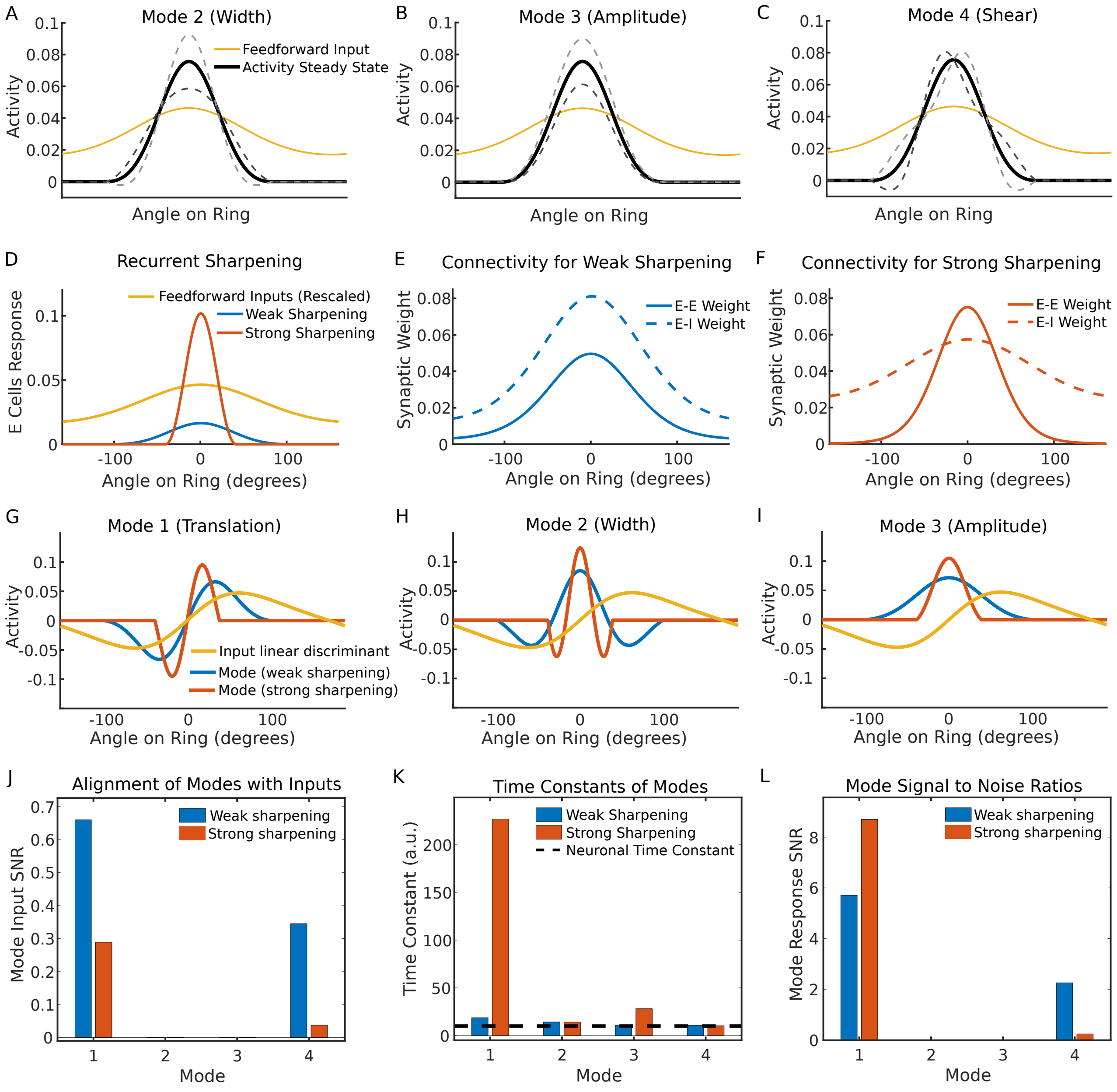

Figure S5. Uniform recurrent sharpening of sensory input reduces alignment of the slowest dynamical mode with the input linear discriminant (Related to Figure 6). To test whether connectivity changes that cause recurrent sharpening can explain the findings of the MVAR model, we examined the changes in the four slowest modes as connectivity was varied. A-C: Response steady state and perturbation along the 2nd-4th most slowly decaying modes in the E-I ring model (as in Figure 6B). D: Response of two networks to the same feedforward input, yielding weak and strong sharpening respectively. E, F: Patterns of network connectivity that induced the weak and strong sharpening of responses shown in D. Narrower E-E weights and/or broader E-I weights caused sharpening to increase. G-I: The activation patterns $\mathbf{m}$ of the three most slowly decaying modes, each overlaid with the input linear discriminant. In both networks, the translation mode was best aligned to the input discriminant and decayed most slowly. However, increased sharpening reduced the alignment of the translation mode with the input discriminant (panel G, less overlap between the red and yellow curve than between cyan and yellow). J-L: SNR of feedforward input projected onto each mode (J), the time constant for each mode (K) and the SNR of network output along each mode (L). Although the decay time constant of the translation mode increased (panel K) and generated an increase in response SNR (panel L), these improvements are nonetheless inconsistent with the unchanged time constants and increased input SNR observed over learning in the MVAR model (Figure 5A, C).

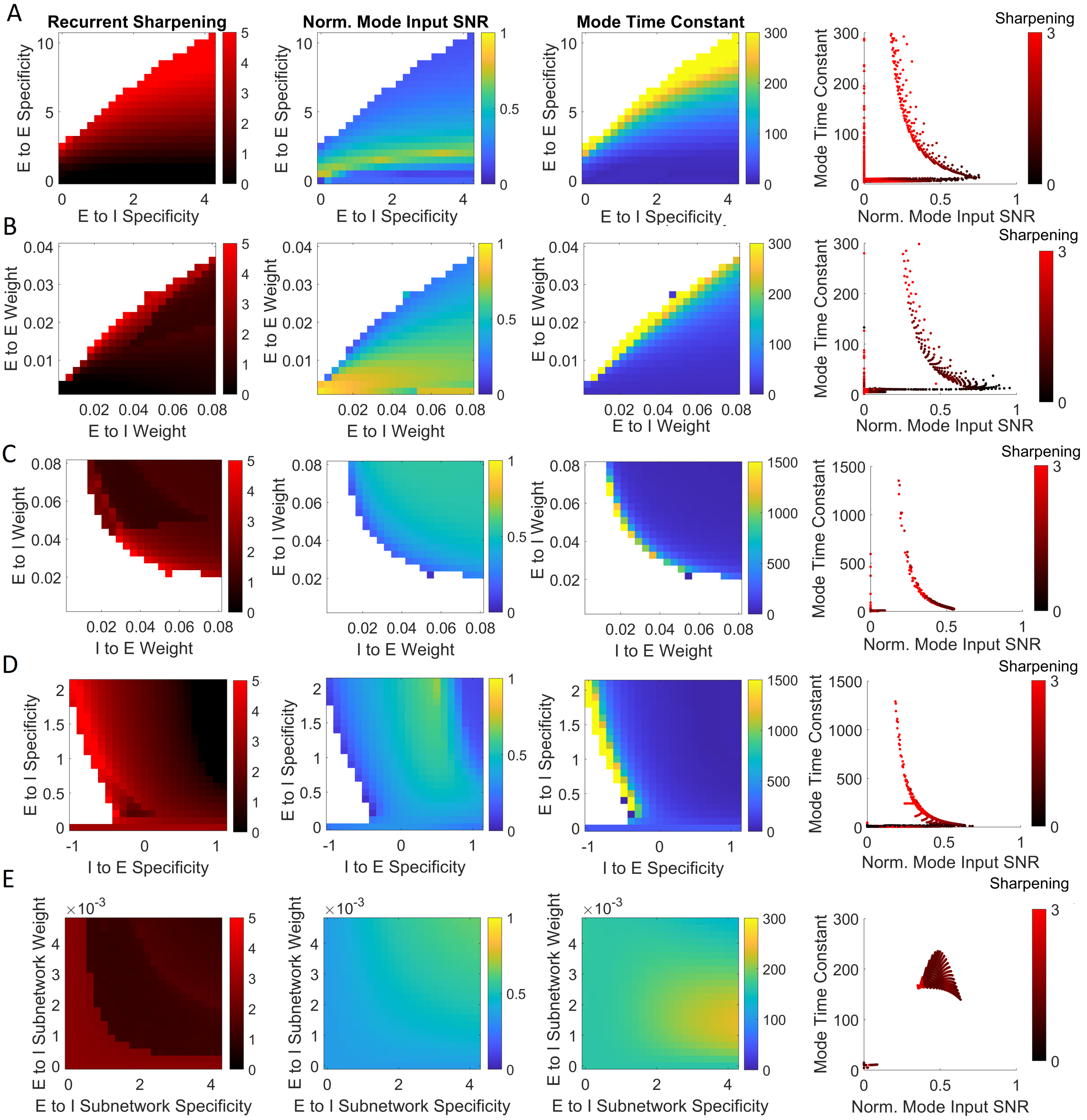

Figure S6. Parameter sweeps of excitatory and inhibitory synaptic weights (Related to Figure 6). A: Effect of varying the specificity (concentration around ring) of E to I and E to E weights. White denotes unstable networks (global instability or oscillation about an unstable fixed point). The first column shows the degree of recurrent sharpening for each network, the second column shows the normalized SNR of feedforward input projected along the best mode (the mode with greatest input SNR for a given network), the third column shows the time constant of the mode shown in the second column, the fourth column shows the time constant and input SNR of all modes pooled across all networks shown in the preceding columns of the same row. B-D: As in A but varying the magnitude of E to E and E to I weights (B), the magnitude of E to I and I to E weights (C) and the specificity of E to I and I to E weights (D). For these uniform connectivity changes (A-D), time constants and normalized input SNRs covaried across networks and were largely constrained to lie on a 1-dimensional curve (note that these curves all lie on top of one another, i.e. varying all parameters led to the same 1-dimensional curve). For modes with decay time constants significantly greater than single-neuron time constants (here, 10), increases in normalized input SNR were consistently accompanied by decreases in time constant, in contrast to the lack of change in time constants of modes with increased input SNR in the MVAR model. Although small increases in normalized in-

put SNR with fixed time constant were possible (as evidenced by horizontal scatter about the main curves in the last column), these required fine-tuning of parameters to achieve and resulted in only small improvements in SNR. E: As is A-D, but for networks with an E to I subnetwork of varying specificity and strength. These non-uniform connectivity changes yielded a fundamentally different relationship between mode time constant and input SNR, such that input SNR could be increased without altering time constants by increasing the strength and tuning of the E-I subnetwork, with a wide range of connectivity parameters achieving the desired result.

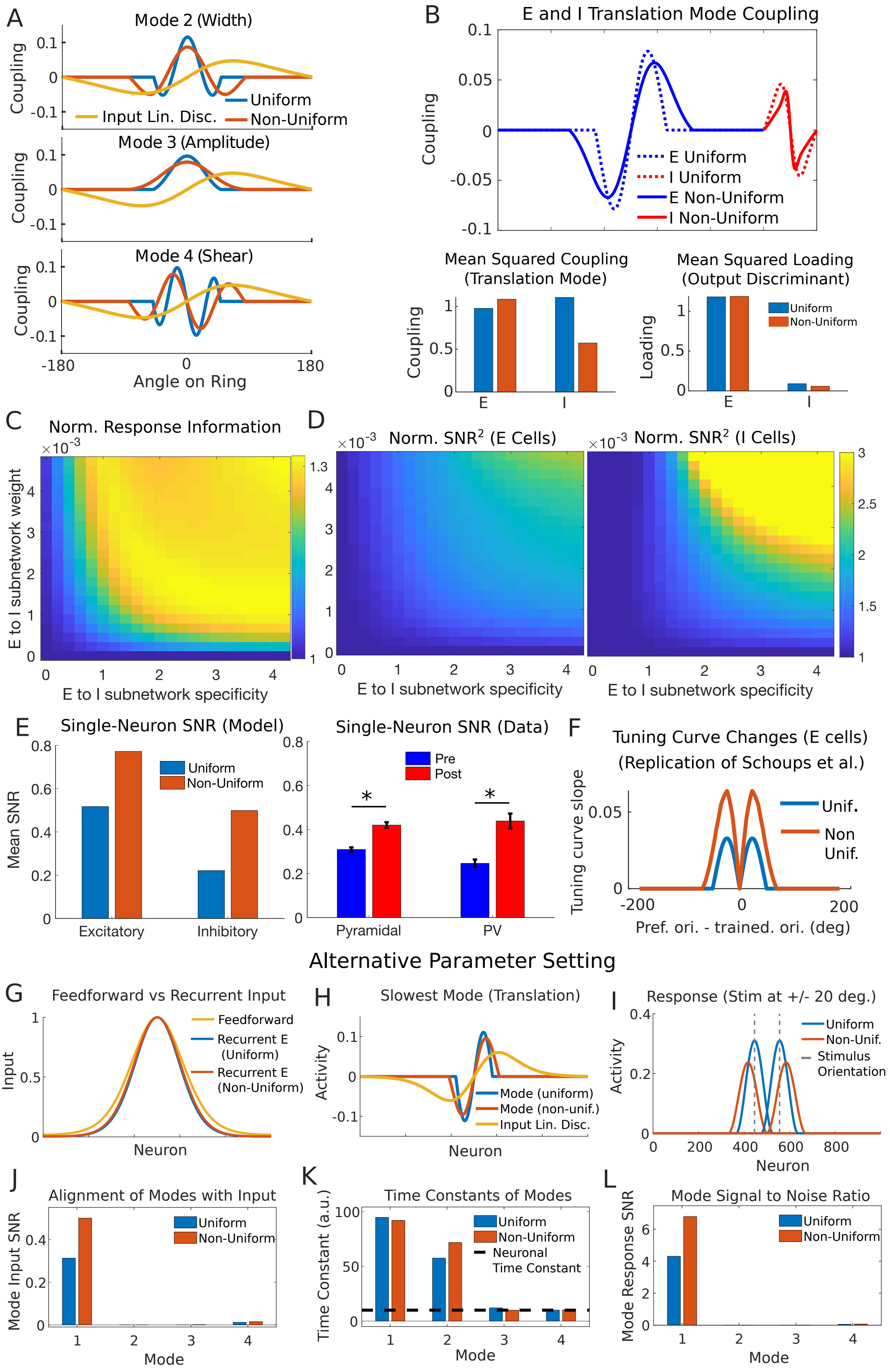

A
Mode 2 (Width)
Coupling
Uniform
Input Lin. Disc.
Non-Uniform
Mode 3 (Amplitude)
Coupling
Mode 4 (Shear)
Coupling
-180
0
180
Angle on Ring
B
E and I Translation Mode Coupling
Coupling
E Uniform
I Uniform
E Non-Uniform
I Non-Uniform
Mean Squared Coupling
(Translation Mode)
Coupling
E
I
Mean Squared Loading
(Output Discriminant)
Loading
Uniform
Non-Uniform
C
Norm. Response Information
E to I subnetwork weight
E to I subnetwork specificity
D
Norm. SNR^2 (E Cells)
Norm. SNR^2 (I Cells)
E to I subnetwork specificity
E to I subnetwork specificity
E
Single-Neuron SNR (Model)
Mean SNR
Uniform
Non-Uniform
Excitatory
Inhibitory
Single-Neuron SNR (Data)
Pre
Post
Pyramidal
PV
F
Tuning Curve Changes (E cells)
(Replication of Schoups et al.)
Tuning curve slope
Unif.
Non
Unif.
Pref. ori. - trained. ori. (deg)
Alternative Parameter Setting
G
Feedforward vs Recurrent Input
Input
Feedforward
Recurrent E
(Uniform)
Recurrent E
(Non-Uniform)
Neuron
H
Slowest Mode (Translation)
Activity
Mode (uniform)
Mode (non-unif.)
Input Lin. Disc.
Neuron
I
Response (Stim at +/- 20 deg.)
Activity
Uniform
Non-Unif.
Stimulus
Orientation
Neuron
J
Alignment of Modes with Input
Mode Input SNR
Uniform
Non-Uniform
Mode
K
Time Constants of Modes
Time Constant (a.u.)
Uniform
Non-Uniform
Neuronal
Time Constant
Mode
L
Mode Signal to Noise Ratio
Mode Response SNR
Uniform
Non-Uniform
Mode

Figure S7. Modes, response information, and stimulus tuning for networks with non-uniform connectivity (Related to Figures 6, 7). A: Activation patterns $\mathbf{m}$ for modes 2-4 in the uniform and non-uniform networks shown in Figure 6D. B: Top: Activation pattern of the translation mode in the uniform (dotted) and non-uniform (solid) networks, showing both excitatory (blue, neurons 1 to 1000) and inhibitory (red, neurons 1001 to 1200) couplings into this mode. Bottom left: The mean squared coupling of I cells was weakened in the non-uniform network, consistent with changes in PV neurons observed in the data (see Figure S4C, note also the relatively small change in magnitude of excitatory couplings due to the simultaneous broadening and drop in peak amplitude of the couplings, consistent with the lack of change of PYR cells in the data). Bottom right: The loading of E or I cells onto the response linear discriminant did not increase, as found in experimental data (Figure S4A). Note that the magnitude of loading of I cells is small because they receive untuned feedforward input. In simulations with tuned feedforward input to I cells their loading into the output discriminant was comparable to E cells and again did not increase with non-uniform inhibition. C: Linear discriminability of the two stimuli shown in Figure 7A, for networks with varying subnetwork strength and specificity (response information normalized by uniform network response information). D: Average squared SNR of excitatory and inhibitory responses (normalized by value for uniform network). Note that response information is also a squared SNR (of the linear discriminant projection). E: Left: Average SNR of excitatory and inhibitory responses for the uniform and non-uniform network (unnormalized, non-squared). Right: Average SNR of excitatory (pyramidal) and inhibitory (PV) responses for the pre- and post-learning data (called selectivity in Khan et al., 2018). F: Tuning curve changes in the model are consistent with data from monkey V1 following training on a fine-scale orientation discrimination task, which show an increased slope of tuning curves of neurons flanking the midpoint of trained orientations (Schoups et al., 2001). Following Schoups et al., the slope of the tuning curve of each neuron is plotted for the 0 degree stimulus (the “trained orientation") as a relative change in firing rate (see Methods). G-L: Alternative parameter setting with narrower feedforward input. G: Feedforward input and recurrent excitatory input for the uniform and non-uniform networks. Note that feedforward and recurrent input have similar tuning widths, as reported by Lien and Scanziani (2013). H: The input linear discriminant is broader than the recurrent dynamical modes, despite the similar width of feedforward and recurrent input. As in Figure 6E, the slowest dynamical mode overlaps more with the input discriminant in the network with non-uniform inhibition. I: Network responses to stimuli flanking the subnetwork center are repelled around the ring, leading to improved separation of stimulus responses. J-L: Alignment of the slowest mode with the input discriminant is increased in the non-uniform network (J) despite an unchanged time constant (K), leading to improved response SNR (L).

Methods S1: Supplementary Mathematical Note (Related to STAR Methods)

Contents

1 Notation **14**

2 Signal Processing Analysis **14**

3 Analysis of Fisher Information in Recurrent Networks **17**

3.1 Tuning Curve Slopes and Response Covariance 17

3.2 Relationship Between Eigenmodes and Signal Processing Analysis 19

3.3 Linear Fisher Information at Stationary State 20

3.4 Linear Fisher Information for Non-Normal Networks 22

4 The Role of Input Temporal Correlations **24**

4.1 Signal Processing Analysis with Temporal Correlations 25

4.2 Response SNR of Recurrent Networks Driven by Ornstein-Uhlenbeck Noise 26

4.3 Linear Fisher Information of Recurrent Networks Driven by Ornstein-Uhlenbeck Noise 27

1 Notation

We use bold-face lower case letters for column vectors and non-bold upper case letters for matrices. Superscript T denotes a (vector or matrix) transpose; x_i or $(\mathbf{x})_i$ denotes the ith element of vector $\mathbf{x}$; $\mathbf{x} \cdot \mathbf{y} = \mathbf{x}^T\mathbf{y} = \sum_{i=1}^{N} x_i y_i$ denotes an inner (dot) product of vectors; $\mathbf{x}\mathbf{y}^T$ denotes an outer product of vectors with (ij)th element $= x_i y_j$; $\|\mathbf{x}\| = \sqrt{\mathbf{x} \cdot \mathbf{x}}$ denotes the Euclidean vector norm; $\hat{\mathbf{x}} = \mathbf{x}/\|\mathbf{x}\|$ denotes a unit vector; $\mathrm{Tr}\,[A] = \sum_{i=1}^{N} A_{ii}$ denotes the trace of an $N \times N$ matrix A; I denotes the identity matrix; we make use of the shorthand notation for the transpose of a matrix inverse $X^{-T} = \left(X^T\right)^{-1} = \left(X^{-1}\right)^T$; $\langle \mathbf{x} \rangle$ denotes the ensemble average of $\mathbf{x}$ (or time-average for ergodic variables); δ_{ij} denotes the Kronecker delta symbol and $\delta(t)$ denotes the Dirac delta function.

2 Signal Processing Analysis

In this section we derive the results of Figure 1 in the main text. We consider a simplified model describing the sensory input to a network of neurons upon presentation a stimulus. Under the assumptions of this simple model, we derive the optimal method to discriminate a pair of stimuli based on observations of the network input. We also derive the performance of a more general class of suboptimal discrimination functions which we will later show are relevant to the way in which recurrent network dynamics act on the sensory input. This signal processing analysis places an

upper bound on the possible discrimination performance of any network receiving such sensory input, specifies the mathematical operations a network must apply to its input in order to achieve this upper bound, and shows how suboptimal integration can be understood in terms of information loss both instantaneously and over time. In the sections that follow we use the results of this analysis to interpret the behavior of recurrent networks integrating such sensory input.

We consider a network of N neurons receiving sensory input $\mathbf{u} \in \mathbb{R}^N$ generated from a stimulus s. In the scenario we consider, one of two stimuli $s \in \{s_1, s_2\}$ may be presented, each of which generates a time-series of sensory input $\mathbf{u}(s,t)$ drawn from a different distribution $p(\mathbf{u}|s)$. We assume that network input on any given trial consists of a time series $\mathbf{u}(s,t) = \mathbf{g}(s) + \boldsymbol{\eta}(t)$ with time-independent but stimulus-dependent mean $\mathbf{g}(s)$ and additive, stimulus-independent, multivariate normal noise $\boldsymbol{\eta}(t) \sim N(\mathbf{0}, \Sigma_{\boldsymbol{\eta}})$ with $\langle \boldsymbol{\eta}(t) \rangle = 0$ and $\langle \boldsymbol{\eta}(t)\boldsymbol{\eta}^T(t) \rangle = \Sigma_{\boldsymbol{\eta}}$. We wish to infer the identity of the stimulus s having observed a single realization of such a time series $\mathbf{u}$. This can be achieved optimally by maximizing the posterior probability $p(s|\mathbf{u})$ over the two stimuli.

We first consider how the two stimuli can be discriminated given an observation of network input $\mathbf{u}_0$ at a single time sample t_0. In this case, the most probable stimulus s given the input vector $\mathbf{u}_0$ can be found using linear discriminant analysis (LDA), i.e. by taking a linear projection of the input vector $\mathbf{w} \cdot \mathbf{u}_0$ and comparing this to a threshold c. To see this, note that $p(s_i|\mathbf{u}_0) = \frac{p(s_i)}{p(\mathbf{u}_0)} p(\mathbf{u}_0|s_i) = \frac{p(s_i)}{p(\mathbf{u}_0)} \left[(2\pi)^{N/2} |\Sigma_{\boldsymbol{\eta}}|^{1/2}\right]^{-1} \exp\left(-(\mathbf{u}_0 - \mathbf{g}(s_i))^T \Sigma_{\boldsymbol{\eta}}^{-1} (\mathbf{u}_0 - \mathbf{g}(s_i))\right)$, which gives $\log p(s_i|\mathbf{u}_0) = c_i - (\mathbf{u}_0 - \mathbf{g}(s_i))^T \Sigma_{\boldsymbol{\eta}}^{-1} (\mathbf{u}_0 - \mathbf{g}(s_i))$ where c_i is a constant with respect to $\mathbf{u}_0$. Thus, $\log p(s_2|\mathbf{u}_0) - \log p(s_1|\mathbf{u}_0) = c_2 - c_1 - \mathbf{g}(s_2)\Sigma_{\boldsymbol{\eta}}^{-1}\mathbf{g}(s_2) + \mathbf{g}(s_1)\Sigma_{\boldsymbol{\eta}}^{-1}\mathbf{g}(s_1) + 2(\mathbf{g}(s_2) - \mathbf{g}(s_1))^T \Sigma_{\boldsymbol{\eta}}^{-1} \mathbf{u}_0 \equiv -c + \mathbf{w}^T \mathbf{u}_0$, where we have absorbed all constant terms into a single scalar c and defined the projection vector $\mathbf{w} = 2\Sigma_{\boldsymbol{\eta}}^{-1}(\mathbf{g}(s_2) - \mathbf{g}(s_1))$. Therefore, the most probable stimulus given the observed input vector $\mathbf{u}_0$ is found by asking whether $\mathbf{w}^T \mathbf{u}_0 \lessgtr c$ (i.e., if $\mathbf{w}^T \mathbf{u}_0 > c$ then $s = s_2$ is more probable, whereas if $\mathbf{w}^T \mathbf{u}_0 < c$ then $s = s_1$ is more probable). The projection vector $\mathbf{w}$ is known as the linear discriminant, and can be understood as the vector which is normal to the hyperplane separating the two stimulus input distributions. The constant c determines the location of that hyperplane. Note that $\mathbf{w}$ and c can be rescaled by an arbitrary scalar constant without altering the decision rule.

We next consider how stimuli can best be discriminated when network input is observed sequentially in time. When statistically independent inputs $\mathbf{u}(t)$ are observed at a set of times $t \in \mathcal{T}$ (a continuous interval or discrete samples), the optimal solution is to perform a time-averaged LDA using the decision rule $\mathbf{w} \cdot \langle \mathbf{u}(t) \rangle_{t \in \mathcal{T}} \lessgtr c$. Here, $\langle \cdot \rangle_{t \in \mathcal{T}}$ is the sample mean over the set of time samples and $\mathbf{w}$, c are the same quantities as in the single time sample case. This result follows directly from the single time sample case and the fact that $\log p(s_i|\mathbf{u}(t), t \in \mathcal{T}) = \sum_{t \in \mathcal{T}} \log p(s_i|\mathbf{u}(t))$ for statistically independent samples.

An intuitive way to understand this time-averaged LDA solution is to search for the linear projection $\mathbf{n} \in \mathbb{R}^N$ and temporal filter $f(t)$ which, when applied jointly to the input time series $\mathbf{u}(s,t)$, generate the scalar output with the greatest signal to noise ratio with respect to the two stimuli to be discriminated. In the case of a continuous time series of length T, i.e. $t \in [0, T]$, we denote the scalar output of such an operation as $d_{\mathbf{n},f}(s,T) = \int_0^T f(\tau)(\mathbf{n} \cdot \mathbf{u}(s, T - \tau)) d\tau$. The signal to noise ratio of $d_{\mathbf{n},f}(s,T)$ is defined as:

$$\mathrm{SNR}_T^2(\mathbf{n}, f) = \frac{\left[\langle d_{\mathbf{n},f}(s,T) \rangle_{s=s_2} - \langle d_{\mathbf{n},f}(s,T) \rangle_{s=s_1}\right]^2}{\frac{1}{2}\left[\mathrm{Var}\left[d_{\mathbf{n},f}(s,T)\right]_{s=s_1} + \mathrm{Var}\left[d_{\mathbf{n},f}(s,T)\right]_{s=s_2}\right]}. \tag{1}$$

Provided that $\mathbf{u}(s,t)$ has Gaussian statistics, $d_{\mathbf{n},f}(s,T)$ is a normally distributed random variable under each stimulus s. Moreover, assuming stimulus-independent input covariance, the variance of $d_{\mathbf{n},f}(s,T)$ is independent of s. As a consequence, the above signal to noise ratio is sufficient to determine stimulus discrimination performance of an optimal observer receiving the scalar output $d_{\mathbf{n},f}(s,T)$ (in particular, $p(\mathrm{correct}) = \Phi(\mathrm{SNR}_T/2)$ where Φ is the cumulative function of the stan-

dard normal distribution). The solution derived above by maximizing the posterior probability over s corresponds to setting $f(t) = 1/T$, $\mathbf{n} = \mathbf{w} = 2\Sigma_{\boldsymbol{\eta}}^{-1}(\mathbf{g}(s_2) - \mathbf{g}(s_1))$. We rederive this optimal solution below through maximization of the above SNR. As we will show, using a different projection vector $\mathbf{n}$ or temporal filter f reduces the signal to noise ratio (except for scaling of f or $\mathbf{n}$, which has no effect). Thus, the linear discriminant vector $\mathbf{w}$ can also be understood as the vector which maximizes the signal to noise ratio of the projected input.

We now derive the optimal choice of $\mathbf{n}, f$ and quantify the performance of both optimal and sub-optimal choices under the assumption of temporally uncorrelated Gaussian input noise. In this case, the influence of $\mathbf{n}$ and f on the signal to noise ratio of the scalar output $d_{\mathbf{n},f}(s,T)$ takes on a particularly simple form. In particular, we then have $\langle \boldsymbol{\eta}(t)\boldsymbol{\eta}^T(t')\rangle = \Sigma_{\boldsymbol{\eta}}\delta(t-t')$, so that $\langle d_{\mathbf{n},f}(s,T)\rangle_{s=s_i} = \mathbf{n}\cdot\mathbf{g}(s_i)\int_0^T f(\tau)d\tau$ and $\mathrm{Var}\left[d_{\mathbf{n},f}(s,T)\right]_{s=s_i} = \mathbf{n}\cdot\Sigma_{\boldsymbol{\eta}}\mathbf{n}\left[\int_0^T f^2(\tau)d\tau\right]$. Defining $\Delta\mathbf{g} = \mathbf{g}(s_2) - \mathbf{g}(s_1)$, the output signal to noise ratio is then given by:

$$\mathrm{SNR}_T^2(\mathbf{n}, f) = \frac{[\mathbf{n}\cdot\Delta\mathbf{g}]^2}{\mathbf{n}\cdot\Sigma_{\boldsymbol{\eta}}\mathbf{n}}\frac{\left[\int_0^T f(\tau)d\tau\right]^2}{\int_0^T f^2(\tau)d\tau} \equiv \mathrm{SNR}_{\mathrm{input}}^2(\mathbf{n})I_T(f) \tag{2}$$

where $\mathrm{SNR}_{\mathrm{input}}^2(\mathbf{n}) = [\mathbf{n}\cdot\Delta\mathbf{g}]^2 / [\mathbf{n}\cdot\Sigma_{\boldsymbol{\eta}}\mathbf{n}]$ is the signal to noise ratio of the instantaneous input projected along $\mathbf{n}$ and $I_T(f) = \left[\int_0^T f(\tau)d\tau\right]^2 / \left[\int_0^T f^2(\tau)d\tau\right]$ is a temporal integration factor. Thus, the total signal to noise ratio factors into an instantaneous term and a temporal term. We can therefore proceed to maximize each of these two factors in turn with respect to $\mathbf{n}$ and f respectively. To do so, we apply the Cauchy-Schwarz inequality to derive two inequalities, $\mathrm{SNR}_{\mathrm{input}}^2(\mathbf{n}) \leq \Delta\mathbf{g}\cdot\Sigma_{\boldsymbol{\eta}}^{-1}\Delta\mathbf{g}$ and $I_T(f) \leq T$. To see how the first inequality arises, note that $\mathbf{n}\cdot\Sigma_{\boldsymbol{\eta}}\mathbf{n} = \left(\Sigma_{\boldsymbol{\eta}}^{1/2}\mathbf{n}\right)\cdot\left(\Sigma_{\boldsymbol{\eta}}^{1/2}\mathbf{n}\right)$, while by Cauchy-Schwarz $|\mathbf{n}\cdot\Delta\mathbf{g}(s)| = |\left(\Sigma_{\boldsymbol{\eta}}^{1/2}\mathbf{n}\right)\cdot\left(\Sigma_{\boldsymbol{\eta}}^{-1/2}\Delta\mathbf{g}\right)| \leq \sqrt{\left(\Sigma_{\boldsymbol{\eta}}^{1/2}\mathbf{n}\right)\cdot\left(\Sigma_{\boldsymbol{\eta}}^{1/2}\mathbf{n}\right)}\sqrt{\left(\Sigma_{\boldsymbol{\eta}}^{-1/2}\Delta\mathbf{g}\right)\cdot\left(\Sigma_{\boldsymbol{\eta}}^{-1/2}\Delta\mathbf{g}\right)}$. Inserting these into the definition of $\mathrm{SNR}_{\mathrm{input}}^2(\mathbf{n})$ and cancelling terms in the numerator and denominator gives the desired inequality. The second inequality follows in a similar fashion: the integral Cauchy-Schwarz inequality gives $|\int_0^T f(\tau)d\tau| = |\int_0^T f(\tau)\cdot 1 d\tau| \leq \sqrt{\int_0^T f^2(\tau)d\tau}\sqrt{\int_0^T 1^2 d\tau} = \sqrt{\int_0^T f^2(\tau)d\tau}\sqrt{T}$ which can be inserted into the definition of $I_T(f)$ to arrive at the desired result. It can easily be verified that these upper bounds are achieved when $f(t) = \alpha$ and $\mathbf{n} = \beta\Sigma_{\eta}^{-1}\Delta\mathbf{g} = \beta\mathbf{w}$ for any pair of constants α, β. Thus, we have arrived at the same optimal solution for stimulus discrimination using two different methods: first, by maximizing the posterior probability of the stimulus given the observed network input; second, by maximizing the signal to noise ratio obtained by linear projection and temporal filtering of the network input.

Several conclusions can be drawn from this analysis. First, for invertible $\Sigma_{\boldsymbol{\eta}}$, the information available to a decoder of network input over a time window T is finite and the sources of information loss can be factored into an instantaneous term $\mathrm{SNR}_{\mathrm{input}}$ and a temporal term $I_T(f)$ (note that further sources of information loss may occur when different functions than those considered here are applied to the network input, as we will see when we study recurrent networks). Moreover, even in the limit of infinite time, the information available to decoder with finite timescales of temporal integration remains finite due to the loss of previously integrated information over time (i.e., if $\lim_{T\to\infty} I_T(f) < \infty$). As we have shown, the optimal solution for discriminating pairs of stimuli given an observed time series of network input is to project that network input onto the direction carrying the most information instantaneously, and then to integrate that projection using a sufficiently long time constant in order to avoid loss of previously integrated information (i.e., using a choice of f such that $I_T(f)/T \approx 1$). In the following analysis of information transmission through recurrent networks, we will focus on the information contained in the output of networks with finite dynamical time constants following integration of sensory input over a long period of time.

3 Analysis of Fisher Information in Recurrent Networks

We next quantify the capacity of an optimal observer to discriminate stimuli based on observations of the output of a recurrent network which receives the sensory input described in the previous section. We analyze the transformation of noisy sensory input by a recurrent network of N nonlinear units governed by the following dynamics:

$$\tau_i \frac{\partial r_i}{\partial t} = -r_i + \phi_i \left(\sum_j W_{ij} r_j + u_i(s,t) \right) \tag{3}$$

where r_i represents the firing rate of neuron i, τ_i is its time constant, ϕ_i is its input-output nonlinearity (or transfer function), W_{ij} is the synaptic weight from neuron j to neuron i and $u_i(s,t) = g_i(s) + \eta_i(t)$ is the feedforward input to neuron i at time t given a sensory stimulus s. As before, inputs are defined as having additive, multivariate Gaussian, temporally uncorrelated, stimulus-independent noise $\boldsymbol{\eta}(t)$.

Rather than deriving the signal to noise ratio for two discrete stimuli as above, we will derive the Fisher Information of network responses $\mathbf{r}$ with respect to a continuous one-dimensional stimulus s. The Fisher Information places a lower bound on the variance of any unbiased estimator of s from $\mathbf{r}$. For responses following a multivariate normal distribution, the Fisher Information is given by $\mathcal{I}_F^{\text{tot}} = \mathbf{r}'^T \Sigma^{-1} \mathbf{r}' + \frac{1}{2} \text{Tr} \left[\left(\Sigma^{-1} \Sigma' \right)^2 \right]$, where $r_i' \equiv \frac{\partial \langle r_i \rangle}{\partial s}$ is the slope of the tuning curves with respect to s, $\Sigma = \langle (\mathbf{r} - \langle \mathbf{r} \rangle) (\mathbf{r} - \langle \mathbf{r} \rangle)^T \rangle$ is the covariance of network responses under that stimulus and $\Sigma' = \frac{\partial \Sigma}{\partial s}$ is the change in response covariance as the stimulus is changed. When Σ is stimulus-dependent, achieving the precision of stimulus discrimination set by the Fisher Information requires a quadratic decoder of neural activity (Shamir and Sompolinsky, 2004; Yang et al., 2021). We focus instead on the linear Fisher Information $\mathcal{I}_F = \mathbf{r}'^T \Sigma^{-1} \mathbf{r}'$ following previous studies (Seriès et al., 2004; Beck et al., 2011; Moreno-Bote et al., 2014). In addition to being analytically tractable, the linear Fisher Information has several theoretical advantages. First, even for networks in which the optimal decoder is quadratic (or otherwise nonlinear), the linear Fisher Information describes the optimal local linear decoder of small changes in the stimulus based on network responses (Seriès et al., 2004; Beck et al., 2011; Kafashan et al., 2021). Second, the linear Fisher Information places a bound on the precision of an optimal linear estimator even for non-Gaussian response distributions, whereas the quadratic term holds only for Gaussian statistics (Yang et al., 2021; Kafashan et al., 2021). Third, the linear Fisher Information has a natural relationship to linear discriminant analysis, in particular $\mathcal{I}_F \Delta s^2 \approx \Delta \mathbf{r}^T \Sigma^{-1} \Delta \mathbf{r}$ for sufficiently small Δs, which allows us to relate our findings back to the two-stimulus discrimination task studied experimentally in the main text and above in our signal processing analysis. Fourth, the linear Fisher Information can be understood as a signal to noise ratio, much as in our above signal processing analysis. In particular, the linear Fisher Information is the SNR of $\mathbf{w}^T \mathbf{r}$, where $\mathbf{w} = \Sigma^{-1} \mathbf{r}'$ is the linear discriminant vector for discriminating infinitesimal changes in s based on network output $\mathbf{r}$.

In order to evaluate the linear Fisher Information of the output of a recurrent network, we next derive expressions for the tuning curve derivatives $\mathbf{r}'$ and response covariance Σ for networks obeying the dynamics of Equation (3) and driven to stationary state.

3.1 Tuning Curve Slopes and Response Covariance

The linear Fisher Information of the output of a recurrent network $\mathbf{r}$ depends on two quantities: the tuning curves with respect to the stimulus $\mathbf{r}' = \frac{\partial \langle \mathbf{r} \rangle}{\partial s}$, and the response covariance $\Sigma = \langle (\mathbf{r} - \langle \mathbf{r} \rangle)(\mathbf{r} - \langle \mathbf{r} \rangle)^T \rangle$. To derive expressions for these, we will rely on two approximations: first, we linearize the system about a stimulus-evoked fixed point; second, we compute the statistics of the stationary state

response of the linearized system.

To estimate the tuning curve derivatives $\mathbf{r}' = \frac{\partial \langle \mathbf{r} \rangle}{\partial s}$, we differentiate the noise-free fixed points of the network with respect to the stimulus. To do so we set $\frac{\partial \mathbf{r}}{\partial t} = 0$ and $\boldsymbol{\eta} = 0$ and then differentiate both sides of Equation (3) with respect to s. On performing this calculation, we obtain $\mathbf{r}'_{SS}(s) = -J^{-1}(s)\Phi'(s)\mathbf{g}'(s)$, where $\mathbf{r}_{SS}(s) = \phi\left(W\mathbf{r}_{SS}(s) + \mathbf{g}(s)\right)$ is the noise-free steady state response, $J(s) = \Phi'(s)W - T^{-1}$ is the Jacobian, i.e. a matrix of effective interaction weights with $T_{ij} = \tau_j \delta_{ij}$ and $\Phi'_{ij}(s) = \delta_{ij}\tau_j^{-1}\frac{d\phi_j(x)}{dx}|_{x=\sum_k W_{jk} r_k(s) + g_j(s)}$ is a diagonal matrix quantifying the sensitivity of each neuron to small changes in its input (both feedforward and recurrent). Note that this result involves an approximation: we have replaced the average stationary state response of the stochastic system $\langle \mathbf{r} \rangle$ with the fixed point of the noise-free system $\mathbf{r}_{SS}$. The accuracy of this approximation depends on the nonlinearity near the fixed point and on the magnitude of the noise. Note that while we did not explicitly linearize in order to obtain this solution, an identical result is obtained by first linearizing the network dynamics about the noise-free fixed point, computing the mean response of the noise-injected linearized system at stationary state, and then differentiating this with respect to the stimulus. This is the approach we next take in order to obtain an approximation for the response covariance.

To derive the response covariance within the linearized stationary state approximation, we first linearize Equation (3) about the fixed point $\mathbf{r} = \mathbf{r}_{SS}(s)$ by applying a first order Taylor expansion for small fluctuations $\delta\mathbf{r}$ about the fixed point $\mathbf{r}_{SS}$, i.e. $\mathbf{r} = \mathbf{r}_{SS} + \delta\mathbf{r}$ with $\|\delta\mathbf{r}\| \approx 0$. This gives the following approximation to the dynamics:

$$\frac{\partial \mathbf{r}}{\partial t} \approx J\left(\mathbf{r} - \mathbf{r}_{SS}\right) + \Phi'\boldsymbol{\eta} \tag{4}$$

where J and Φ' are as defined above. Equation (4) describes a multivariate Ornstein-Uhlenbeck process, and has the general solution:

$$\mathbf{r}(t) - \mathbf{r}_{SS} = e^{J(t-t_0)}\left(\mathbf{r}(t_0) - \mathbf{r}_{SS}\right) + \int_{t_0}^{t} e^{J(t-\tau)}\Phi'\boldsymbol{\eta}(\tau)d\tau \tag{5}$$

for any initial condition $\mathbf{r}(t_0)$, where e^X is the matrix exponential function. Provided the fixed point is stable (i.e., all eigenvalues of J have negative real part) we can take the stationary state limit by letting $t_0 \to -\infty$ to obtain:

$$\mathbf{r} - \mathbf{r}_{SS} = \int_{-\infty}^{t} e^{J(t-\tau)}\Phi'\boldsymbol{\eta}(\tau)d\tau. \tag{6}$$

Assuming that input noise is temporally uncorrelated, i.e. $\langle \boldsymbol{\eta}(t)\boldsymbol{\eta}^T(t') \rangle = \Sigma_{\boldsymbol{\eta}}\delta(t-t')$, the stationary-

state response covariance $\Sigma_{SS} = \langle (\mathbf{r} - \mathbf{r}_{SS}) (\mathbf{r} - \mathbf{r}_{SS})^T \rangle$ is:

$$\Sigma_{SS} = \int_{-\infty}^{t} \int_{-\infty}^{t} e^{J(t-\tau)} \Phi' \langle \boldsymbol{\eta}(\tau) \boldsymbol{\eta}^T(\tau') \rangle \Phi' e^{J^T(t-\tau')} d\tau d\tau' \quad (7)$$

$$= \int_{-\infty}^{t} \int_{-\infty}^{t} e^{J(t-\tau)} \Phi' \Sigma_{\boldsymbol{\eta}} \delta(\tau - \tau') \Phi' e^{J^T(t-\tau')} d\tau d\tau' \quad (8)$$

$$= \int_{-\infty}^{t} e^{J(t-\tau)} \Phi' \Sigma_{\boldsymbol{\eta}} \Phi' e^{J^T(t-\tau)} d\tau \quad (9)$$

$$= \int_{-\infty}^{t} \left[\sum_i \mathbf{v}_i^R \left(\mathbf{v}_i^L\right)^T e^{\lambda_i (t-\tau)} \right] \Phi' \Sigma_{\boldsymbol{\eta}} \Phi' \left[\sum_j \mathbf{v}_j^R \left(\mathbf{v}_j^L\right)^T e^{\lambda_j (t-\tau)} \right]^T d\tau \quad (10)$$

$$= \sum_{i,j} \mathbf{v}_i^R \left(\mathbf{v}_i^L\right)^T \Phi' \Sigma_{\boldsymbol{\eta}} \Phi' \mathbf{v}_j^L \left(\mathbf{v}_j^R\right)^T \int_{-\infty}^{t} e^{(\lambda_i + \lambda_j)(t-\tau)} d\tau \quad (11)$$

$$= -\sum_{i,j} \mathbf{v}_i^R \left(\mathbf{v}_i^L\right)^T \Phi' \Sigma_{\boldsymbol{\eta}} \Phi' \mathbf{v}_j^L \left(\mathbf{v}_j^R\right)^T \frac{1}{\lambda_i + \lambda_j} \quad (12)$$

where we have made use of the eigendecomposition of the Jacobian $J = V \Lambda V^{-1} = \sum_{i=1}^{N} \mathbf{v}_i^R \left(\mathbf{v}_i^L\right)^T \lambda_i$ and of its matrix exponential $e^{J\tau} = V e^{\Lambda \tau} V^{-1} = \sum_{i=1}^{N} \mathbf{v}_i^R \left(\mathbf{v}_i^L\right)^T e^{\lambda_i \tau}$. We use superscripts L and R to denote left and right eigenvectors, which are the rows of V^{-1} and columns of V respectively. Note that the left and right eigenvectors do not in general form orthonormal bases, but do satisfy the orthogonality relations $\mathbf{v}_i^L \cdot \mathbf{v}_j^R = \delta_{ij}$. This orthogonality relation does not typically allow for both left and right eigenvectors to have unit length, because $\mathbf{v}_i^L \cdot \mathbf{v}_i^R = \|\mathbf{v}_i^L\| \|\mathbf{v}_i^R\| \cos\theta = 1$. Where a choice of normalization is required, we choose to normalize left eigenvectors to unit length, in which case right eigenvectors typically do not have unit length. This convention for normalization is entirely arbitrary and is made for convenience only, reflecting the central role that left eigenvectors play in our theory. In the main text, we refer to the left eigenvectors as the mode activation patterns $\mathbf{m}$, and we define their time constants as $\tau = -1/\mathrm{Re}(\lambda)$. Note that the stationary state covariance also satisfies the Lyapunov equation $J\Sigma_{SS} + \Sigma_{SS} J^T + \Phi' \Sigma_{\boldsymbol{\eta}} \Phi' = 0$, which is well known in the control theory literature. This Lyapunov equation can be solved efficiently using numerical methods, but is less convenient when deriving the analytical results we present the following sections.

3.2 Relationship Between Eigenmodes and Signal Processing Analysis

With the results of the previous section in hand, we are now in a position to formulate a general expression for the Linear Fisher Information of the network response. Before doing so, however, we first show that the signal to noise ratio of the network output projected along any left eigenvector (i.e., mode) $\mathbf{v}_i^L$ of the Jacobian J takes on a particularly simple form that is readily interpretable using the insights obtained from our earlier signal processing analysis. The linear Fisher Information can be understood as the signal to noise ratio of network output projected onto the linear discriminant vector for the network output, which in turn can be understood as the projection vector which maximizes this signal to noise ratio (as shown in our signal processing analysis). Thus, deriving an expression for the signal to noise ratio along any other projection (in this case, a left eigenvector) allows us to place a lower bound on the total information in the network response. The equations derived in this section form the basis for the results presented in Figure 2 of the main text, and motivate much of our analysis of the experimental data and network models presented in Figures 3-7.

To simplify the expressions which follow, we first make a change of variables $\tilde{\mathbf{r}} \equiv \Phi'^{-1} \mathbf{r}$ and $\tilde{J} \equiv \Phi'^{-1} J \Phi' = W\Phi' - T^{-1}$. In this basis, Equation (4) becomes $\dot{\tilde{\mathbf{r}}} = \tilde{J} (\tilde{\mathbf{r}} - \tilde{\mathbf{r}}_{SS}) + \boldsymbol{\eta}$, while $\tilde{J}$ has eigenvalues $\tilde{\lambda}_i = \lambda_i$ and eigenvectors $\tilde{\mathbf{v}}_i^L = \Phi' \mathbf{v}_i^L$, $\tilde{\mathbf{v}}_i^R = \Phi'^{-1} \mathbf{v}_i^R$. We can express the tuning curve

derivatives as $\mathbf{r}'_{SS} = -\sum_i \frac{1}{\lambda_i}\mathbf{v}_i^R \left(\mathbf{v}_i^L\right)^T \Phi'\mathbf{g}'$. Then using the identity $\mathbf{v}_i^L \cdot \mathbf{v}_j^R = \delta_{ij}$, both Σ_{SS} and $\mathbf{r}'_{SS}$ can be expressed in terms of their projections onto left eigenvectors, which obtains:

$$\left(\mathbf{v}_i^L\right)^T \mathbf{r}'_{SS} = -\left(\mathbf{v}_i^L\right)^T \Phi'\mathbf{g}'\frac{1}{\lambda_i} = -\left(\tilde{\mathbf{v}}_i^L\right)^T \mathbf{g}'\frac{1}{\lambda_i}, \tag{13}$$

$$\left(\mathbf{v}_i^L\right)^T \Sigma_{SS}\mathbf{v}_j^L = -\left(\mathbf{v}_i^L\right)^T \Phi'\Sigma_{\boldsymbol{\eta}}\Phi'\mathbf{v}_j^L\frac{1}{\lambda_i+\lambda_j} = -\left(\tilde{\mathbf{v}}_i^L\right)^T \Sigma_{\boldsymbol{\eta}}\tilde{\mathbf{v}}_j^L\frac{1}{\lambda_i+\lambda_j}. \tag{14}$$

We can then calculate the signal to noise ratio of the instantaneous network response at stationary state, projected along any left eigenvector $\mathbf{v}_i^L$:

$$\mathrm{SNR}^2_{\mathrm{output}}\left(\mathbf{v}_i^L\right) \equiv \frac{\left(\mathbf{v}_i^L\cdot\mathbf{r}'_{SS}\right)^2}{(\mathbf{v}_i^L)^T\Sigma_{SS}\mathbf{v}_i^L} = -\frac{\left(\tilde{\mathbf{v}}_i^L\cdot\mathbf{g}'\right)^2}{\left(\tilde{\mathbf{v}}_i^L\right)^T\Sigma_{\boldsymbol{\eta}}\tilde{\mathbf{v}}_i^L}\frac{2}{\lambda_i} = \mathrm{SNR}^2_{\mathrm{input}}\left(\tilde{\mathbf{v}}_i^L\right)2\tau_i \tag{15}$$

where we have defined $\tau_i = -1/\lambda_i$, under the assumption that $\lambda_i \in \mathbb{R}$ (i.e., the mode is not oscillatory).

Equation (15) demonstrates that the SNR of network output following projection onto any left eigenvector of J is equal to the SNR of network input projected along the corresponding left eigenvector of $\tilde{J}$, multiplied by the decay time constant of that eigenmode and by a constant factor of 2. This result is identical to that obtained in our signal processing analysis, and can easily be derived from Equation (2) by setting $f(t) = e^{-t/\tau_i}$, $\mathbf{n} = \tilde{\mathbf{v}}_i^L$, and taking $T \to \infty$. The reason for this correspondence is that left eigenvectors implement exactly the linear projection and temporal filtering operations required for optimal stimulus discrimination, up to the minor caveat that the optimal (but biologically implausible) $f(t) = 1/T$ is replaced with an exponential filter $f(t) = e^{-t/\tau_i}$. We can identify the scalar output $d_{\mathbf{n},f}(s,T)$ from the signal processing analysis with the linear projection of the network response $\mathbf{v}_i^L \cdot \mathbf{r}$. Equation (15) is the main result presented in Figure 2, where we considered a purely linear (rather than linearized) system, which slightly simplifies the result because $\tilde{\mathbf{v}}_i^L = \mathbf{v}_i^L$.

It is important to emphasize that, while Equation (15) can be understood as a special case of our more general signal processing analysis (which allows for arbitrary filters $f(t)$), this result in fact relies on the unique properties of left eigenvectors. For example, a similar result is not obtained when projecting responses along right eigenvectors $\mathbf{v}_i^R$. Indeed, there is a deeper reason that left eigenvectors exhibit this property. This result relies on two facts: first, network input along each left eigenvector is mapped onto network output along the corresponding right eigenvector; second, left eigenvectors are orthogonal to right eigenvectors ($\mathbf{v}_i^L \cdot \mathbf{v}_j^R = \delta_{ij}$). Together, these properties ensure that the network dynamics decouple into independent leaky integrators when projected onto left eigenvectors, in particular $\tilde{\mathbf{v}}_i^L \cdot \dot{\tilde{\mathbf{r}}} = \lambda_i\tilde{\mathbf{v}}_i^L \cdot (\tilde{\mathbf{r}} - \tilde{\mathbf{r}}_{SS}) + \tilde{\mathbf{v}}_i^L \cdot \boldsymbol{\eta}$ (and also $\mathbf{v}_i^L \cdot \dot{\mathbf{r}} = \lambda_i\mathbf{v}_i^L \cdot (\mathbf{r} - \mathbf{r}_{SS}) + \mathbf{v}_i^L \cdot \Phi'\boldsymbol{\eta}$). This decoupling into independent processes is a unique feature of left eigenvectors, and motivates the use of the word “modes" to describe them. This observation underscores an additional source of information loss in recurrent networks that was not apparent from our signal processing analysis - because recurrent networks map multiple different projections of their input onto any given projection of their output, they superimpose both relevant information and additional irrelevant noise within the same output projection, which reduces the signal to noise ratio. Left eigenvectors avoid this source of information loss by isolating a single projection of network input and preserving it along a single projection of the network output, allowing them to integrate input information optimally.

3.3 Linear Fisher Information at Stationary State

We now return to the problem of estimating the linear Fisher Information of the network response. The Linear Fisher Information is equal to the (squared) signal to noise ratio obtained after projecting

network responses along their linear discriminant $\mathbf{w} = \Sigma_{SS}^{-1}\mathbf{r}'_{SS}$. Because the linear discriminant is the projection which maximizes this signal to noise ratio, the linear Fisher Information will typically exceed the signal to noise ratio obtained following projection along any left eigenvector (Equation (15)). Inserting the expressions for tuning curve slopes and response covariance derived above into the equation for the linear Fisher Information, we obtain:

$$\mathcal{I}_F \equiv \mathbf{r}'_{SS} \cdot \Sigma_{SS}^{-1}\mathbf{r}'_{SS} = -\mathbf{g}'^T \left[\Phi'^{-1} \sum_{i,j} \mathbf{v}_i^R \left(\mathbf{v}_i^L\right)^T \Phi' \Sigma_\eta \Phi' \mathbf{v}_j^L \left(\mathbf{v}_j^R\right)^T \Phi'^{-1} \frac{\lambda_i \lambda_j}{\lambda_i + \lambda_j} \right]^{-1} \mathbf{g}'. \tag{16}$$

Using again the change of basis introduced in the previous section, this result simplifies to:

$$\mathcal{I}_F = \mathbf{g}'^T \left[\sum_{i,j} \tilde{\mathbf{v}}_i^R \left(\tilde{\mathbf{v}}_j^R\right)^T \Gamma_{ij} \right]^{-1} \mathbf{g}' \equiv \mathbf{g}'^T \Sigma_{\text{eff}}^{-1} \mathbf{g}', \quad \Gamma_{ij} = -\left(\tilde{\mathbf{v}}_i^L\right)^T \Sigma_{\boldsymbol{\eta}} \tilde{\mathbf{v}}_j^L \frac{\lambda_i \lambda_j}{\lambda_i + \lambda_j} = \left(\tilde{V}^{-1} \Sigma_{\boldsymbol{\eta}} \tilde{V}^{-T}\right)_{ij} \frac{1}{\tau_i + \tau_j}. \tag{17}$$

This equation provides intuition as to how the transformation of sensory input through the recurrent network shapes the information about the stimulus available in the network output. The linear Fisher Information of the instantaneous sensory input is $\mathbf{g}'^T \Sigma_{\boldsymbol{\eta}}^{-1} \mathbf{g}'$, so that Σ_{eff} encapsulates the relationship between input and output information (the transformation of both input signal and noise by the network have been absorbed into this effective covariance). The coefficients Γ_{ij} have a natural interpretation as the effective covariance between network responses projected onto pairs of left eigenvectors, i.e. $\Gamma_{ij} = \left(\tilde{\mathbf{v}}_i^L\right)^T \Sigma_{\text{eff}} \tilde{\mathbf{v}}_j^L$ and $\Gamma = \tilde{V}^{-1} \Sigma_{\text{eff}} \tilde{V}^{-T}$. Moreover, these coefficients depend on the alignment of the corresponding pair of left eigenvectors with the sensory input covariance and also depend inversely on the timescale of integration along those eigenvectors $\tau_i + \tau_j = -\left(\lambda_i + \lambda_j\right) / \left(\lambda_i \lambda_j\right)$ (assuming the eigenvalues are real). Moreover, Γ is the solution to the Lyapunov equation $\Gamma \Lambda^{-1} + \Lambda^{-1} \Gamma + \tilde{V}^{-1} \Sigma_\eta \tilde{V}^{-T} = 0$, meaning it is the stationary state covariance of a system with injected covariance $\tilde{V}^{-1} \Sigma_\eta \tilde{V}^{-T}$ and dynamical evolution Λ^{-1}. Similarly, the effective covariance follows the Lyapunov equation $\tilde{J}^{-1} \Sigma_{\text{eff}} + \Sigma_{\text{eff}} \tilde{J}^{-T} + \Sigma_{\boldsymbol{\eta}} = 0$.

The Fisher Information can be expressed compactly in matrix form as:

$$\mathcal{I}_F = \mathbf{g}'^T \tilde{V}^{-T} \Gamma^{-1} \tilde{V}^{-1} \mathbf{g}' = \sum_{ij} \left(\mathbf{g}' \cdot \tilde{\mathbf{v}}_i^L\right) \left(\mathbf{g}' \cdot \tilde{\mathbf{v}}_j^L\right) \left(\Gamma^{-1}\right)_{ij}. \tag{18}$$

Unfortunately, this expression for Fisher Information is difficult to compute analytically except in certain special cases where Γ can be directly inverted, such as when Γ is a 2x2 matrix or a diagonal matrix. For a diagonal Γ we have:

$$\mathcal{I}_F = -\sum_i \frac{\left(\mathbf{g}' \cdot \tilde{\mathbf{v}}_i^L\right)^2}{\left(\tilde{\mathbf{v}}_i^L\right)^T \Sigma_\eta \tilde{\mathbf{v}}_i^L} \frac{2}{\lambda_i} = \sum_i \text{SNR}_{\text{input}}^2 \left(\tilde{\mathbf{v}}_i^L\right) 2\tau_i \tag{19}$$

so that the Fisher Information in the network response is simply the sum of response SNRs along individual left eigenvectors. Although this case provides useful intuition, the assumption that Γ is diagonal places strong restrictions on the dynamics which may not be applicable to neural circuits, for example that the eigenvectors are orthogonal (following a whitening operation with respect to the input covariance $\Sigma_{\boldsymbol{\eta}}$, see below). For such networks (also known as “normal" networks), it can be seen that the solution which maximizes the linear Fisher Information in Equation (19) is to align the left eigenvector with the longest decay time constant τ_k with the linear discriminant of the instantaneous sensory input, so that $\mathcal{I}_F = \mathbf{g}' \cdot \Sigma_{\boldsymbol{\eta}}^{-1} \mathbf{g}' 2\tau_k$, much as in our analysis of single eigenmodes.

To show that a diagonal Γ implies that dynamics are normal following a whitening operation, we first note that $\Gamma_{ij} = (\tilde{V}^{-1} \Sigma_{\boldsymbol{\eta}} \tilde{V}^{-T})_{ij} / (\tau_i + \tau_j)$ can only be diagonal if $\tilde{V}^{-1} \Sigma_{\boldsymbol{\eta}} \tilde{V}^{-T}$ is diagonal (except

in the pathological case where $\tau_i + \tau_j = \infty$ for all $i \neq j$). Defining $X = \tilde{V}^{-1}\sqrt{\Sigma_{\boldsymbol{\eta}}}$, we therefore see that $XX^T = 2\Gamma\mathrm{diag}(\boldsymbol{\tau})$ is diagonal, which means that the rows of X form an orthogonal basis. The matrix $X^{-1} = \sqrt{\Sigma_{\boldsymbol{\eta}}}^{-1}\tilde{V}$ is the matrix of right eigenvectors whitened with respect to the input covariance $\Sigma_{\boldsymbol{\eta}}$, which must have orthogonal columns since $(XX^T)^{-1} = X^{-T}X^{-1} = (2\Gamma\mathrm{diag}(\boldsymbol{\tau}))^{-1}$ is diagonal. Thus, the input-whitened dynamical modes (i.e., the columns of $\sqrt{\Sigma_{\boldsymbol{\eta}}}^{-1}\tilde{V}$) must form a normal dynamical system whenever Γ is diagonal. In the following section, the terms normal and non-normal refer to the normality of dynamics following such a whitening operation.

3.4 Linear Fisher Information for Non-Normal Networks

Networks in which the eigenvectors of the Jacobian are not orthogonal are known as “non-normal" networks (Ganguli et al., 2008; Goldman, 2009; Murphy and Miller, 2009). We now study how non-normal network dynamics influence information integration and transmission (technically, non-normal following a whitening operation, see above). Our main finding is that non-normal dynamics can enhance the linear Fisher Information of network responses by a factor of up to N (the number of neurons in the network). These findings form the basis of the results presented in Figure S1. We note that closely related findings have been presented previously (Ganguli et al., 2008; Goldman, 2009). To arrive at these results, we first analyze an arbitrary two-dimensional non-normal system, then use the optimal solution obtained in this 2-dimensional case to motivate a specific class of N-dimensional networks which achieve the desired N-fold improvement in information transmission.

To gain intuition into how non-normality of network dynamics affects linear Fisher Information, we perturb the solution obtained for the normal network by adding a single pair off-diagonal elements $\Gamma_{ab} = \Gamma_{ba}$ to Γ. This perturbed system corresponds a network in which only a two-dimensional plane exhibits non-normal dynamics, with the remaining eigenvectors forming an orthogonal basis. This system has effective covariance matrix $\Sigma_{\text{eff}} = \Sigma_{\text{diag}} + \Gamma_{ab}\left(\tilde{\mathbf{v}}_a^R\left(\tilde{\mathbf{v}}_b^R\right)^T + \tilde{\mathbf{v}}_b^R\left(\tilde{\mathbf{v}}_a^R\right)^T\right)$, where Σ_{diag} is the effective covariance matrix for the unperturbed system. This covariance matrix can be inverted exactly using the Sherman-Morrison matrix inversion identity:

$$\Sigma_{\text{eff}}^{-1} = \Sigma_{\text{diag}}^{-1} + \frac{\Gamma_{ab}^2}{\Gamma_{aa}\Gamma_{bb} - \Gamma_{ab}^2}\left[\frac{1}{\Gamma_{aa}}\tilde{\mathbf{v}}_a^L\left(\tilde{\mathbf{v}}_a^L\right)^T + \frac{1}{\Gamma_{bb}}\tilde{\mathbf{v}}_b^L\left(\tilde{\mathbf{v}}_b^L\right)^T - \frac{1}{\Gamma_{ab}}\left(\tilde{\mathbf{v}}_a^L\left(\tilde{\mathbf{v}}_b^L\right)^T + \tilde{\mathbf{v}}_b^L\left(\tilde{\mathbf{v}}_a^L\right)^T\right)\right]. \tag{20}$$

This result can then be used to obtain the linear Fisher Information of the perturbed system via Equations (17, 19):

$$\mathcal{I}_F = \sum_i \frac{1}{\Gamma_{ii}}\left(\mathbf{g}'\cdot\tilde{\mathbf{v}}_i^L\right)^2 + \frac{\Gamma_{ab}^2}{\Gamma_{aa}\Gamma_{bb} - \Gamma_{ab}^2}\left[\frac{1}{\Gamma_{aa}}\left(\mathbf{g}'\cdot\tilde{\mathbf{v}}_a^L\right)^2 + \frac{1}{\Gamma_{bb}}\left(\mathbf{g}'\cdot\tilde{\mathbf{v}}_b^L\right)^2 - 2\frac{1}{\Gamma_{ab}}\left(\mathbf{g}'\cdot\tilde{\mathbf{v}}_a^L\right)\left(\mathbf{g}'\cdot\tilde{\mathbf{v}}_b^L\right)\right]. \tag{21}$$

By rearranging this expression, we can make explicit the information contained in the non-normal plane of dynamics (given in the second term below):

$$\mathcal{I}_F = \sum_{i\neq a,b} \frac{1}{\Gamma_{ii}}\left(\mathbf{g}'\cdot\tilde{\mathbf{v}}_i^L\right)^2 + \frac{1}{1 - \frac{\Gamma_{ab}^2}{\Gamma_{aa}\Gamma_{bb}}}\left[\frac{\left(\mathbf{g}'\cdot\tilde{\mathbf{v}}_a^L\right)^2}{\Gamma_{aa}} + \frac{\left(\mathbf{g}'\cdot\tilde{\mathbf{v}}_b^L\right)^2}{\Gamma_{bb}} - 2\frac{\Gamma_{ab}}{\Gamma_{aa}\Gamma_{bb}}\left(\mathbf{g}'\cdot\tilde{\mathbf{v}}_a^L\right)\left(\mathbf{g}'\cdot\tilde{\mathbf{v}}_b^L\right)\right]. \tag{22}$$

To understand how the non-normal component of the Fisher Information depends on the relative alignment of eigenvectors and their time constants we define $D_{ab} = \left(\tilde{\mathbf{v}}_a^L\right)^T\Sigma_{\boldsymbol{\eta}}\tilde{\mathbf{v}}_b^L$, so that $\Gamma_{ab} = D_{ab}/(\tau_a + \tau_b)$. We then introduce the two dimensionless quantities $\beta = \tau_b/\tau_a$ and $\kappa = \left[\left(\mathbf{g}'\cdot\tilde{\mathbf{v}}_b^L\right)^2/D_{bb}\right]/\left[\left(\mathbf{g}'\cdot\tilde{\mathbf{v}}_a^L\right)^2/D_{aa}\right]$. The term D_{ab} quantifies the degree of non-orthogonality of the eigenvector pair a, b under a metric induced by the input covariance (or equivalently the covariance of sensory input following projection onto the two eigenvectors). β quantifies the relative time

constants of the two eigenmodes, and κ quantifies the relative signal to noise ratio of sensory input projected onto the two left eigenvectors. Without loss of generality, we may assume that $\tau_a \geq \tau_b$, so that $\beta \leq 1$.

Inserting these definitions into Equation (22) gives:

$$\mathcal{I}_F = \sum_{i \neq a,b} 2\tau_i \frac{\left(\mathbf{g}' \cdot \tilde{\mathbf{v}}_i^L\right)^2}{D_{ii}} + 2\tau_a \frac{\left(\mathbf{g}' \cdot \tilde{\mathbf{v}}_a^L\right)^2}{D_{aa}} \frac{1 + \kappa\beta - 4\sqrt{\kappa}\frac{\beta}{1+\beta}\frac{D_{ab}}{\sqrt{D_{aa}D_{bb}}}}{1 - 4\frac{D_{ab}^2}{D_{aa}D_{bb}}\frac{\beta}{(1+\beta)^2}}. \tag{23}$$

As $D_{ab} \to 0$, the solution for the normal system is recovered (Equation (19)). However, if both $\kappa \to 1$ and $\frac{D_{ab}}{\sqrt{D_{aa}D_{bb}}} \to 1$ then the Fisher Information becomes $\mathcal{I}_F = \sum_{i \neq a,b} 2\tau_i \frac{\left(\mathbf{g}' \cdot \tilde{\mathbf{v}}_i^L\right)^2}{D_{ii}} + 2\tau_a \frac{\left(\mathbf{g}' \cdot \tilde{\mathbf{v}}_a^L\right)^2}{D_{aa}}(1+\beta)$. Then as $\beta \to 1$ the Fisher Information becomes $\mathcal{I}_F = \sum_{i \neq a,b} 2\tau_i \frac{\left(\mathbf{g}' \cdot \tilde{\mathbf{v}}_i^L\right)^2}{D_{ii}} + 4\tau_a \frac{\left(\mathbf{g}' \cdot \tilde{\mathbf{v}}_a^L\right)^2}{D_{aa}}$. Taking this set of limits corresponds to the case where $\tilde{\mathbf{v}}_a^L \to \tilde{\mathbf{v}}_b^L$ and $\tau_a \to \tau_b$. The linear Fisher Information is then maximized by setting $\tilde{\mathbf{v}}_a^L = \Sigma_{\boldsymbol{\eta}}^{-1}\mathbf{g}'$, in which case both left eigenvectors in the non-normal plane are aligned to the input linear discriminant while all other left eigenvectors are orthogonal. The total response information for such a network is $\mathcal{I}_F = \mathbf{g}' \cdot \Sigma_{\boldsymbol{\eta}}^{-1}\mathbf{g}' 4\tau_a$, which is twice that achievable by any normal network whose longest time constant is τ_a (see Figure S1B for a numerical validation of this result). It is noteworthy that the limit taken here yields a defective matrix $\tilde{J}$, i.e. one which has fewer distinct eigenvectors than it has dimensions N. We next show that, by constructing a maximally-defective matrix, i.e. one which has just one eigenvector repeated N times, it is possible to achieve an N-fold improvement in linear Fisher Information relative to an optimal normal network.

To extend this two-dimensional example to the N-dimensional case, we construct a network in which non-normal dynamics produce an N-fold increase in response information. Motivated by our signal processing analysis, we search for cases in which there exists a pair of projections $\mathbf{w}$ of the neural response $\delta\mathbf{r} \equiv \mathbf{r} - \mathbf{r}_{SS} = \int_0^\infty e^{J\tau}\Phi'\mathbf{u}(s, t-\tau)d\tau$ and $\mathbf{n}$ of the sensory input $\mathbf{u}(s,t)$ such that:

$$\mathbf{w} \cdot \delta\mathbf{r} = \int_0^\infty f(\tau)\mathbf{n} \cdot \mathbf{u}(s, t-\tau)d\tau. \tag{24}$$

for some yet-to-be-determined function $f(t)$. In such a case the SNR of network responses projected onto $\mathbf{w}$ is given by Equation (2) with $T \to \infty$.

We can immediately identify one solution to Equation (24), which is $\mathbf{w} = \mathbf{v}_j^L$, $\mathbf{n} = \tilde{\mathbf{v}}_j^L$, $f(t) = e^{\lambda_j t}$. This recovers our single-eigenvector analysis. To construct a second case, we consider a network with $J_{ij} = \lambda\delta_{ij} + \omega\delta_{i,j-1}$, which corresponds to a delay line in which units have decay time constants $\tau_i = -1/\lambda$ and feedforward weights ω (by feedforward, we mean that the weights are ordered along the delay line). It can be verified that this matrix has only one distinct eigenvalue λ and one distinct eigenvector $\left(\mathbf{v}^L\right)_i = \delta_{iN}$. Then $\left[e^{tJ}\right]_{ij} = \delta_{j \geq i}\frac{(\omega t)^{j-i}}{(j-i)!}e^{\lambda t}$ (as can be shown using the power series definition of a matrix exponential). Thus, Equation (24) becomes:

$$\sum_{i=1}^N \sum_{j=i}^N w_i \int_0^\infty \frac{(\omega\tau)^{j-i}}{(j-i)!}e^{\lambda\tau}\Phi'_{jj}u_j(s, t-\tau)d\tau = \sum_{j=1}^N \int_0^\infty f(\tau)n_j u_j(s, t-\tau)d\tau. \tag{25}$$

There does not in general exist an $\mathbf{n}$ and f which satisfy this equation, but in the limit $\omega \to \infty$ a solution exists because $\sum_{j=i}^N \frac{(\omega\tau)^{j-i}}{(j-i)!}e^{\lambda\tau}\Phi'_{jj}u_j(s, t-\tau) \to \frac{(\omega\tau)^{N-i}}{(N-i)!}e^{\lambda\tau}\Phi'_{NN}u_N(s, t-\tau)$. This gives the equation:

$$\sum_{i=1}^N w_i \int_0^\infty \frac{(\omega\tau)^{N-i}}{(N-i)!}e^{\lambda\tau}\Phi'_{NN}u_N(s, t-\tau)d\tau = \sum_{j=1}^N \int_0^\infty f(\tau)n_j u_j(s, t-\tau)d\tau. \tag{26}$$

We can then identify a second solution to Equation (24), which is $n_i = \delta_{iN}$ and $f(t) = \sum_{i=1}^{N} w_i \frac{(\omega t)^{N-i}}{(N-i)!} e^{\lambda t} \Phi'_{NN}$. Thus, while we are free to choose any set of readout weights $\mathbf{w}$, only the input to the Nth neuron can be recovered from the output of such a network regardless of the readout weights we choose. In this case, the readout weights $\mathbf{w}$ determine the temporal filter $f(t)$ applied to the Nth neuron's input, with different choices of $\mathbf{w}$ allowing different functions of the input history to be recovered.

Having identified this solution, we next proceed to maximize the SNR of responses along $\mathbf{w}$. To optimize response SNR along $\mathbf{w}$, we need to maximize both $\mathrm{SNR}_{\mathrm{input}}(\mathbf{n})$ and $I_\infty(f)$ as defined in Equation (2). $I_\infty(f)$ can be maximized by choosing the appropriate readout weights $\mathbf{w}$ as follows:

$$I_\infty(f) = \frac{\left[\int_0^\infty f(t)dt\right]^2}{\int_0^\infty f^2(t)dt} = \frac{\left[\sum_{i=1}^{N} w_i \frac{\omega^{N-i}}{(-\lambda)^{N-i+1}}\right]^2}{\sum_{i,j=1}^{N} w_i w_j \frac{\omega^{2N-i-j}}{(-2\lambda)^{2N-i-j+1}} \frac{(2N-i-j)!}{(N-i)!(N-j)!}} \equiv \frac{1}{-\lambda} \frac{\left[\bar{\mathbf{w}} \cdot \mathbf{1}\right]^2}{\bar{\mathbf{w}} \cdot S \bar{\mathbf{w}}} \tag{27}$$

where we have defined $w_i = \left(-\frac{\lambda}{\omega}\right)^{N-i} \bar{w}_i$ and $S_{ij} = 2^{-(2N-i-j+1)} \frac{(2N-i-j)!}{(N-i)!(N-j)!}$ and $\mathbf{1}$ is a vector of ones. The Cauchy-Schwarz inequality then yields $I_\infty(f) \leq (-\lambda)^{-1} \mathbf{1}^T S^{-1} \mathbf{1} = (-\lambda)^{-1} \sum_{i,j=1}^{N} \left(S^{-1}\right)_{ij}$, with the upper bound achieved when $\bar{\mathbf{w}} = S^{-1}\mathbf{1}$. We find numerically that $\sum_{i,j=1}^{N} \left(S^{-1}\right)_{ij} = 2N$, so that $I_\infty(f) = (-\lambda)^{-1} 2N$, revealing an N-fold increase in temporal integration through non-normal dynamics (because λ is the only eigenvalue of J, a normal network could obtain at best $I_\infty(f) = 2(-\lambda)^{-1}$). Figure S1H shows the temporal filter $f(t)$ that results from this choice of weights when $N = 16$.

We now ask how to maximize the second factor in our signal processing analysis, $\mathrm{SNR}_{\mathrm{input}}(\mathbf{n})$. Because the input projection integrated by the above network is $n_i = \delta_{iN}$, $\mathrm{SNR}_{\mathrm{input}}(\mathbf{n})$ is maximized when the linear discriminant of sensory input is aligned to the Nth element of the delay line. However, orthogonal transformations of this delay line, $J \to UJU^T$ with $U^T = U^{-1}$, change the projection of sensory input integrated by the network as $\mathbf{n} \to U\mathbf{n}$, but do not otherwise affect the results. Thus, $\mathrm{SNR}_{\mathrm{input}}(\mathbf{n})$ is maximized by rotating the delay line in neural space so that $\mathbf{n}$ aligns with the linear discriminant of sensory input, while $I_\infty(f)$ is maximized by the appropriate choice of readout weights $\mathbf{w}$ as described in the preceding paragraph (which must also be rotated, $\mathbf{w} \to U\mathbf{w}$). This rotated delay line corresponds to a "functionally feedforward" dynamic (Goldman, 2009) and the integrative properties of such delay line architectures have been studied previously (Ganguli et al., 2008). The Jacobian J introduced here is a defective matrix, i.e. it has only one eigenvector ($\mathbf{v}_i^L = \mathbf{n}$) and one eigenvalue ($\lambda$), and therefore is consistent with the result of the two-dimensional case in which information increases when eigenvectors become more aligned and eigenvalues simultaneously become more similar. Moreover, the optimization of $\mathrm{SNR}_{\mathrm{input}}(\mathbf{n})$ requires that this left eigenvector is aligned to the input linear discriminant, demonstrating that the optimal non-normal network is one in which all left eigenvectors are aligned to the input linear discriminant and have identical time constants. Figure S1G-J show the response information computed from networks with varying number of units N and feedforward weight ω.

4 The Role of Input Temporal Correlations

We next extend our analysis of information transmission through recurrent networks to handle temporally correlated input noise. We first consider how to optimally discriminate stimuli based on the temporally correlated input they produce (or more generally, input with time-dependent second order statistics), and then show how the response SNR and linear Fisher information of a recurrent network receiving multivariate Ornstein-Uhlenbeck input depend on alignment of dynamical modes with temporally correlated input patterns. We find that temporal correlations can substantially modify the optimal readout of sensory input, requiring a time-varying linear discriminant and reorienting the

discriminant vector to avoid input dimensions with strong temporal correlations. However, under certain assumptions, analytical solutions can be obtained for networks driven by Ornstein-Uhlenbeck input which involve only minor modifications to those driven by temporally uncorrelated input.

4.1 Signal Processing Analysis with Temporal Correlations

To extend our signal processing analysis to handle temporal correlations, we modify our previous model for the network input $\mathbf{u}(s,t) = \mathbf{g}(s) + \boldsymbol{\eta}(t)$ by allowing $\boldsymbol{\eta} \sim N(0, \Sigma_{\boldsymbol{\eta}}(t,t'))$ where $\Sigma_{\boldsymbol{\eta}}(t,t') = \langle \boldsymbol{\eta}(t)\boldsymbol{\eta}^T(t')\rangle$ is an arbitrary (stimulus-independent) space-time covariance matrix. Let $Q(t,t')$ be the space-time inverse of $\Sigma_{\boldsymbol{\eta}}(t,t')$, i.e. the solution to the integral equation $\int_0^T dt' Q(t,t')\Sigma_{\boldsymbol{\eta}}(t',t'')dt' = \delta(t-t'')$ (note that $\int_0^T dt' \Sigma_{\boldsymbol{\eta}}(t,t')Q^T(t',t'')dt' = \delta(t-t'')$ because $\Sigma_{\boldsymbol{\eta}}(t,t') = \Sigma_{\boldsymbol{\eta}}^T(t',t)$). On each trial of stimulus s, the input $\mathbf{u}$ is drawn from the probability distribution

$$p(\mathbf{u}|s) = \frac{1}{Z}\exp\left[-\int_0^T dt \int_0^T dt' (\mathbf{u}(t') - \mathbf{g}(s))^T Q(t',t)(\mathbf{u}(t) - \mathbf{g}(s))\right]$$

where Z is a normalizing constant. Following our analysis for the temporally uncorrelated case, we now find the MAP solution for discrimination between two stimuli s_1, s_2:

$$\begin{aligned}
&\log p(s_1|\mathbf{u}) - \log p(s_2|\mathbf{u}) = \log p(s_1) - \log p(s_2) \\
&+ \int_0^T dt \int_0^T dt' [\mathbf{u}(t')^T Q(t',t)(\mathbf{g}(s_1) - \mathbf{g}(s_2)) + (\mathbf{g}(s_1) - \mathbf{g}(s_2))^T Q(t',t)\mathbf{u}(t) \\
&- \mathbf{g}^T(s_1)Q(t',t)\mathbf{g}(s_1) - \mathbf{g}^T(s_2)Q(t',t)\mathbf{g}(s_2)] \\
&= c(T) + \int_0^T \mathbf{w}_{LD}(t)\cdot\mathbf{u}(t)dt
\end{aligned}$$

where $\mathbf{w}_{LD}^T(t) = (\mathbf{g}(s_1) - \mathbf{g}(s_2))^T \int_0^T (Q(t',t) + Q^T(t,t'))dt'$ and $c(T)$ is a constant with respect to $\mathbf{u}$. The MAP solution is to project the input $\mathbf{u}$ onto a time-varying discriminant vector $\mathbf{w}_{LD}$ and integrate this projection over time. This solution is similar to that obtained when assuming temporally uncorrelated input, with two important modifications. First, the linear discriminant varies over the course of the trial (setting $Q(t,t') = \Sigma_{\boldsymbol{\eta}}^{-1}\delta(t-t')$ recovers the solution to the temporally uncorrelated case). Second, the linear discriminant depends on the space-time precision matrix Q, with contributions from both spatial and temporal covariance (as we show below for the special case of an Ornstein-Uhlenbeck process). Note that, because we have assumed arbitrary space-time covariance, the linear discriminant derived above may be shaped by non-stationary variance as well as temporal correlations. Imposing further restrictions on $\Sigma_{\boldsymbol{\eta}}$ and Q (e.g., time translation invariance) would enable further investigation of the role of each type of variability. Unlike the temporally uncorrelated case, this MAP solution is typically not achievable by a linear network, but could in principle be achieved by a nonlinear network or may be well approximated using non-normal dynamics in a linear network (Ganguli et al., 2008).

We next compute the SNR obtained via this optimal projection and filtering. Because the space-time vector $\mathbf{u}$ is drawn from a normal distribution with stimulus-independent noise, this SNR fully captures the discrimination performance of an ideal observer of sensory input, as was the case for temporally uncorrelated input. Computing the mean and variance of the scalar output $d(T) = \int_0^T \mathbf{w}_{LD}(t)\cdot\mathbf{u}(t)dt$ gives $\langle d(T)\rangle_s = \Delta\mathbf{g}^T \int_0^T\int_0^T (Q(t',t) + Q^T(t,t'))dtdt'\mathbf{g}(s)$ and

$$\mathrm{var}(d(T)) = \Delta\mathbf{g}^T \int_0^T\int_0^T\int_0^T\int_0^T (Q(t',t)+Q^T(t,t'))\Sigma_{\boldsymbol{\eta}}(t,\tau)(Q(\tau',\tau)+Q^T(\tau,\tau'))^T dtdt'd\tau d\tau' \Delta\mathbf{g}$$

where $\Delta\mathbf{g} = \mathbf{g}(s_1) - \mathbf{g}(s_2)$. Applying the definition of Q simplifies this expression to $\mathrm{var}(d(T)) =$

$2(\langle d(T)\rangle_{s=s_1} - \langle d(T)\rangle_{s=s_2})$. The signal to noise ratio $\mathrm{SNR}^2(d(T)) = (\langle d(T)\rangle_{s=s_1} - \langle d(T)\rangle_{s=s_2})^2/\mathrm{var(d(T))}$ is therefore

$$\mathrm{SNR}^2(d(T)) = \frac{1}{2}(\mathbf{g}(s_1) - \mathbf{g}(s_2))^T \left[\int_0^T \int_0^T (Q(t', t) + Q^T(t, t'))dt dt'\right] (\mathbf{g}(s_1) - \mathbf{g}(s_2))$$

Thus, the performance of an optimal decoder of stimuli from temporally correlated input depends on the alignment of the time integral of the space-time precision matrix $\int_0^T \int_0^T (Q(t', t) + Q^T(t, t'))dt dt'$ with the input signal vector $\mathbf{g}(s_1) - \mathbf{g}(s_2)$. Temporally uncorrelated input noise is a special case of this solution, obtained by inserting $Q(t, t') = \delta(t - t')\Sigma_{\boldsymbol{\eta}}^{-1}$, which gives $\mathrm{SNR}^2(d(T)) = (\mathbf{g}(s_1) - \mathbf{g}(s_2))^T \Sigma_{\boldsymbol{\eta}}^{-1} (\mathbf{g}(s_1) - \mathbf{g}(s_2))T$. In the temporally correlated case (or more generally, the case with time-varying second order statistics), the available information can be large if $Q(t', t) + Q^T(t, t')$ has large projection onto $\mathbf{g}(s_1) - \mathbf{g}(s_2)$ at any pair of time points t, t', i.e. if there exists a pair of time points at which inputs are precisely coordinated along the direction separating the two stimulus means. In the MAP solution, this information is extracted by weighting these time points strongly in the time-varying linear discriminant.

4.2 Response SNR of Recurrent Networks Driven by Ornstein-Uhlenbeck Noise

While the above result provides insight into the impact of temporal correlations on the optimal decoder of sensory input, $Q(t, t')$ cannot be found analytically for most choices of $\Sigma_{\boldsymbol{\eta}}(t, t')$ and so the optimal time-varying linear discriminant and its associated SNR must be found numerically. However, analytical expressions can be found for the response SNR of a recurrent network that integrates certain forms of temporally correlated input (typically suboptimally). Using a similar analysis to the temporally uncorrelated case, we derive the response SNR of a linearized network driven by multivariate Ornstein-Uhlenbeck input defined by $\tau_\eta \dot{\boldsymbol{\eta}} = A\boldsymbol{\eta} + \sqrt{2\tau_\eta \Sigma_{\mathbf{n}}}\mathbf{n}(t)$ where $\mathbf{n}(t) \sim N(0, I)$ is temporally uncorrelated and stimulus-independent Gaussian noise. Assuming stationary state, $\boldsymbol{\eta}$ has mean $\langle \boldsymbol{\eta}(t)\rangle = 0$ and cross-covariance $\langle (\boldsymbol{\eta}(t) - \langle \boldsymbol{\eta}(t)\rangle)(\boldsymbol{\eta}(t') - \langle \boldsymbol{\eta}(t')\rangle)^T\rangle = \Sigma_{\boldsymbol{\eta}}(t, t') = \frac{2}{\tau_\eta}\int_{-\infty}^{\min(t,t')} e^{(t-\tau)A/\tau_\eta}\Sigma_{\mathbf{n}} e^{(t'-\tau)A^T/\tau_\eta} d\tau$. The response of a network linearized about stimulus input $\mathbf{g}(s)$ has dynamics[1] $\delta\dot{\mathbf{r}} = J\delta\mathbf{r} + \boldsymbol{\eta}(t)$ and stationary state response covariance $\Sigma = 2\tau_\eta A^{-1}\Sigma^{\mathrm{uncorrelated}} A^{-T}$, where $\Sigma^{\mathrm{uncorrelated}} = \int_0^\infty e^{Jt}\Sigma_{\mathbf{n}} e^{J^T t} dt$ is the response covariance that would be obtained if the network were driven by the temporally uncorrelated input $\sqrt{\Sigma_{\mathbf{n}}}\mathbf{n}$ rather than the correlated input $\boldsymbol{\eta}$. To see this, note that $\boldsymbol{\eta}(t) = \int_{-\infty}^t e^{A(t-\tau)/\tau_\eta}\sqrt{2\Sigma_{\mathbf{n}}/\tau_\eta}\mathbf{n}(\tau)d\tau$ and $\delta\mathbf{r}(t) = \int_{-\infty}^t e^{J(t-\tau)}\boldsymbol{\eta}(\tau)d\tau = \int_{-\infty}^t d\tau e^{J(t-\tau)} \int_{-\infty}^\tau e^{A(\tau-s)/\tau_\eta}\sqrt{2\Sigma_{\mathbf{n}}/\tau_\eta}\mathbf{n}(s)ds$. Thus,

$$\Sigma = \frac{2}{\tau_\eta}\int_{-\infty}^t d\tau \int_{-\infty}^t d\tau' \int_{-\infty}^\tau ds \int_{-\infty}^{\tau'} ds' e^{J(t-\tau)} e^{A(\tau-s)/\tau_\eta}\Sigma_{\mathbf{n}} e^{A^T(\tau'-s')/\tau_\eta} e^{J^T(t-\tau')}\delta(\tau - \tau')$$

which can be solved straightforwardly to achieve the stated result since s, s', τ' can all be integrated independently.

The response SNR of network output along a direction $\mathbf{w}$ is,

$$\mathrm{SNR}^2(\mathbf{w}) \equiv \frac{(\mathbf{w}\cdot\mathbf{r}')^2}{\mathbf{w}\cdot\Sigma\mathbf{w}} = \frac{1}{2\tau_\eta}\frac{(\mathbf{w}\cdot J^{-1}\mathbf{g}')^2}{\mathbf{w}^T A^{-1}\left[\int_0^\infty e^{Jt}\Sigma_{\mathbf{n}} e^{J^T t}dt\right] A^{-T}\mathbf{w}}$$

Setting $\mathbf{w} = \mathbf{v}_k^L$ (where $\mathbf{v}_k^L$ is a left eigenvector of J) would simplify the numerator but not denominator and setting $\mathbf{w} = A^T\mathbf{v}_k^L$ would simplify the denominator but not the numerator, so that a full simplification similar to that obtained for temporally uncorrelated input is not possible (Equations

[1] To reduce notational clutter, we drop the tildes used in previous sections, so these results strictly apply to linear rather than linearized networks.

(14)-(16)). However, in the special case where A and J commute[2], the SNR simplifies to

$$\mathrm{SNR}^2(\mathbf{w}) = \frac{1}{2\tau_\eta} \frac{(\mathbf{w} \cdot J^{-1}\mathbf{g}')^2}{\mathbf{w}^T \left[\int_0^\infty e^{Jt} A^{-1} \Sigma_\mathbf{n} A^{-T} e^{J^T t} dt\right] \mathbf{w}}$$

so that setting $\mathbf{w} = \mathbf{v}_i^L$ gives

$$\mathrm{SNR}^2(\mathbf{v}_i^L) = \frac{(\mathbf{v}_i^L \cdot \mathbf{g}')^2}{\mathbf{v}_i^L \cdot A^{-1}\Sigma_\mathbf{n} A^{-T} \mathbf{v}_i^L} \frac{\tau_i}{\tau_\eta}$$

This result is identical to the one obtained for temporally uncorrelated input, but with the input covariance matrix $\Sigma_{\boldsymbol{\eta}} \rightarrow 2\tau_\eta A^{-1}\Sigma_\mathbf{n} A^{-T}$. For fixed $A, \mathbf{u}', \Sigma_\mathbf{n}, \tau_\eta, \tau_i$, this SNR is maximized when $\mathbf{v}_i^L \propto A^T \Sigma_\mathbf{n}^{-1} A \mathbf{g}'$, which gives $\mathrm{SNR}^2(\mathbf{v}_i^L) = \mathbf{g}' \cdot A^T \Sigma_\mathbf{n}^{-1} A \mathbf{g}' \frac{\tau_i}{\tau_\eta}$. Note that, in contrast to the solution obtained assuming temporally uncorrelated input, the matrix $A^{-1}\Sigma_\mathbf{n} A^{-T}$ is not the instantaneous covariance of the input noise $\boldsymbol{\eta}$, which is $\Sigma_{\boldsymbol{\eta}} \propto \int_0^\infty e^{At}\Sigma_\mathbf{n} e^{A^T t} dt$. Thus, rather than integrating the input dimension with greatest instantaneous SNR, the solution identified here involves integrating the input dimension with greatest information per unit time, which requires trading off the instantaneous SNR against the degree of temporal correlations along each direction.

4.3 Linear Fisher Information of Recurrent Networks Driven by Ornstein-Uhlenbeck Noise

The linear Fisher information of the network output is

$$\mathcal{I}_F = \mathbf{r}' \cdot \Sigma^{-1}\mathbf{r}' = 2\tau_\eta \mathbf{g}'^T J^{-T} \left[A^{-1} \int_0^\infty e^{Jt}\Sigma_\mathbf{n} e^{J^T t} dt A^{-T}\right]^{-1} J^{-1}\mathbf{g}' = \mathbf{g}'^T \Sigma_{\text{eff}}^{-1} \mathbf{g}'$$

where $\Sigma_{\text{eff}} = 2\tau_\eta J A^{-1} \left[\int_0^\infty e^{Jt}\Sigma_\mathbf{n} e^{J^T} dt\right] (JA^{-1})^T$. This effective covariance matrix satisfies the Lyapunov equation[3] $\bar{J}^{-1}\Sigma_{\text{eff}} + \Sigma_{\text{eff}}\bar{J}^{-T} + \bar{\Sigma}_\mathbf{n} = 0$ where $\bar{J} = (JA^{-1})J(JA^{-1})^{-1}$ and $\bar{\Sigma}_\mathbf{n} = \frac{2}{\tau_\eta}(JA^{-1}J^{-1})\Sigma_\mathbf{n}(JA^{-1}J^{-1})^T$. Although this result shares a strong resemblance to that obtained assuming temporally uncorrelated input, the modifications to the Lyapunov equation introduce a dependence of the effective input covariance $\bar{\Sigma}_\mathbf{n}$ on the recurrent dynamics J, so that our previous analyses cannot be straightforwardly applied. However, if J and A commute then $\bar{J} = J$ and $\bar{\Sigma}_\mathbf{n} = 2\tau_\eta A^{-1}\Sigma_\mathbf{n} A^{-T}$ (independent of J). In this special case the results of our analysis of temporally uncorrelated inputs, including the role of non-normality in shaping the linear Fisher information of network output, can be carried over straightforwardly by replacing Σ_η with $2\tau_\eta A^{-1}\Sigma_\mathbf{n} A^{-T}$. We note that although this special case is restrictive, it encompasses the case in which $A = -I$, as in previous studies (e.g., Hennequin et al., 2018). In that case, temporal correlations have no effect on response information or the optimal (linearized) network dynamics, other than scaling response information by a factor of τ_η^{-1}.

[2]The requirement that A and J commute may be relaxed to the more general condition that $\mathbf{w}$ is a left eigenvector of both J and A.

[3]This result follows from the Lyapunov equation $J\Sigma^{\text{uncorrelated}} + \Sigma^{\text{uncorrelated}} J^T + \Sigma_\mathbf{n} = 0$ and the fact that $\Sigma_{\text{eff}} = JA^{-1}\Sigma^{\text{uncorrelated}}(JA^{-1})^T$, where $\Sigma^{\text{uncorrelated}} = \int_0^\infty e^{Jt}\Sigma_\mathbf{n} e^{J^T t} dt$
